# Supplementary material for: Design, Synthesis and Biological Evaluation of Phenyl Urea Derivatives as IDO1 Inhibitors
Source: Molecules. 2020 Mar 23;25(6):1447. doi: 10.3390/molecules25061447 (PMC7144934; doi:10.3390/molecules25061447)
Supplement: Supplementary file 1 [file molecules-25-01447-s001.pdf]

Design, Synthesis and Biological Evaluation of Phenyl Urea Derivatives as IDO1 Inhibitors

Chuan Zhou [1], Fangfang Lai [2,*], Li Sheng [2,*], Xiaoguang Chen [2,*], Yan Li [2], Zhiqiang Feng [1,*]

[1] *Beijing Key Laboratory of Active Substance Discovery and Druggability Evaluation, Institute of Materia Medica, Chinese Academy of Medical Sciences and Peking Union Medical College, Beijing, 100050, China;* *zhouchuan@imm.ac.cn (C.Z.)*

[2] *State Key Laboratory of Bioactive Substance and Function of Natural Medicines, Institute of Materia Medica, Chinese Academy of Medical Sciences and Peking Union Medical College, Beijing, 100050, China;* *yanli@imm.ac.cn (Y.L.)*

* *Correspondence: fengzhq@imm.ac.cn (Z.F.); Tel.: +86-10-63189351 (Z.F.)*

^{1}H NMR, ^{13}C NMR and MS spectra of the synthesized compounds P2-P53

^{1}H NMR spectra of compound a

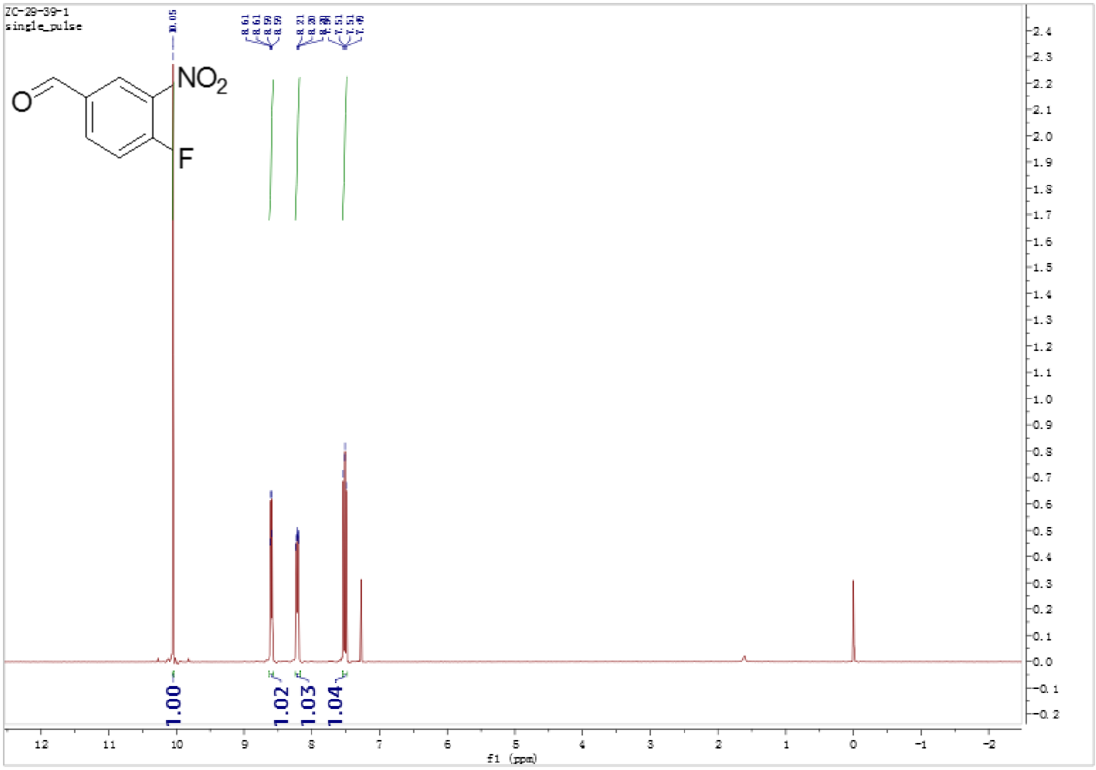

ESI-MS spectra of compound a

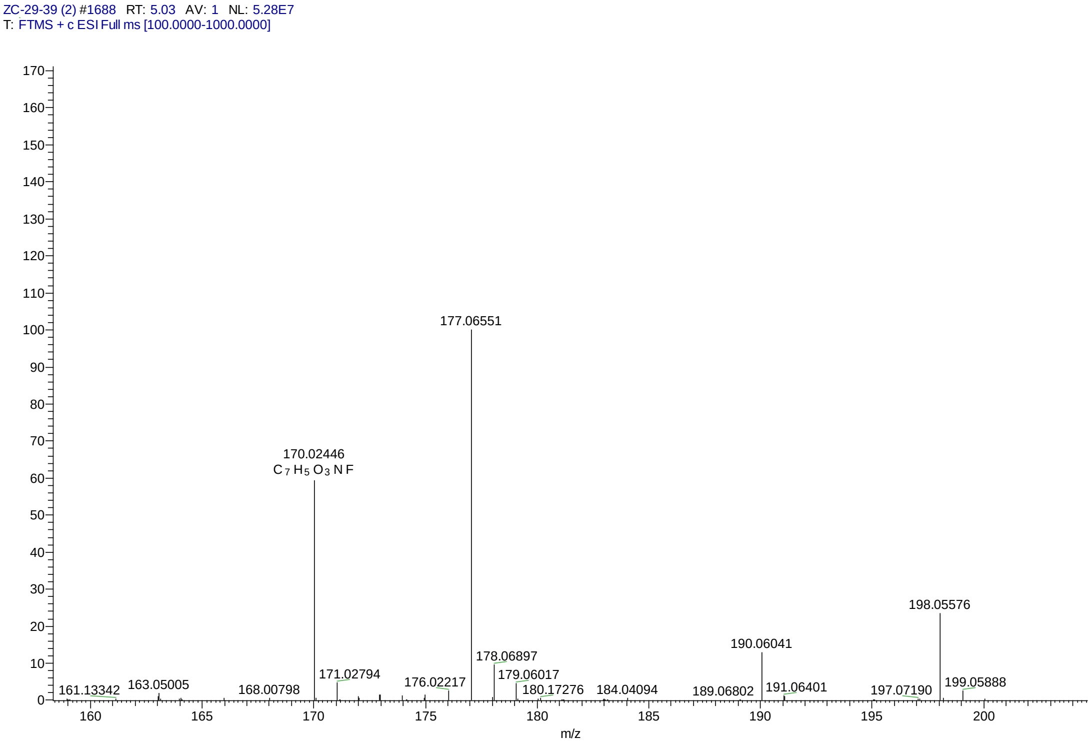

m/z	Theo. Mass	Delta (ppm)	RDB equiv.	Composition
170.02446	170.02480	-1.99	5.5	C7 H5 O3 N F

^{1}H NMR spectra of compound b

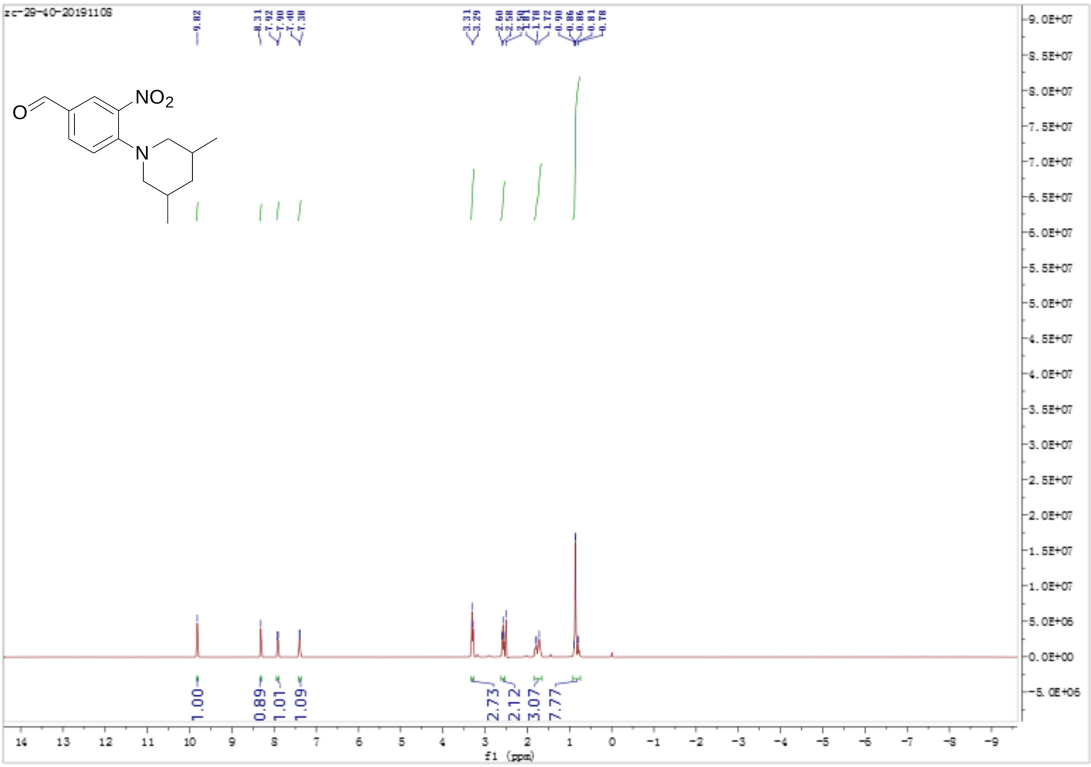

ESI-MS spectra of compound b

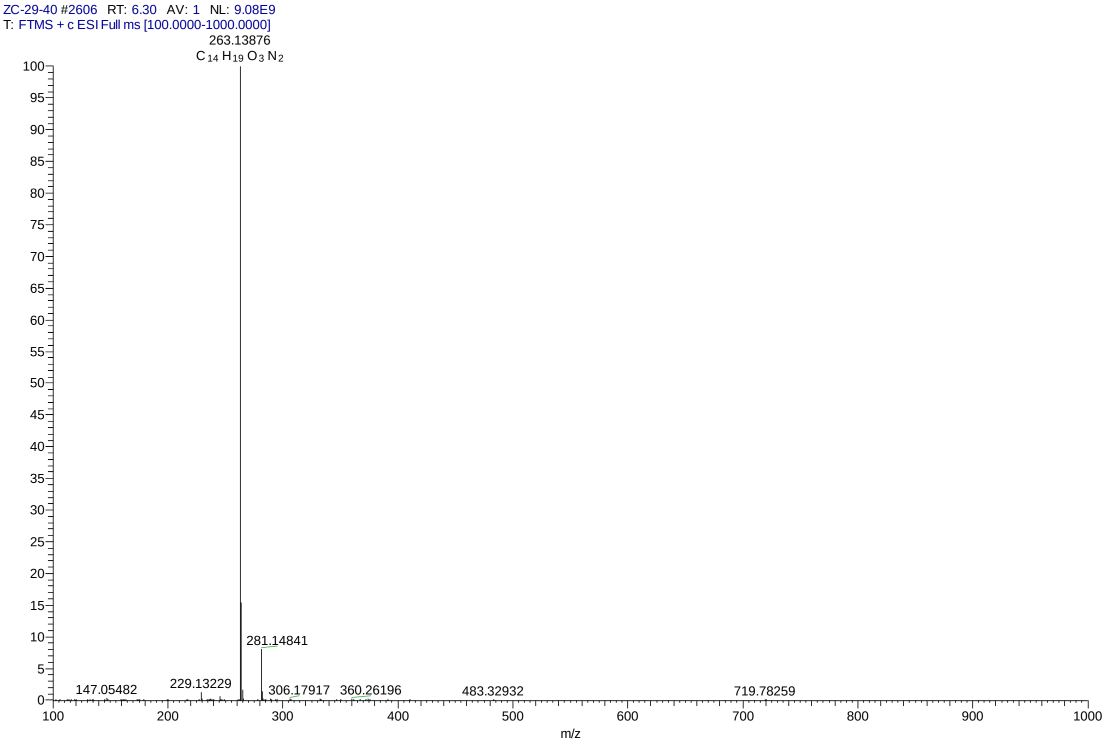

m/z	Theo. Mass	Delta (ppm)	RDB equiv.	Composition
263.13876	263.13902	-0.98	6.5	C14 H19 O3 N2

^{1}H NMR spectra of compound c

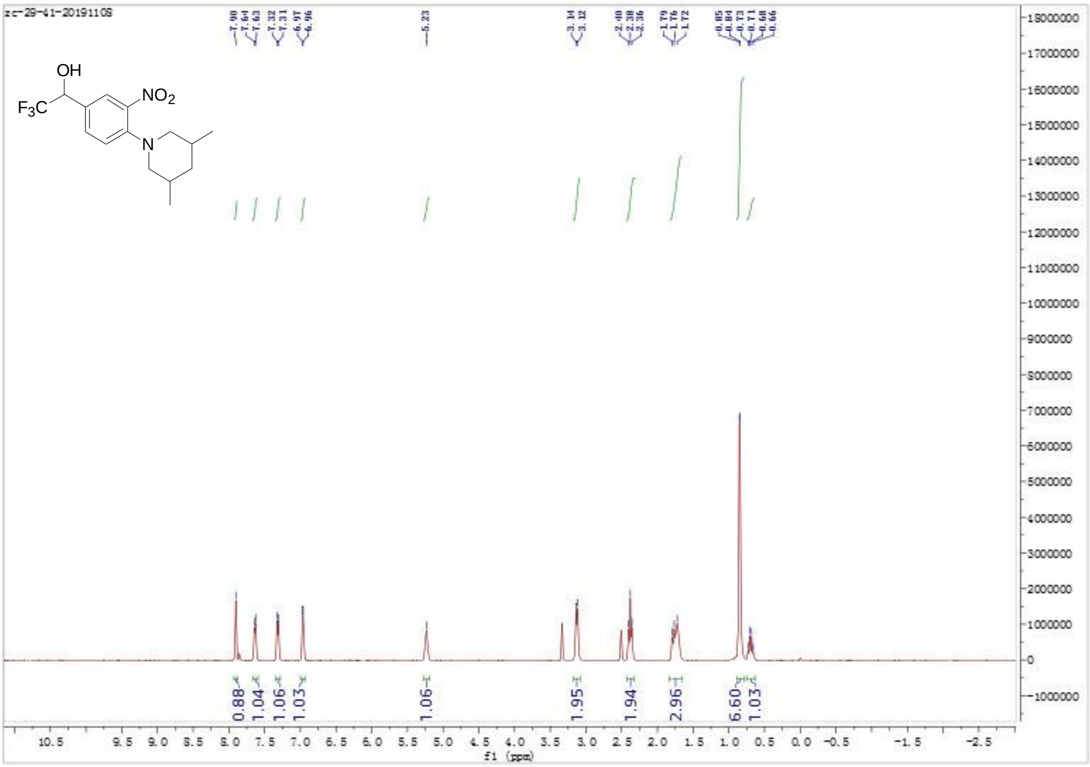

ESI-MS spectra of compound c

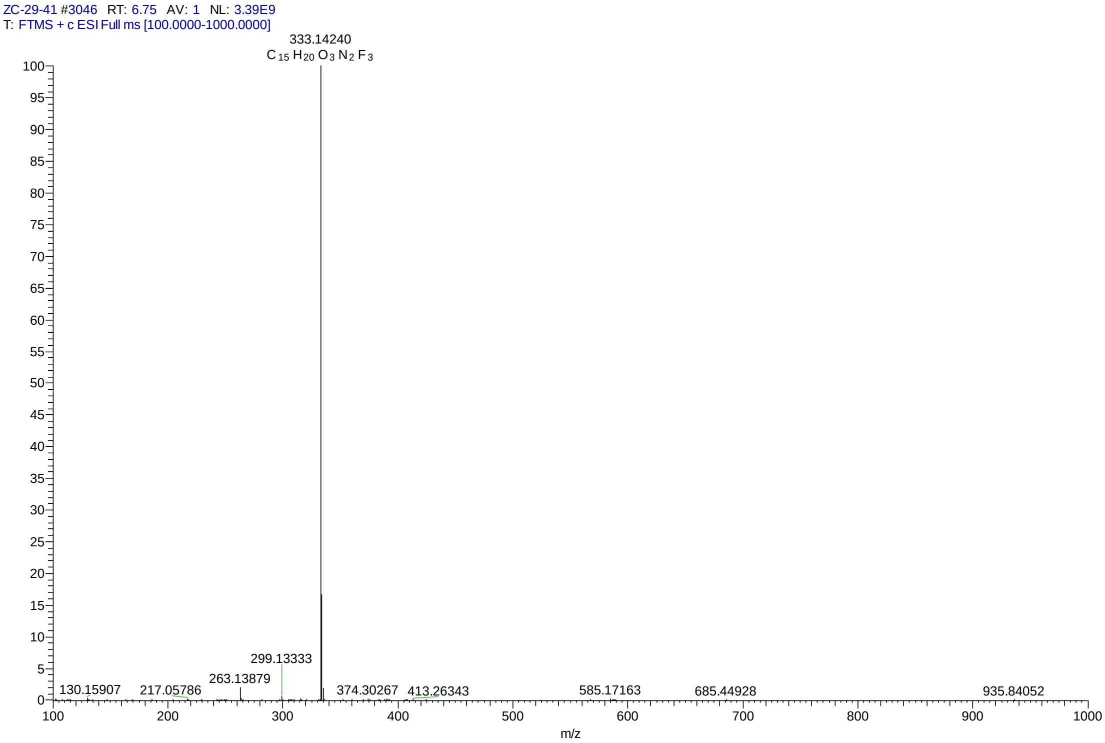

m/z	Theo. Mass	Delta (ppm)	RDB equiv.	Composition
333.14246	333.14205	1.22	5.5	C15 H20 O3 N2 F3

^{1}H NMR spectra of compound e

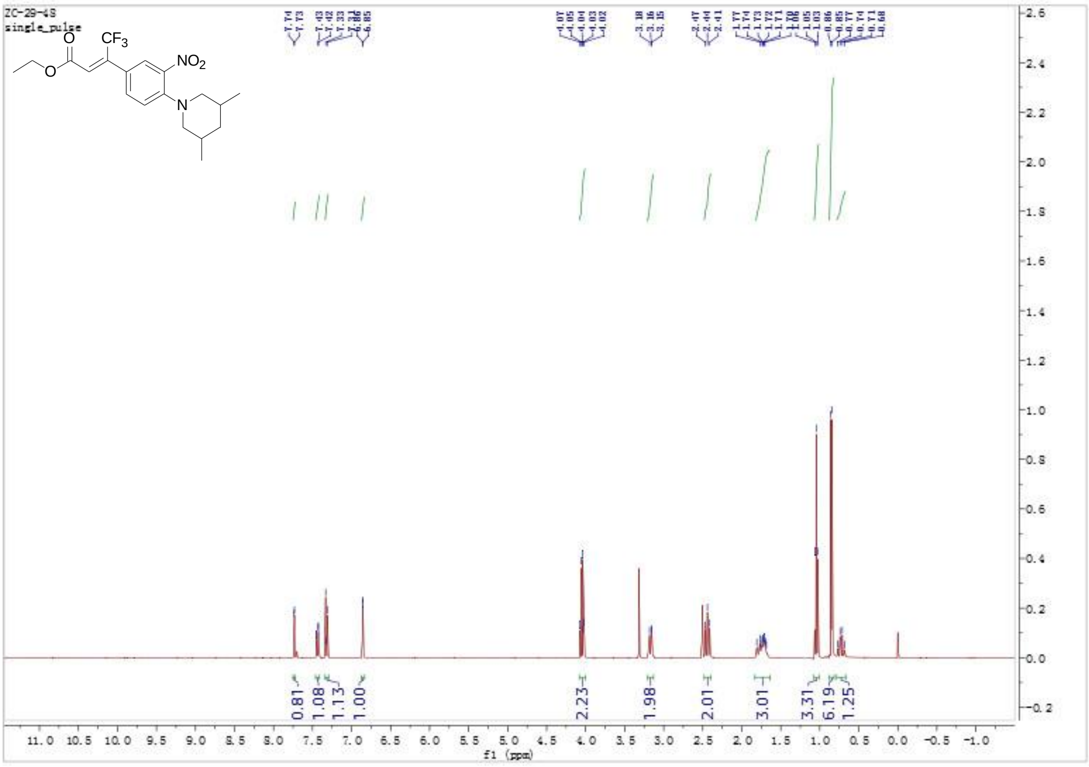

ESI-MS spectra of compound e

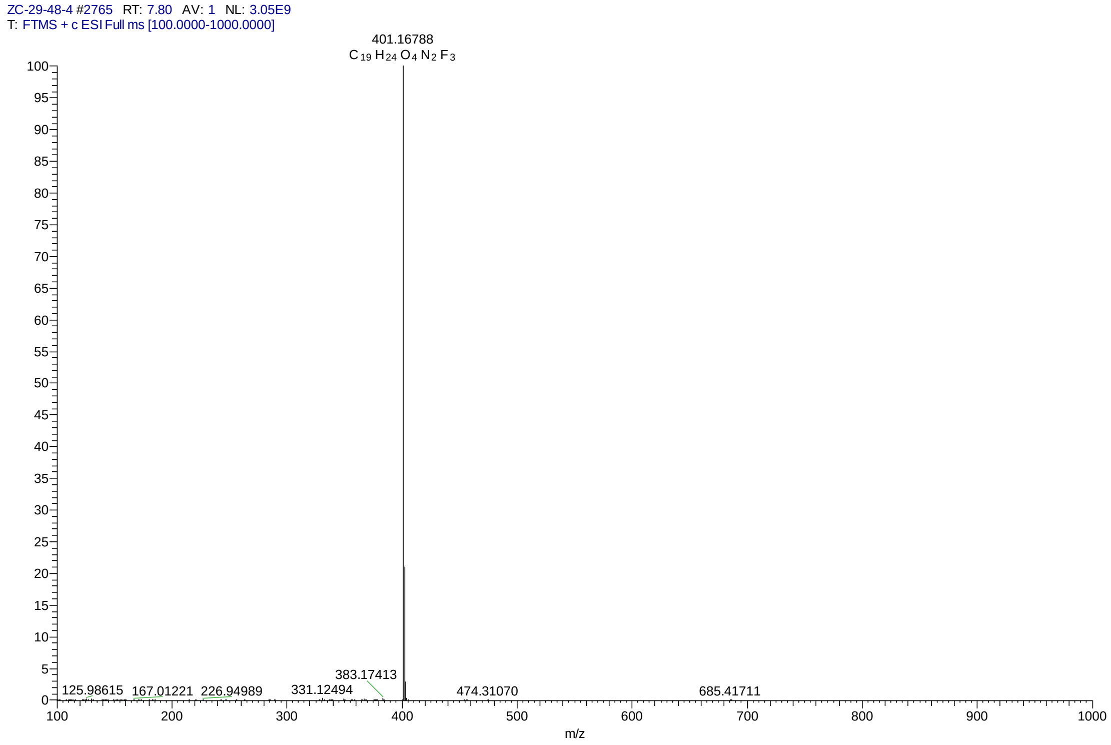

m/z	Theo. Mass	Delta (ppm)	RDB equiv.	Composition
401.16788	401.16827	-0.97	7.5	C19 H24 O4 N2 F3

^{1}H NMR spectra of compound f

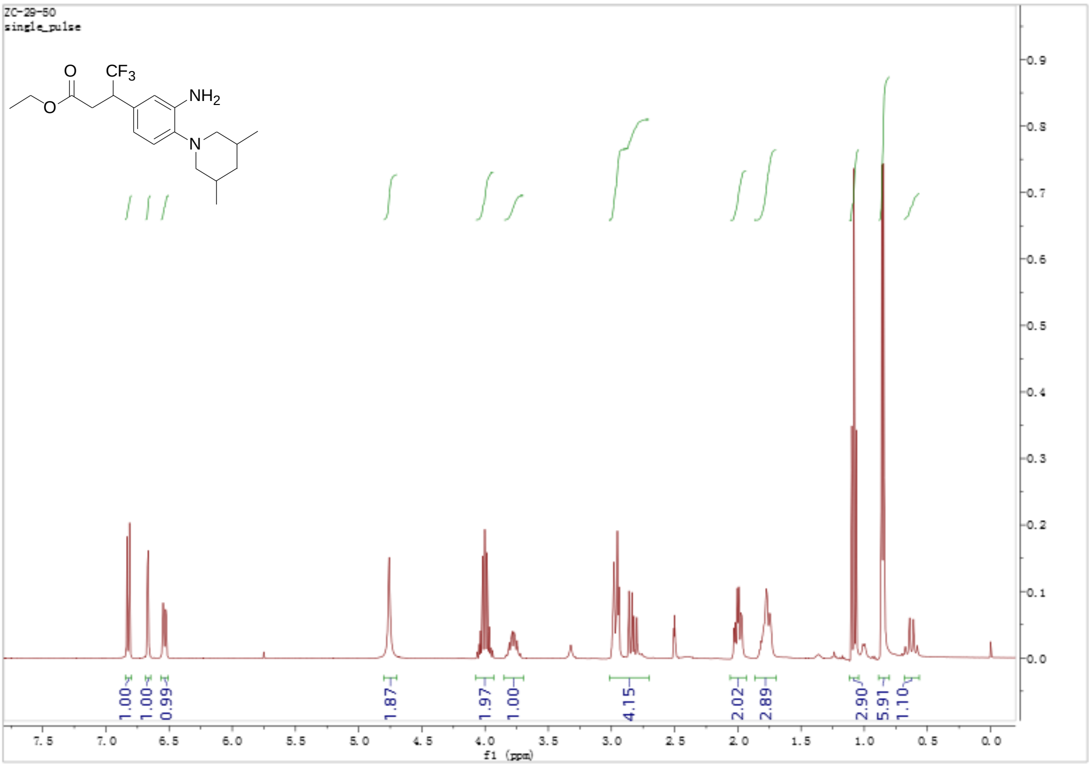

ESI-MS spectra of compound f

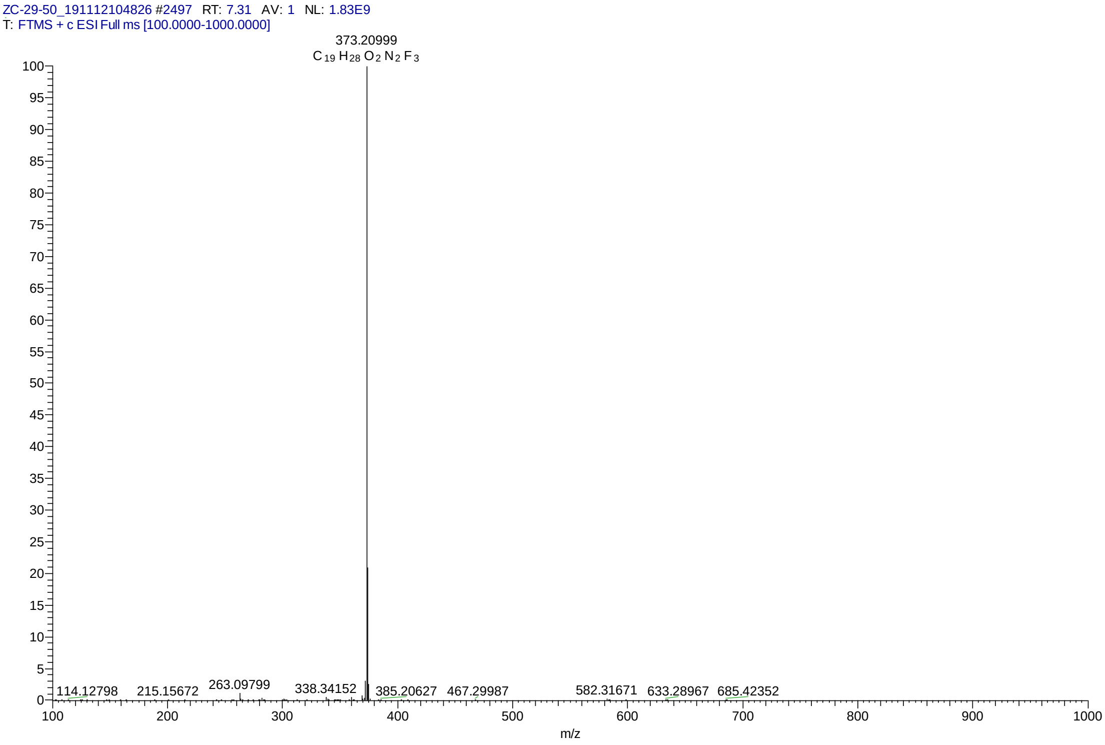

m/z	Theo. Mass	Delta (ppm)	RDB equiv.	Composition
373.20999	373.20974	0.67	5.5	C19 H28 O2 N2 F3

^{1}H NMR spectra of compound g1

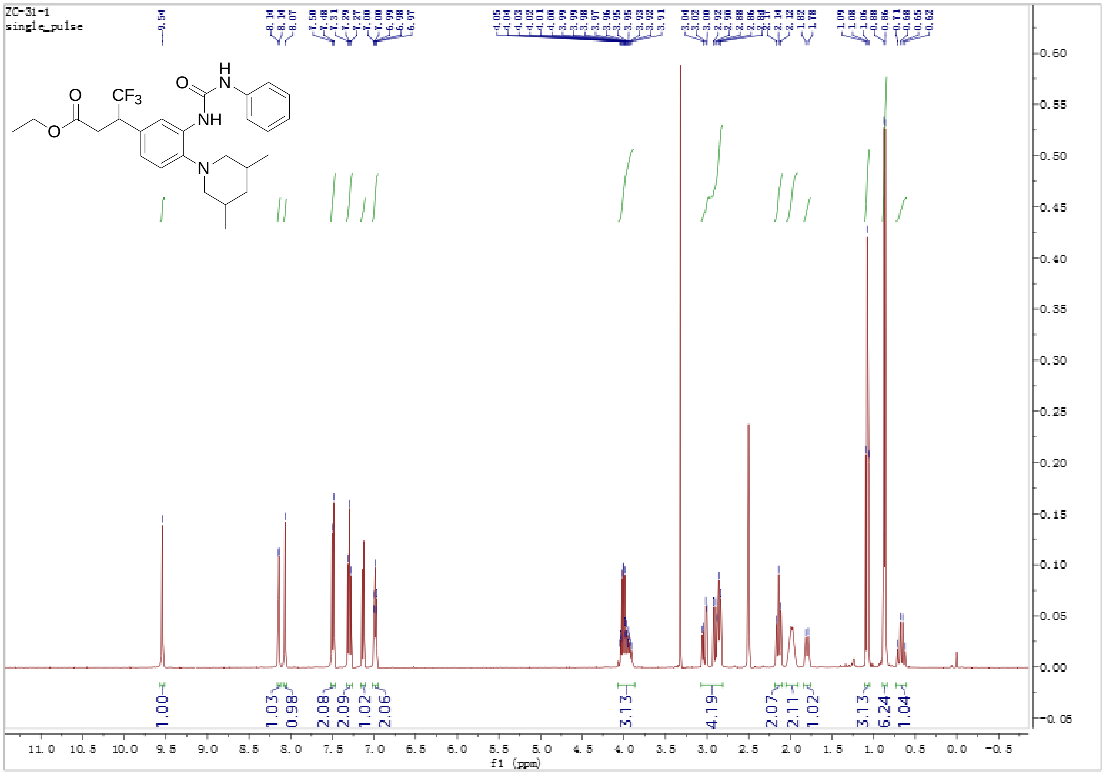

^{13}C NMR spectra of compound g1

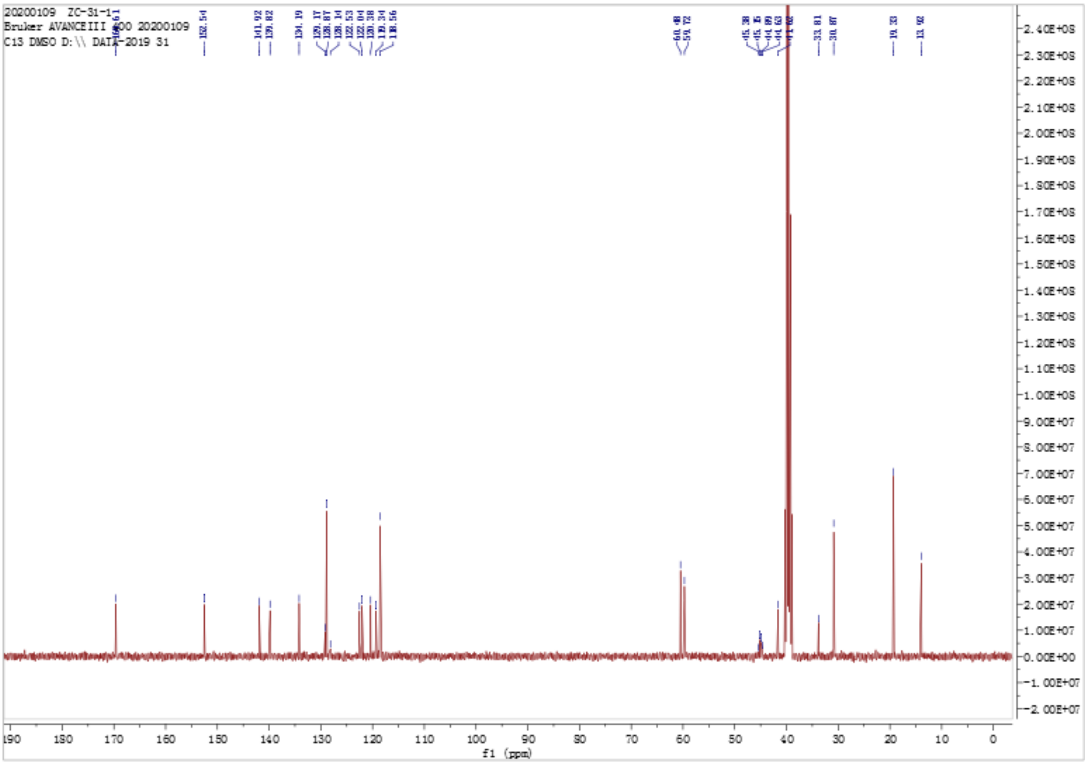

ESI-MS spectra of compound g1

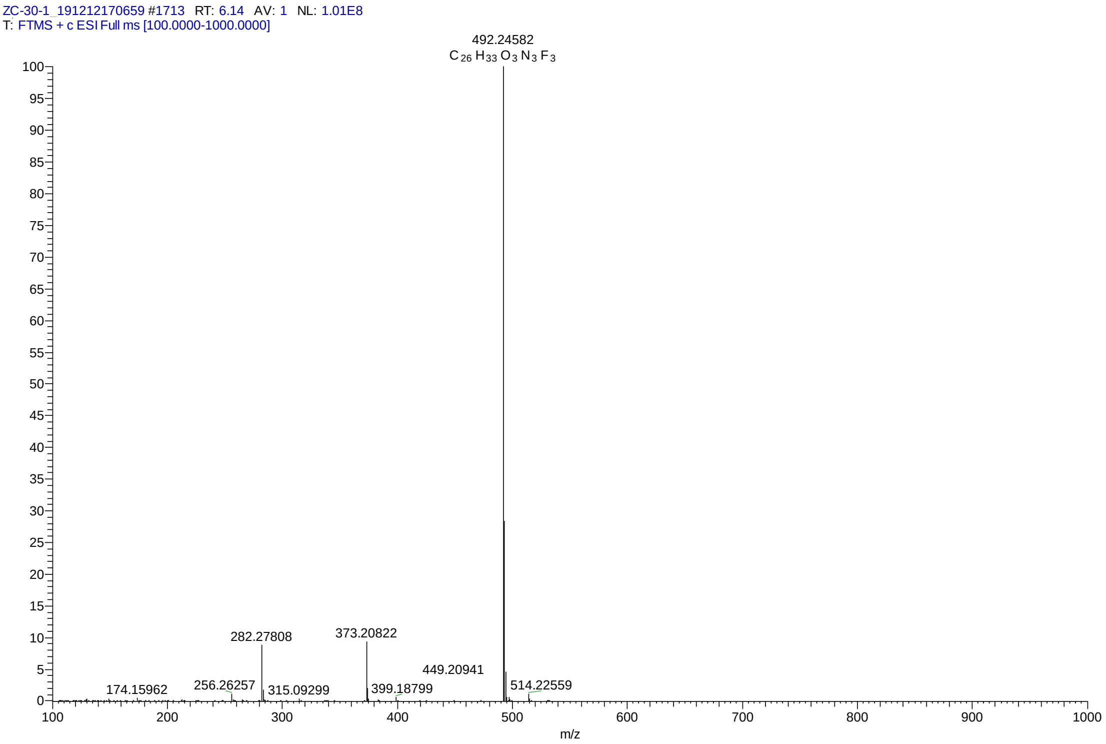

m/z	Theo. Mass	Delta (ppm)	RDB equiv.	Composition
492.24582	492.24685	-2.10	10.5	C26 H33 O3 N3 F3

^{1}H NMR spectra of compound i1

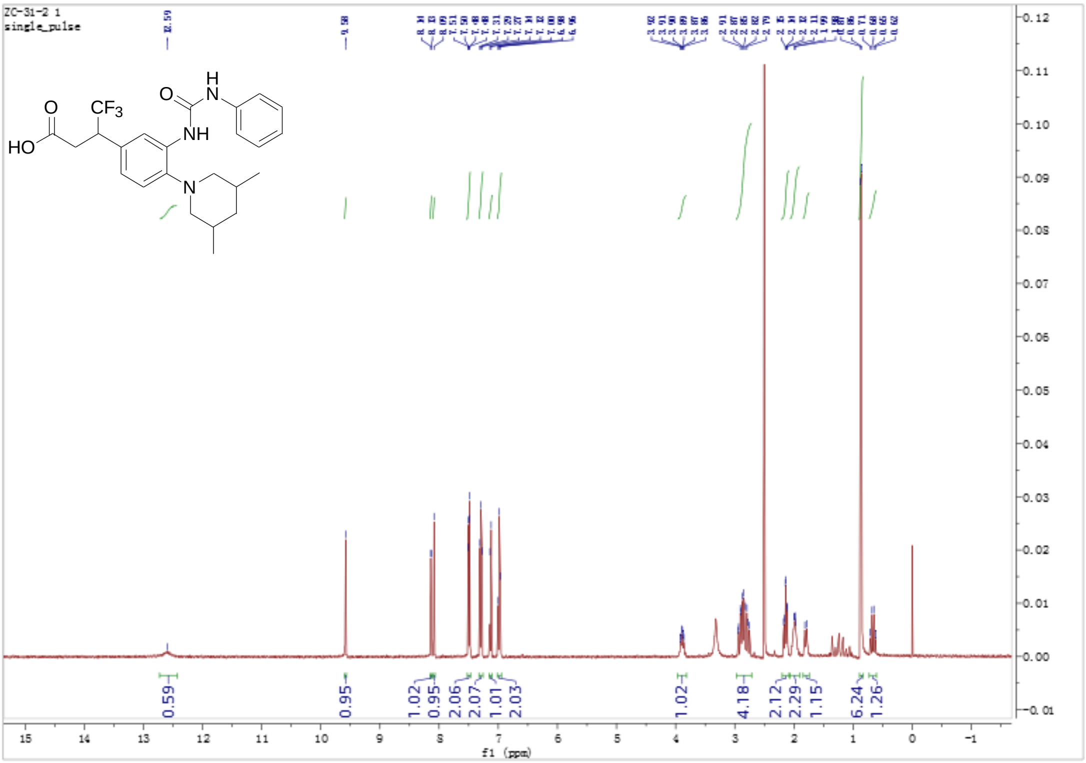

ESI-MS spectra of compound i1

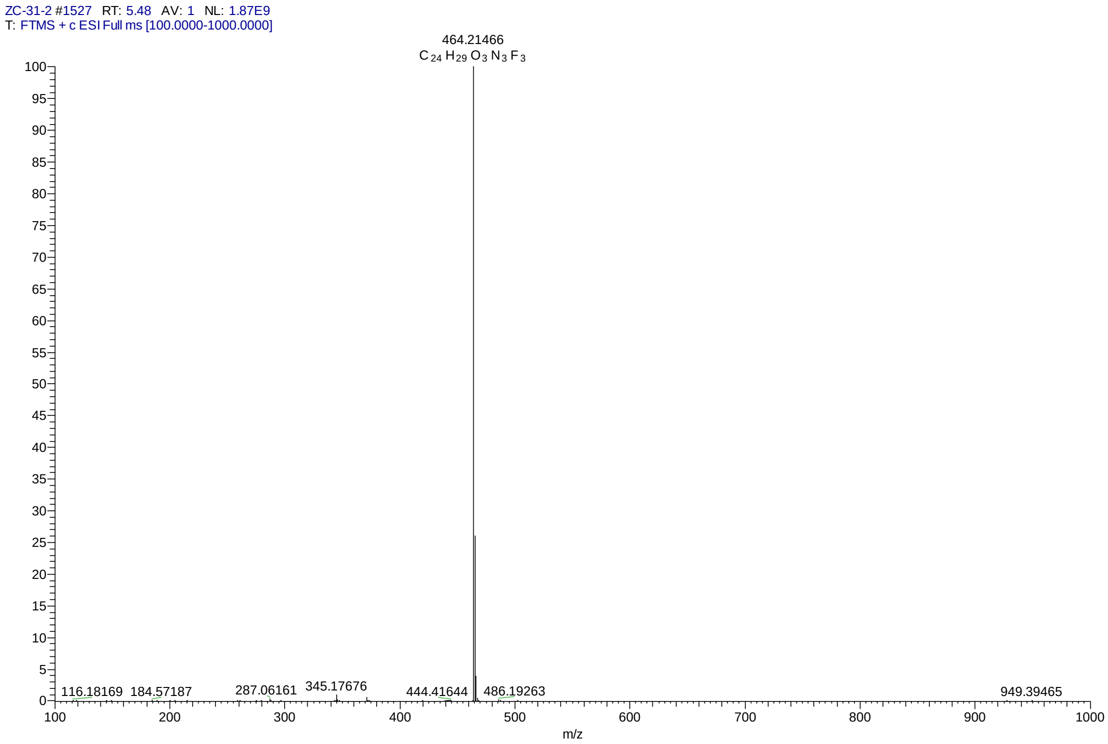

m/z	Theo. Mass	Delta (ppm)	RDB equiv.	Composition
464.21466	464.21555	-1.92	10.5	C24 H29 O3 N3 F3

^{1}H NMR spectra of compound g2

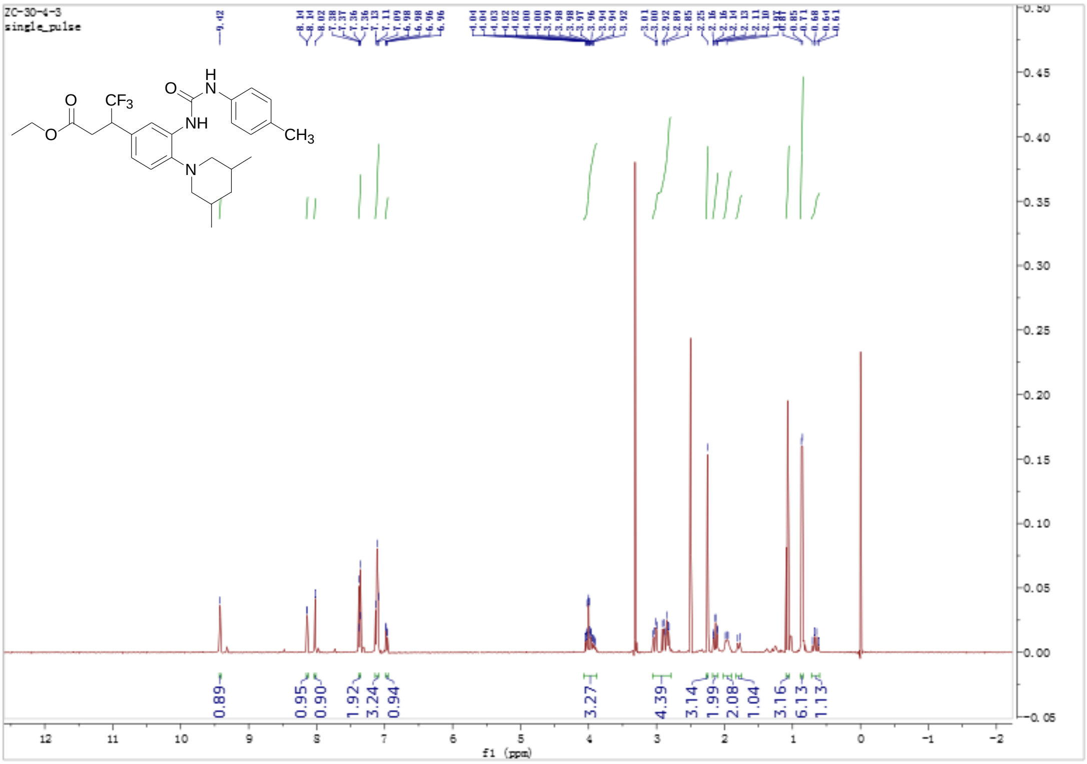

^{13}C NMR spectra of compound g2

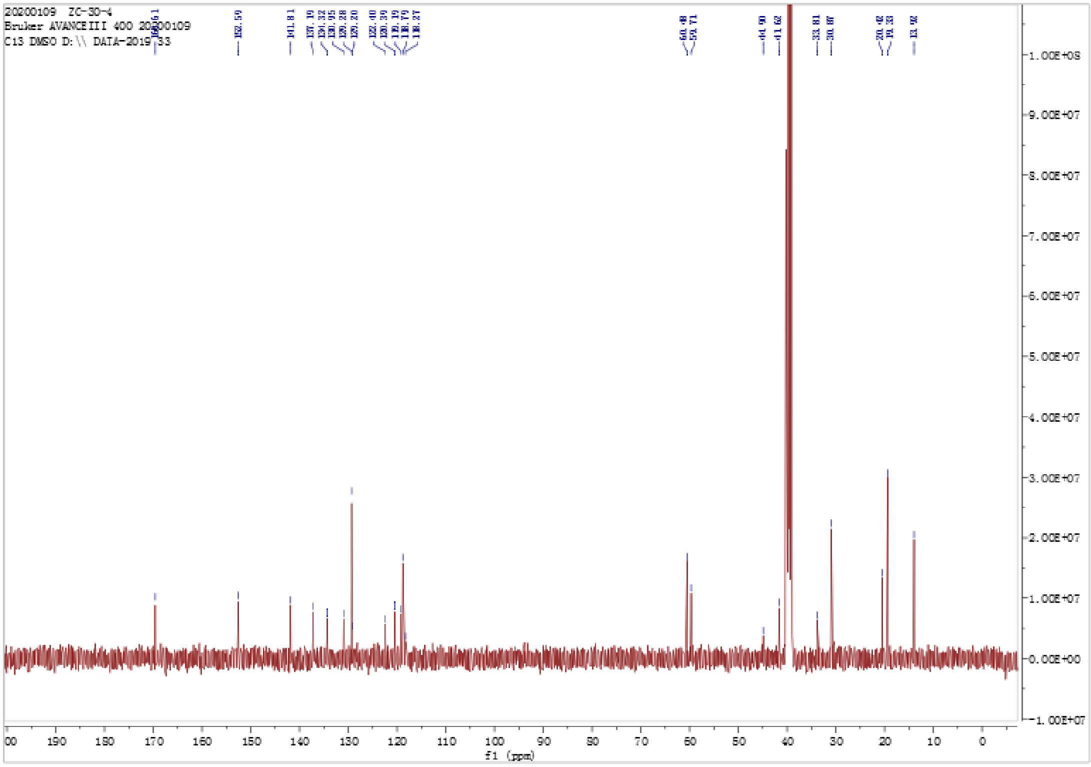

ESI-MS spectra of compound g2

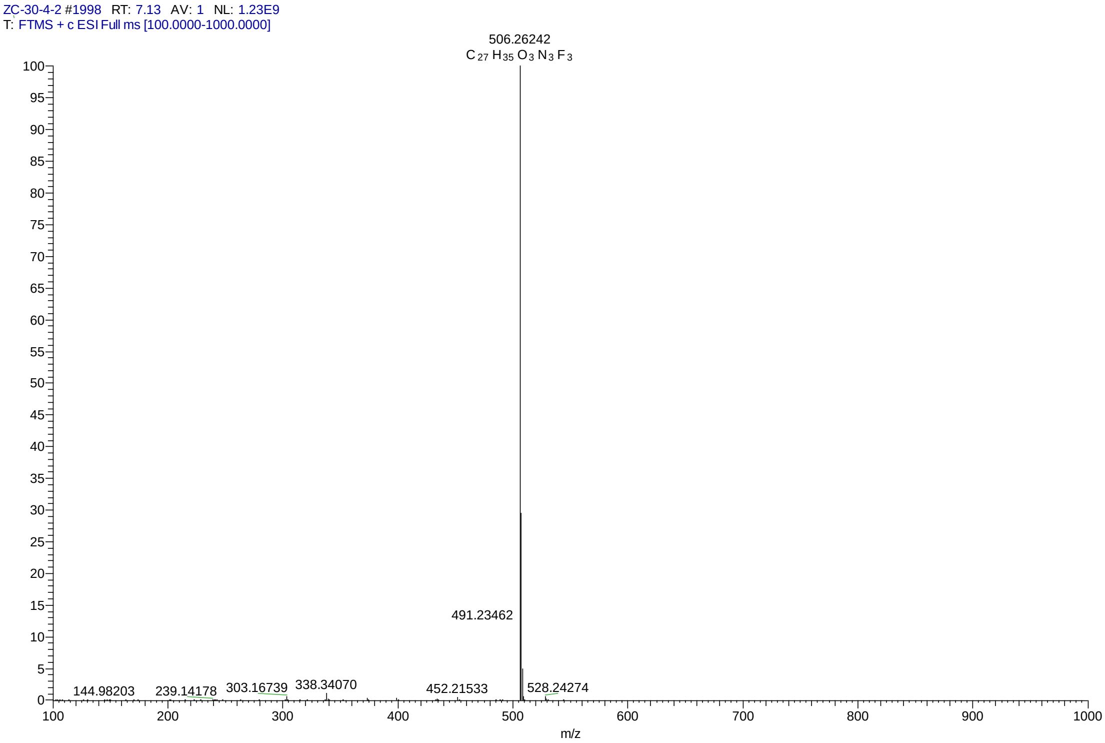

m/z	Theo. Mass	Delta (ppm)	RDB equiv.	Composition
506.26242	506.26250	-0.16	10.5	C27 H35 O3 N3 F3

^{1}H NMR spectra of compound i2

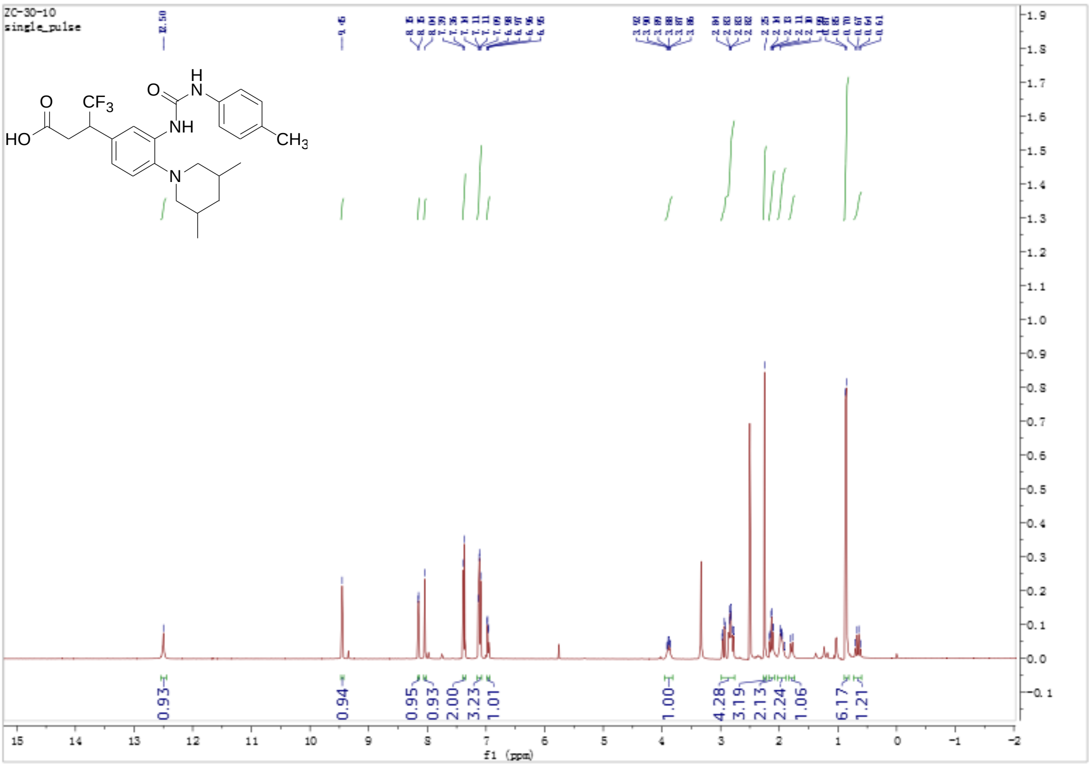

^{13}C NMR spectra of compound i2

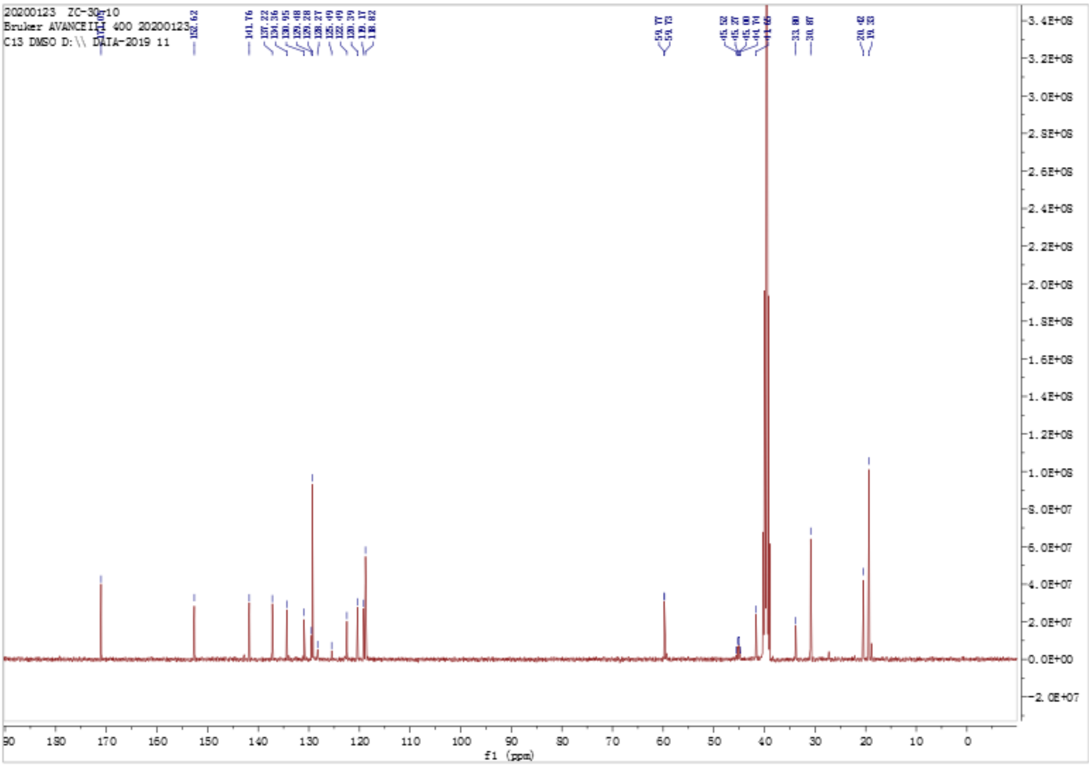

ESI-MS spectra of compound i2

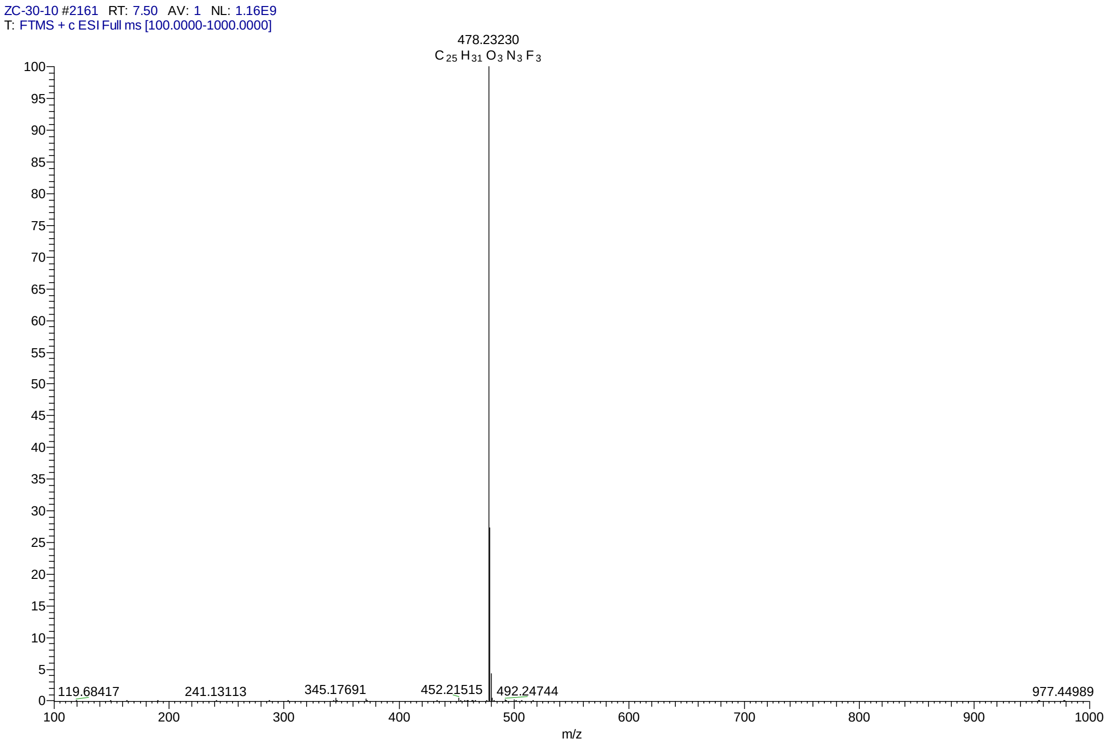

m/z	Theo. Mass	Delta (ppm)	RDB equiv.	Composition
478.23230	478.23120	2.29	10.5	C25 H31 O3 N3

^{1}H NMR spectra of compound g4

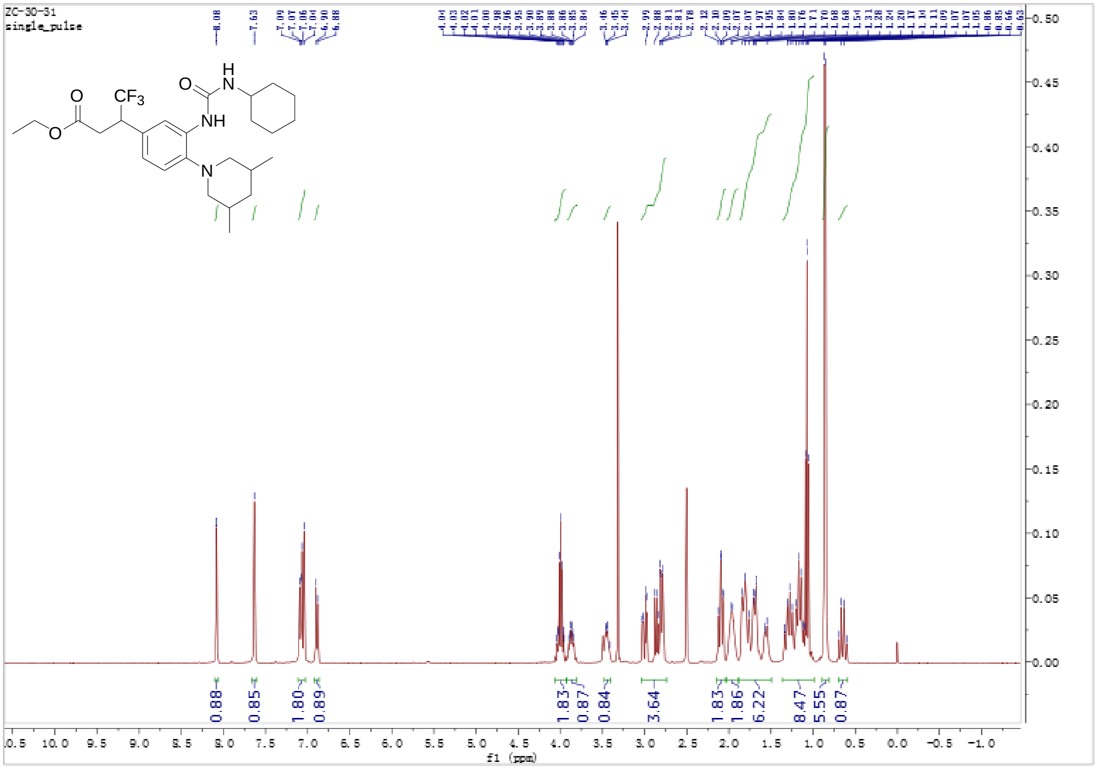

ESI-MS spectra of compound g4

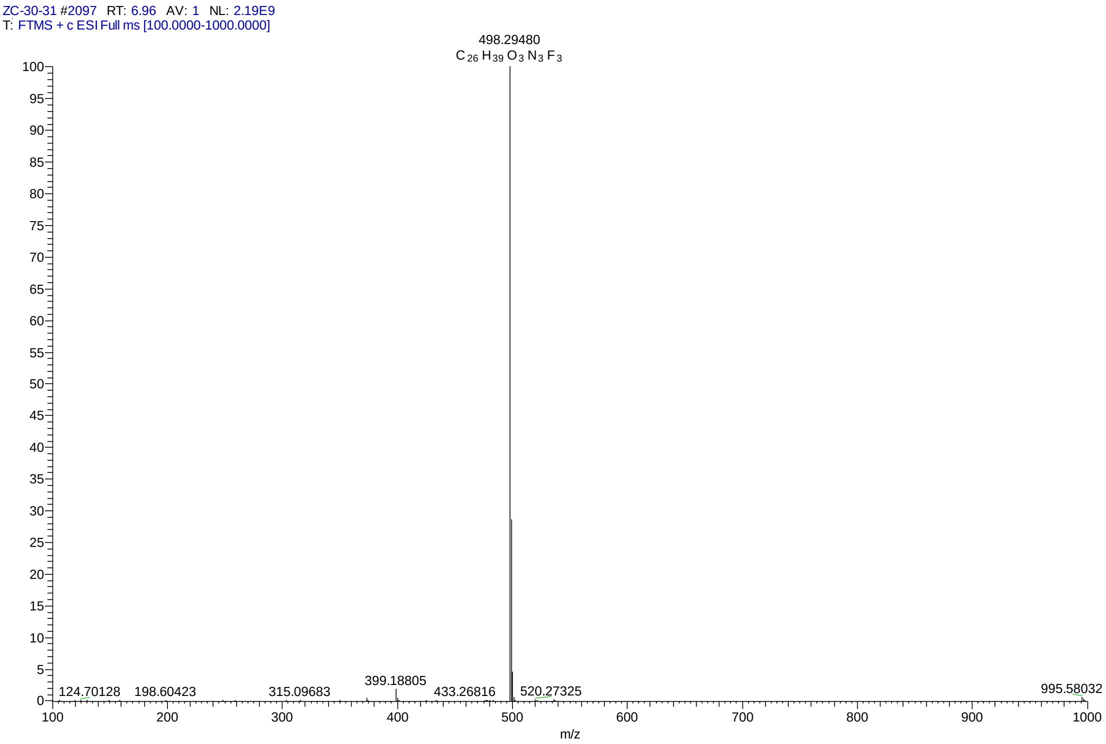

m/z	Theo. Mass	Delta (ppm)	RDB equiv.	Composition
498.29480	498.29380	2.00	7.5	C26 H39 O3 N3 F3

^{1}H NMR spectra of compound i4

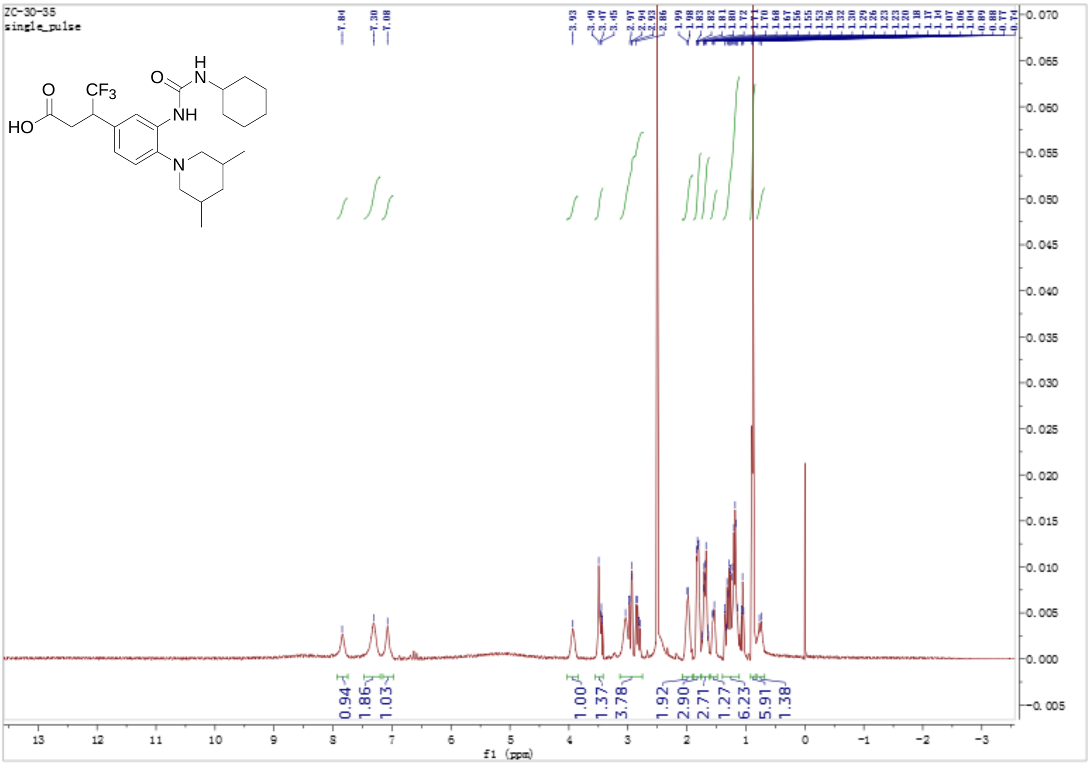

ESI-MS spectra of compound i4

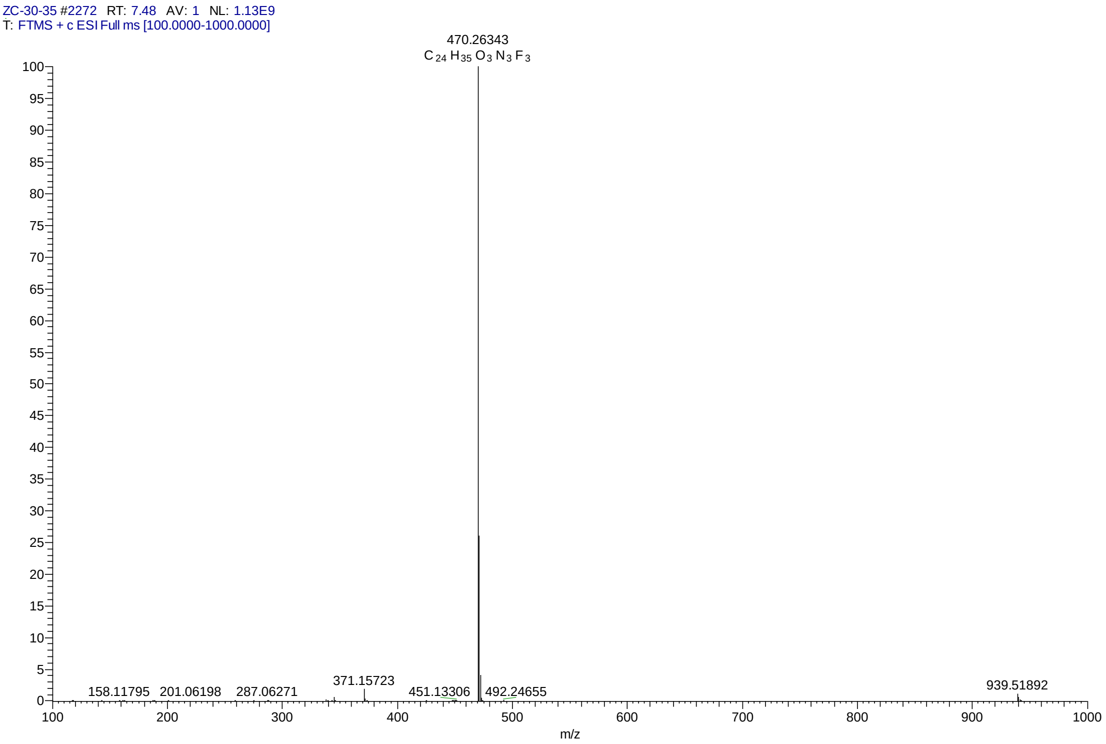

m/z	Theo. Mass	Delta (ppm)	RDB equiv.	Composition
470.26343	470.26250	1.97	7.5	C24 H35 O3 N3 F3

^{1}H NMR spectra of compound g5

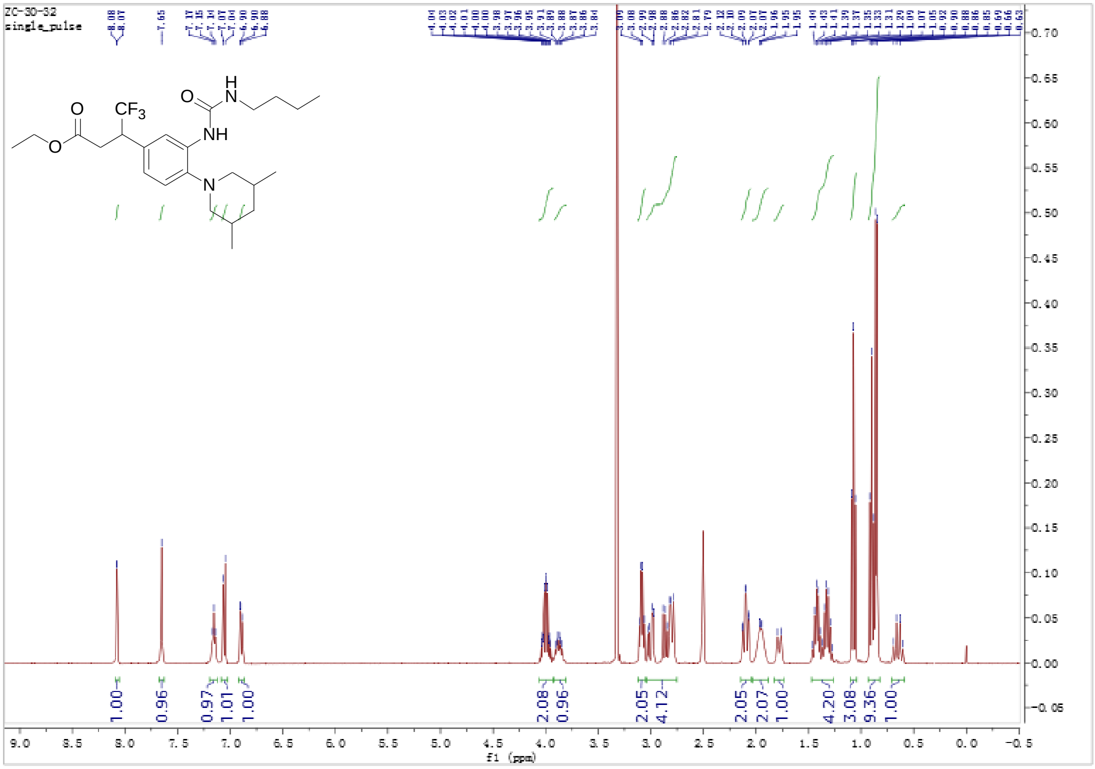

ESI-MS spectra of compound g5

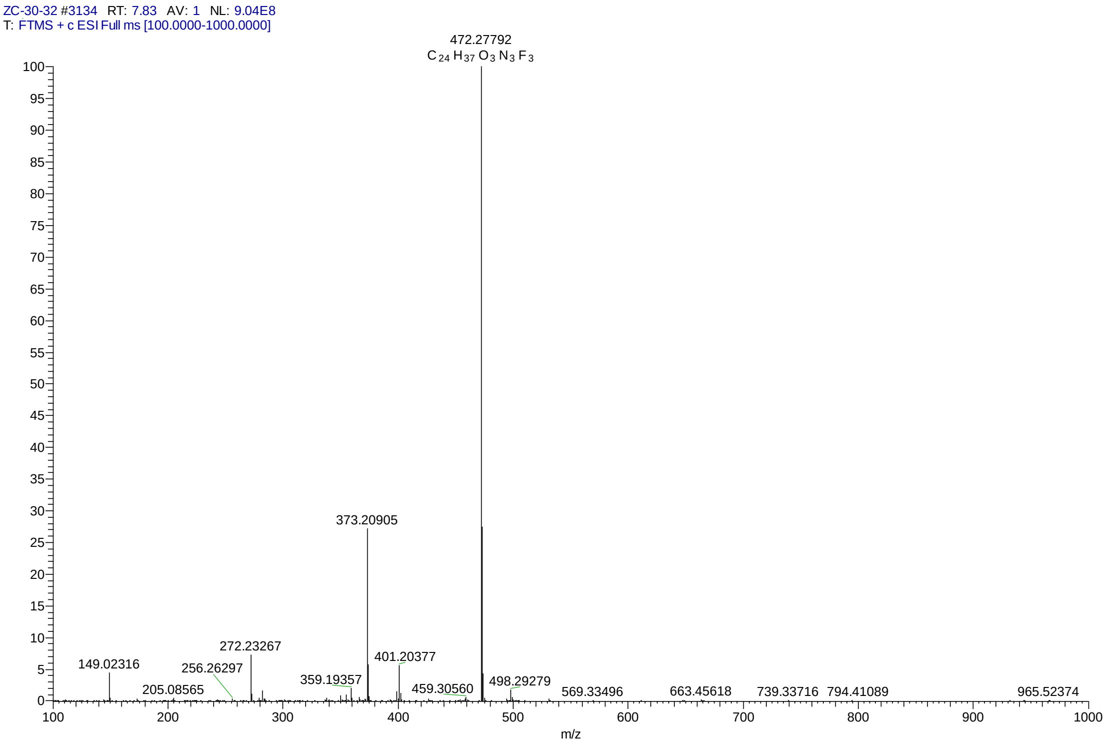

m/z	Theo. Mass	Delta (ppm)	RDB equiv.	Composition
472.27792	472.27815	-0.49	6.5	C24 H37 O3 N3 F3

^{1}H NMR spectra of compound i5

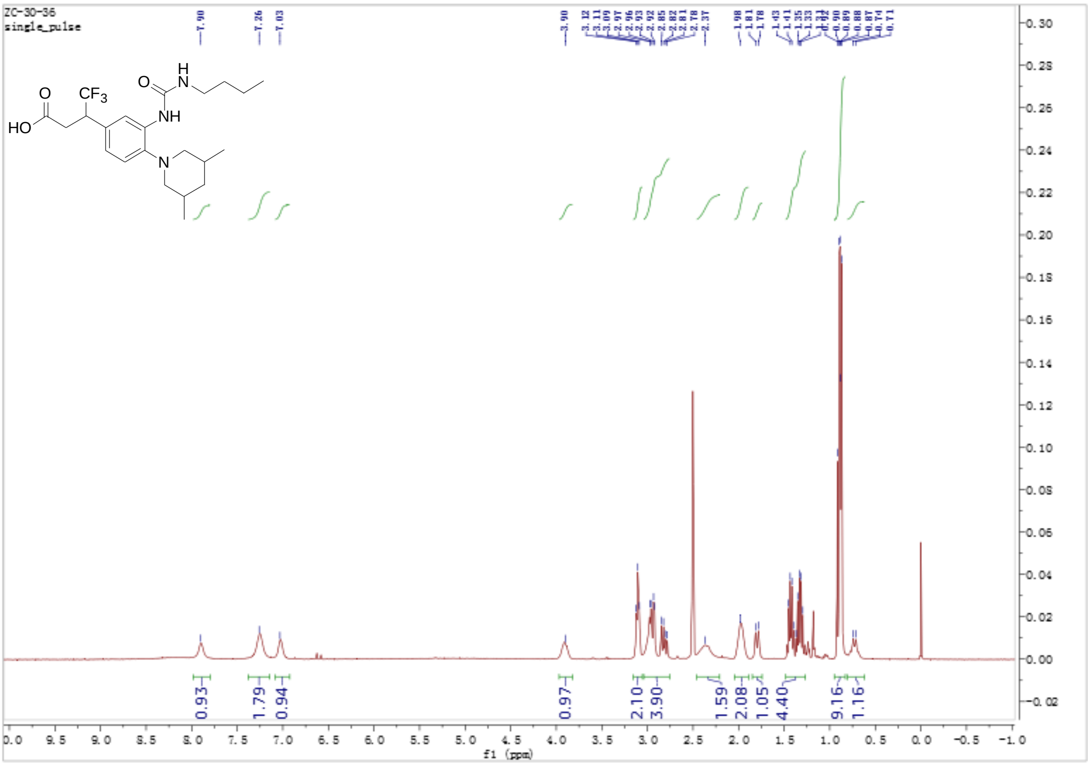

ESI-MS spectra of compound i5

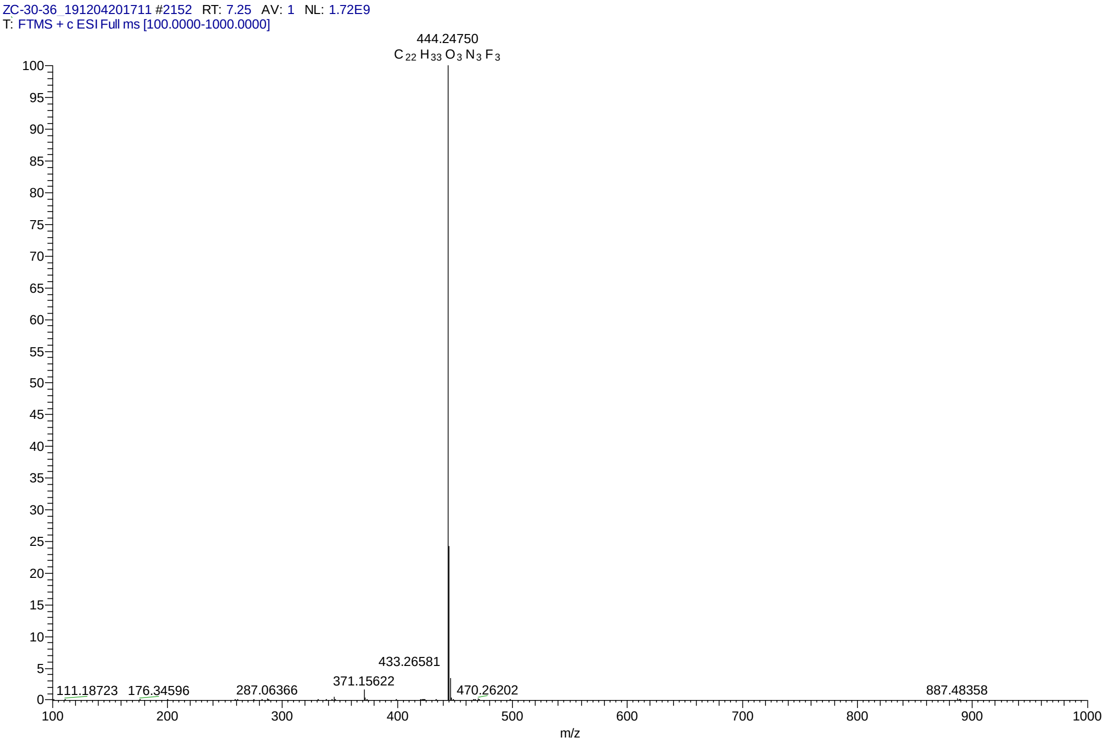

m/z	Theo. Mass	Delta (ppm)	RDB equiv.	Composition
444.24750	444.24685	1.46	6.5	C22 H33 O3 N3 F3

^{1}H NMR spectra of compound g8

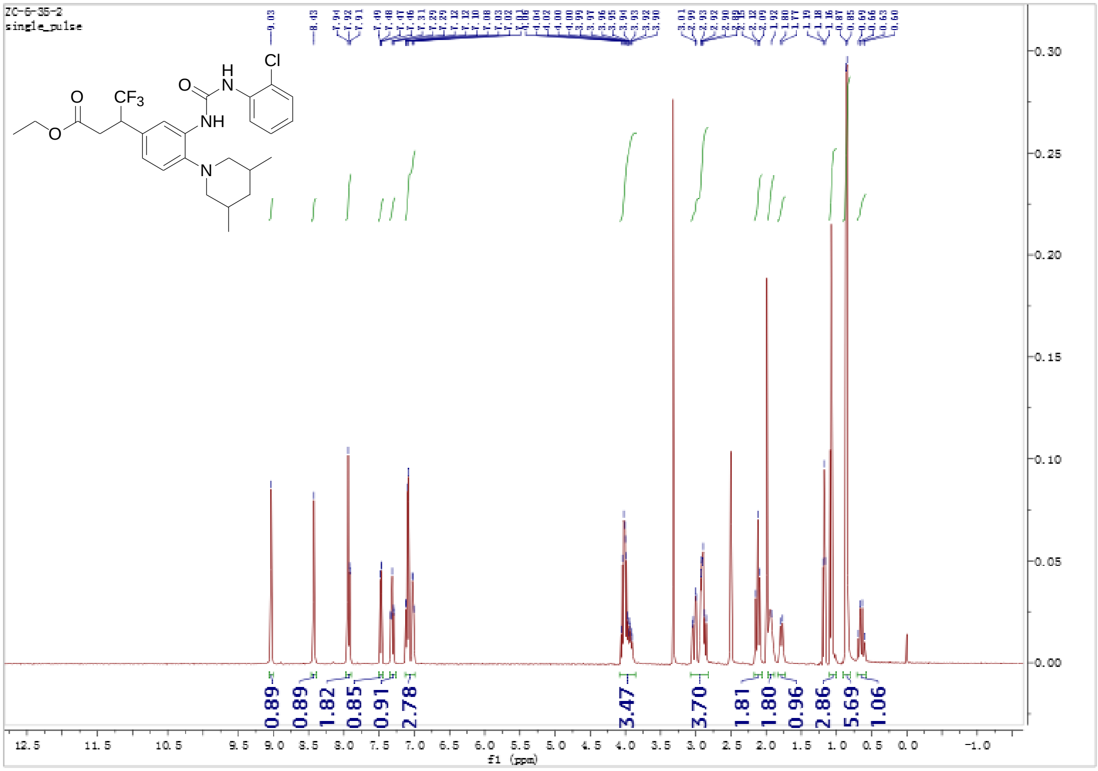

ESI-MS spectra of compound g8

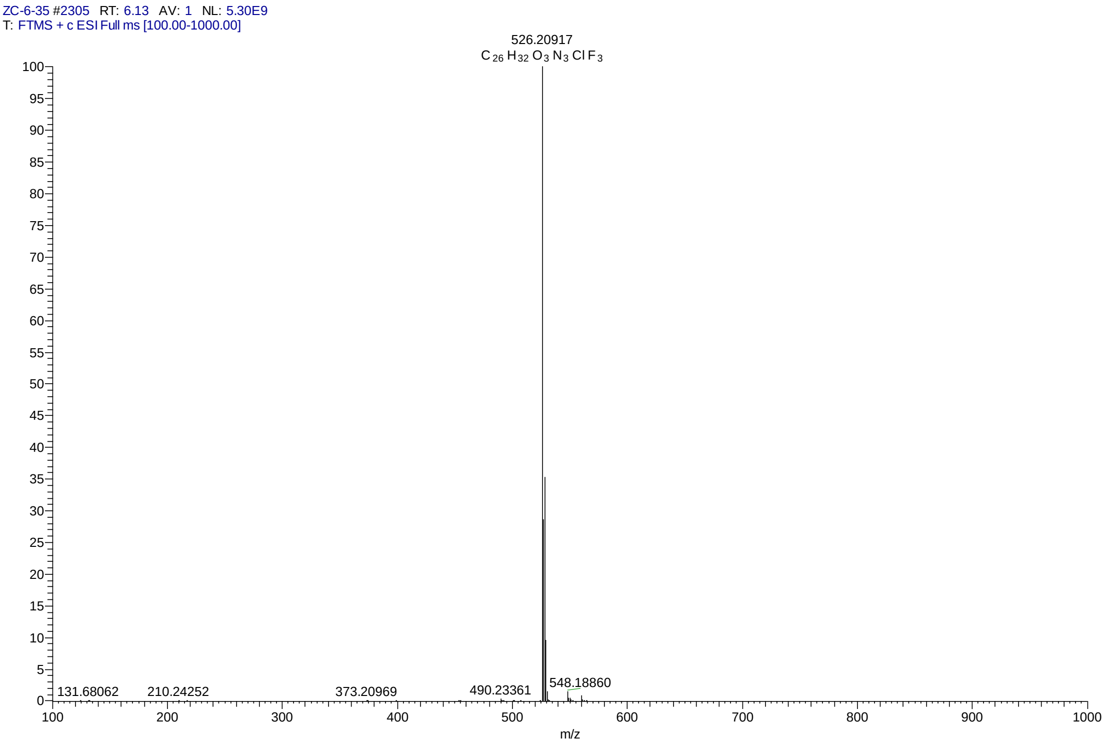

m/z	Theo. Mass	Delta (ppm)	RDB equiv.	Composition
526.20917	526.20788	2.45	10.5	C26 H32 O3 N3 Cl F3

^{1}H NMR spectra of compound i8

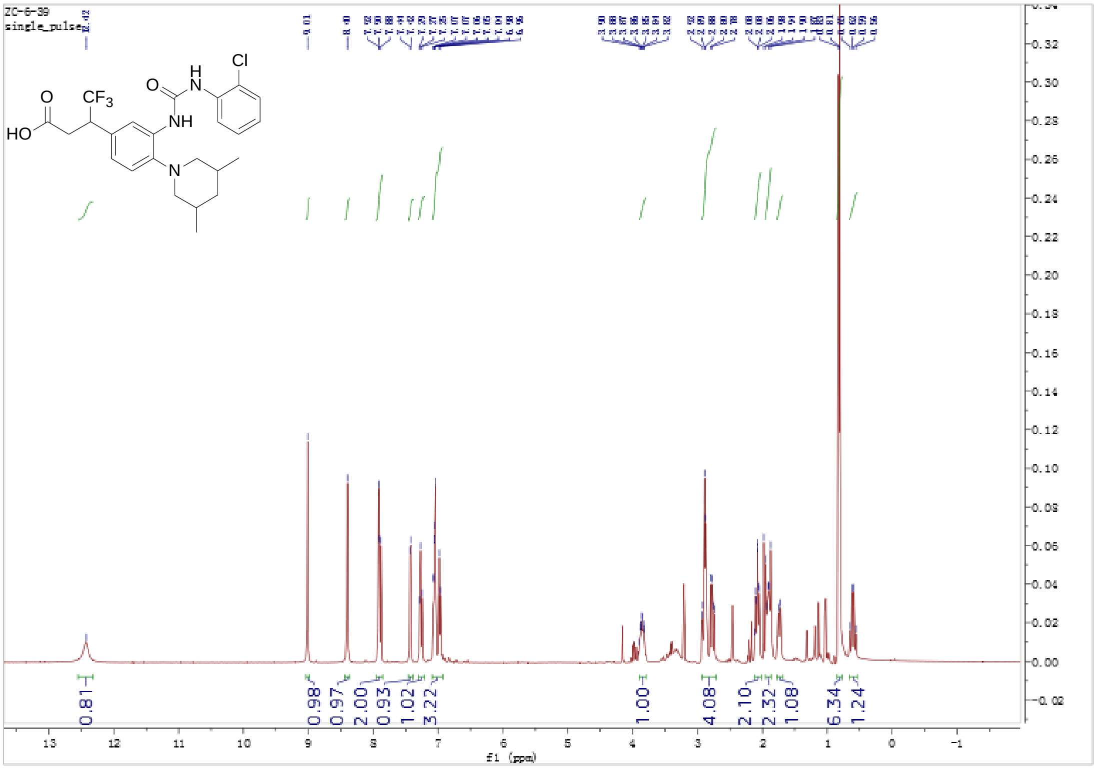

ESI-MS spectra of compound i8

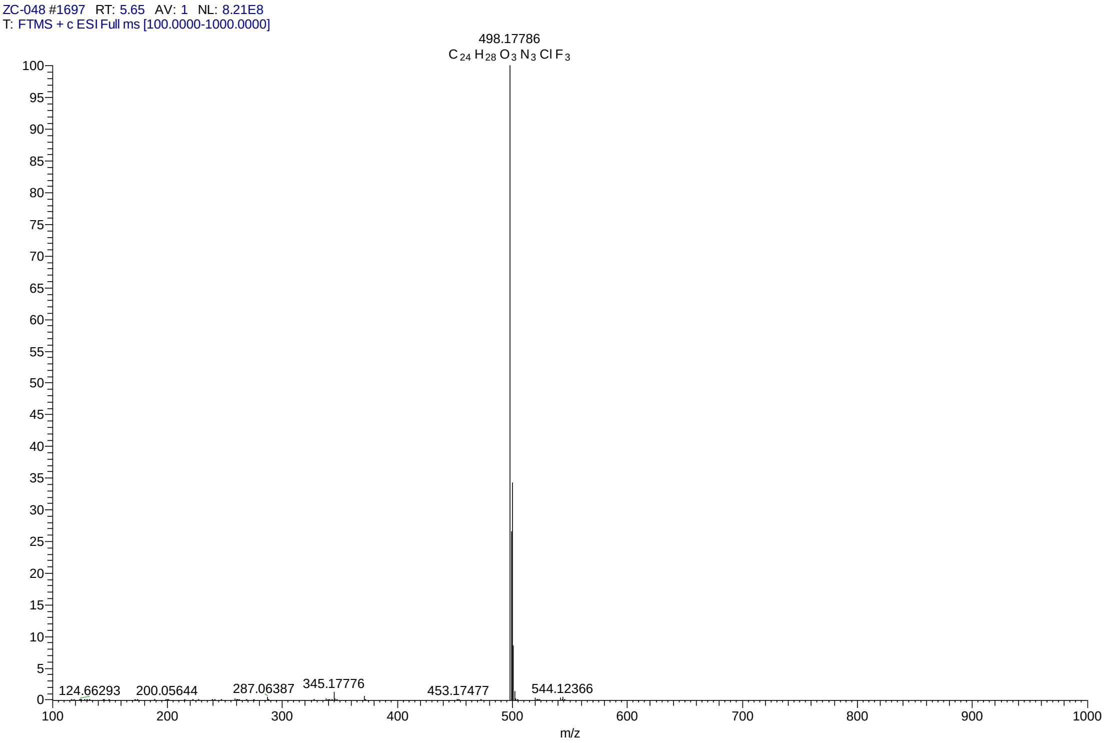

m/z	Theo. Mass	Delta (ppm)	RDB equiv.	Composition
498.17786	498.17658	2.57	10.5	C24 H28 O3 N3 Cl F3

^{1}H NMR spectra of compound i9

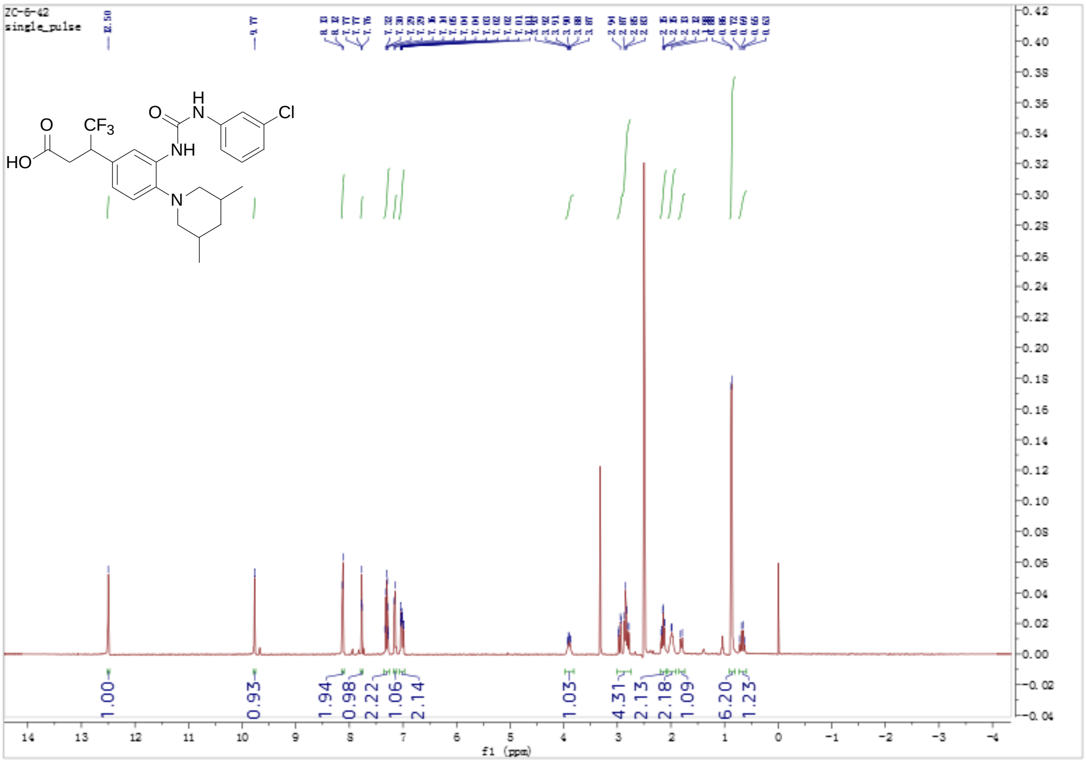

^{13}C NMR spectra of compound i9

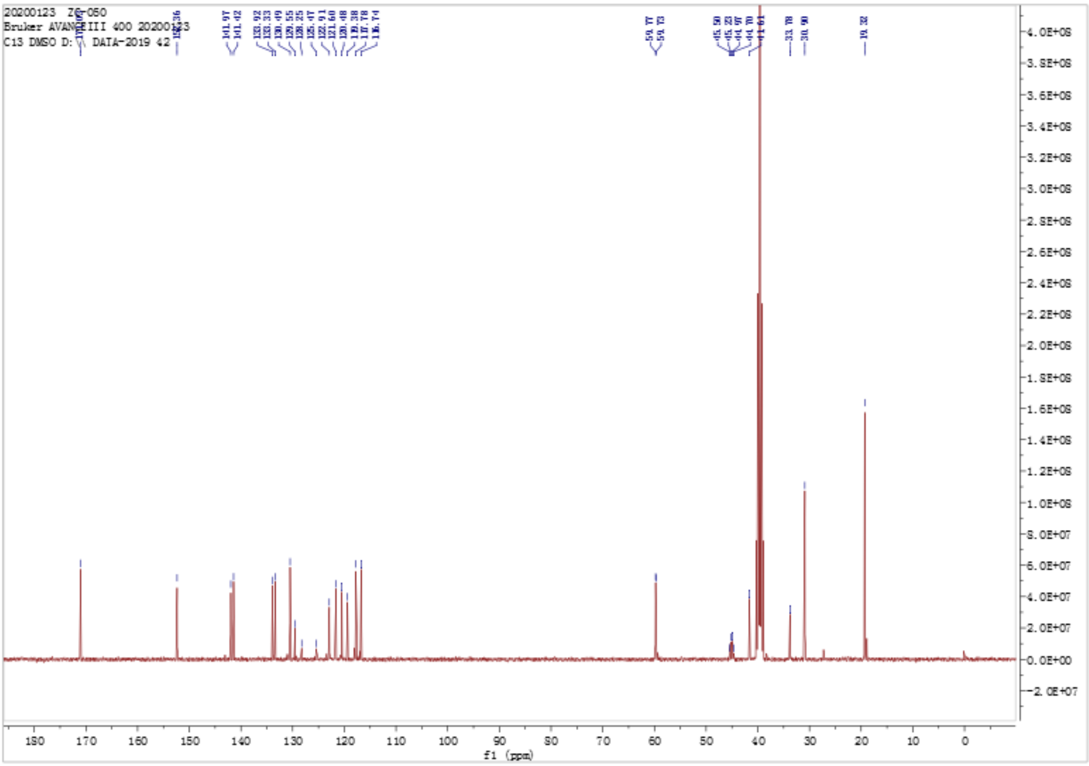

ESI-MS spectra of compound i9

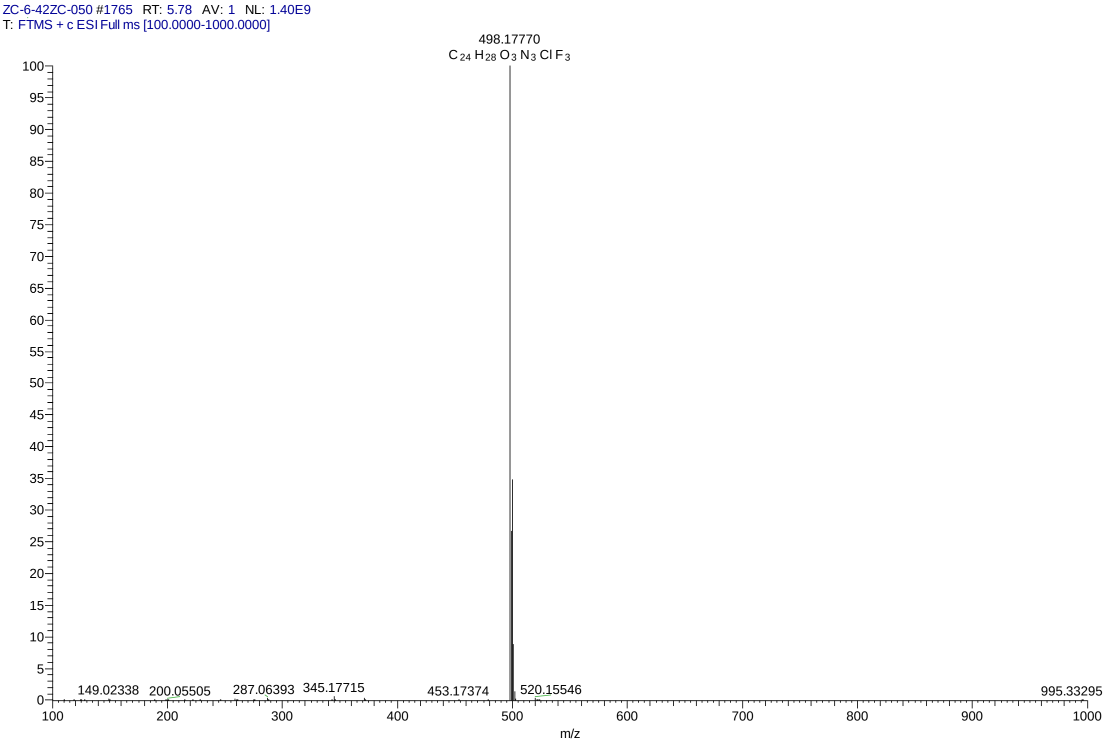

m/z	Theo. Mass	Delta (ppm)	RDB equiv.	Composition
498.17770	498.17658	2.25	10.5	C24 H28 O3 N3 Cl F3

^{1}H NMR spectra of compound g12

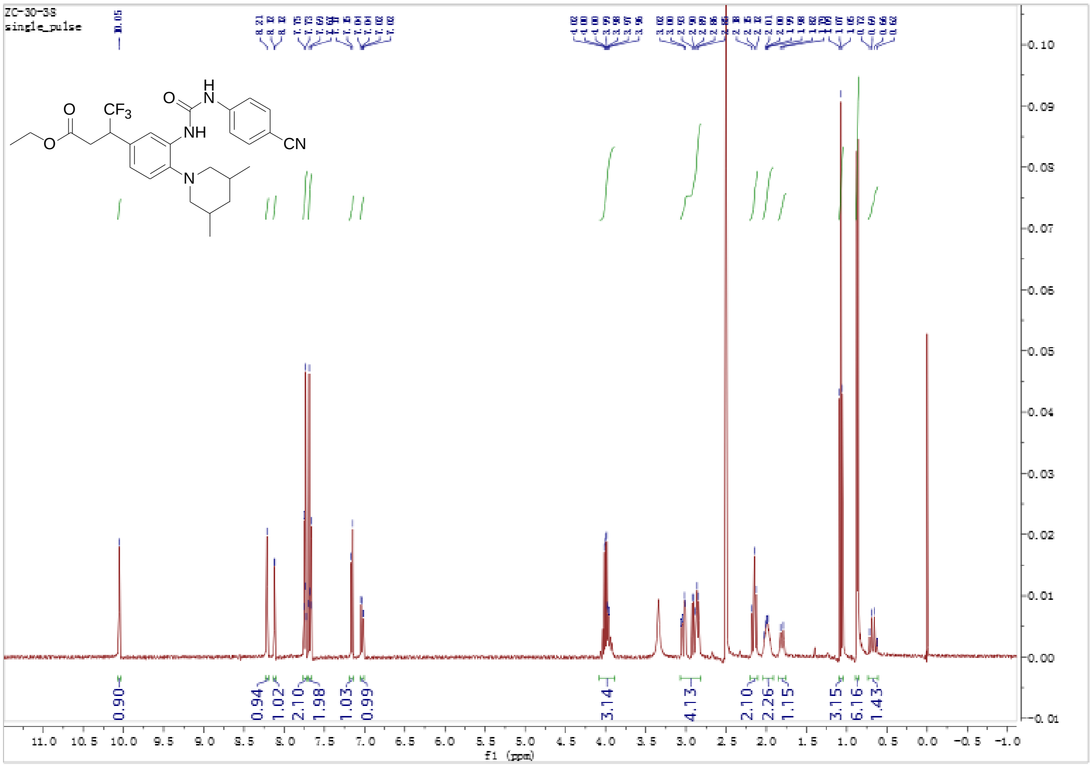

ESI-MS spectra of compound g12

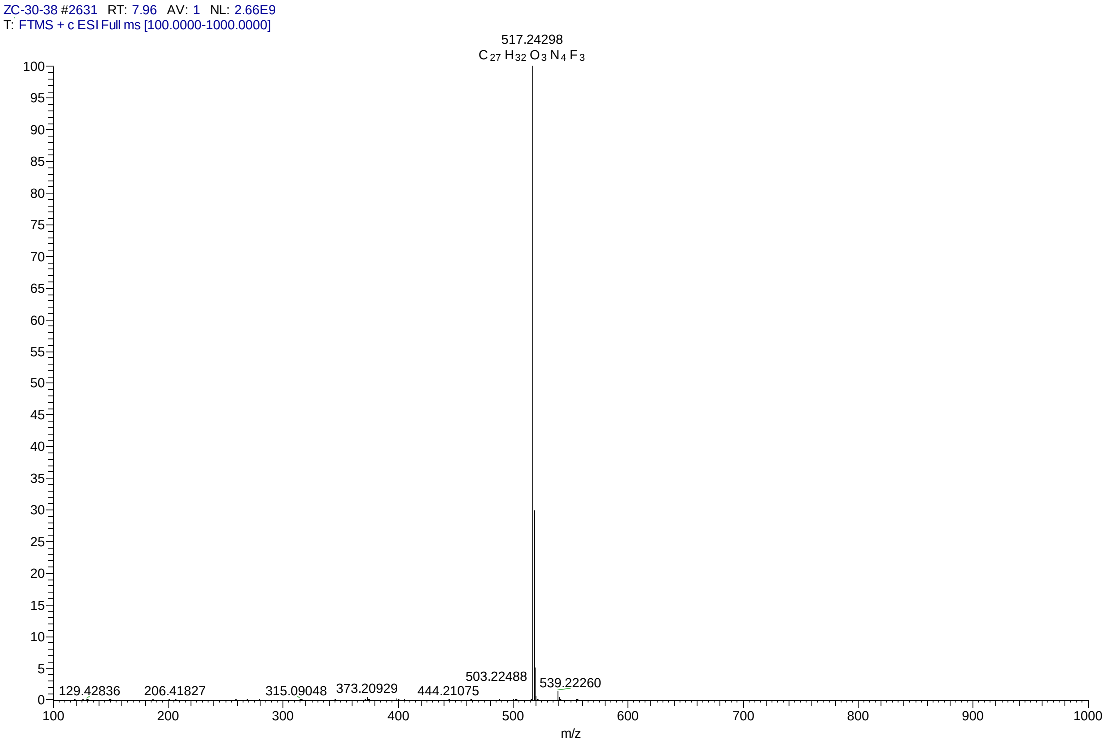

m/z	Theo. Mass	Delta (ppm)	RDB equiv.	Composition
517.24298	517.24210	1.70	12.5	C27 H32 O3 N4 F3

^{1}H NMR spectra of compound i12

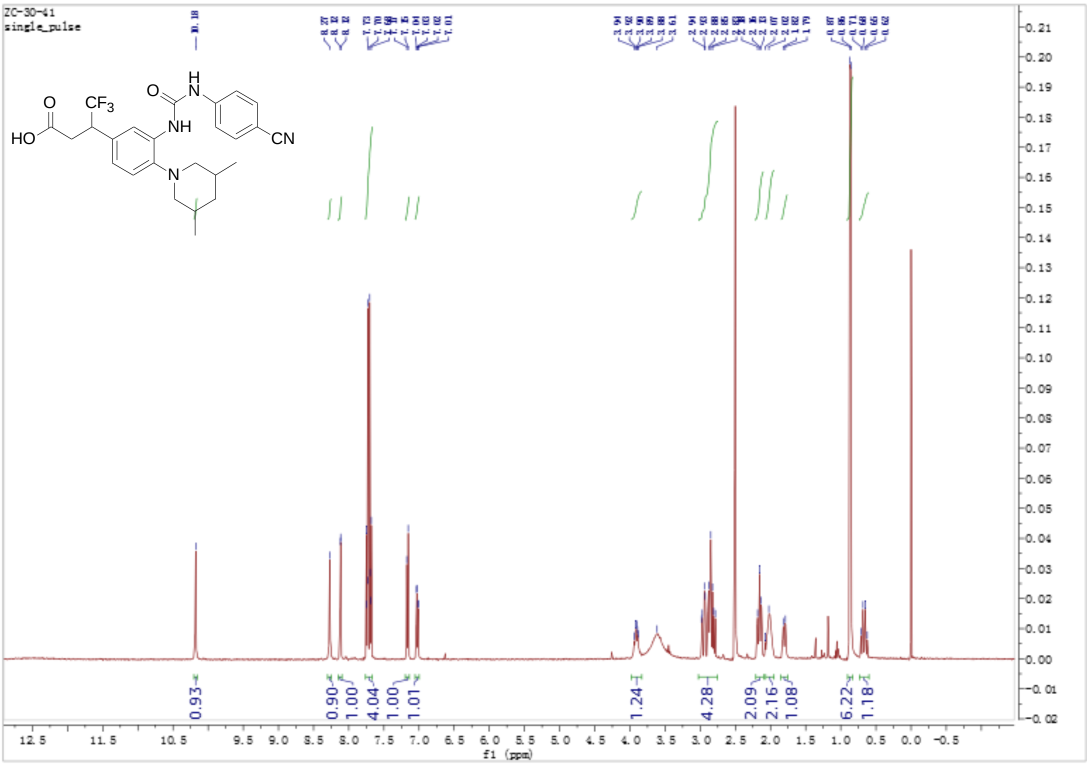

^{13}C NMR spectra of compound i12

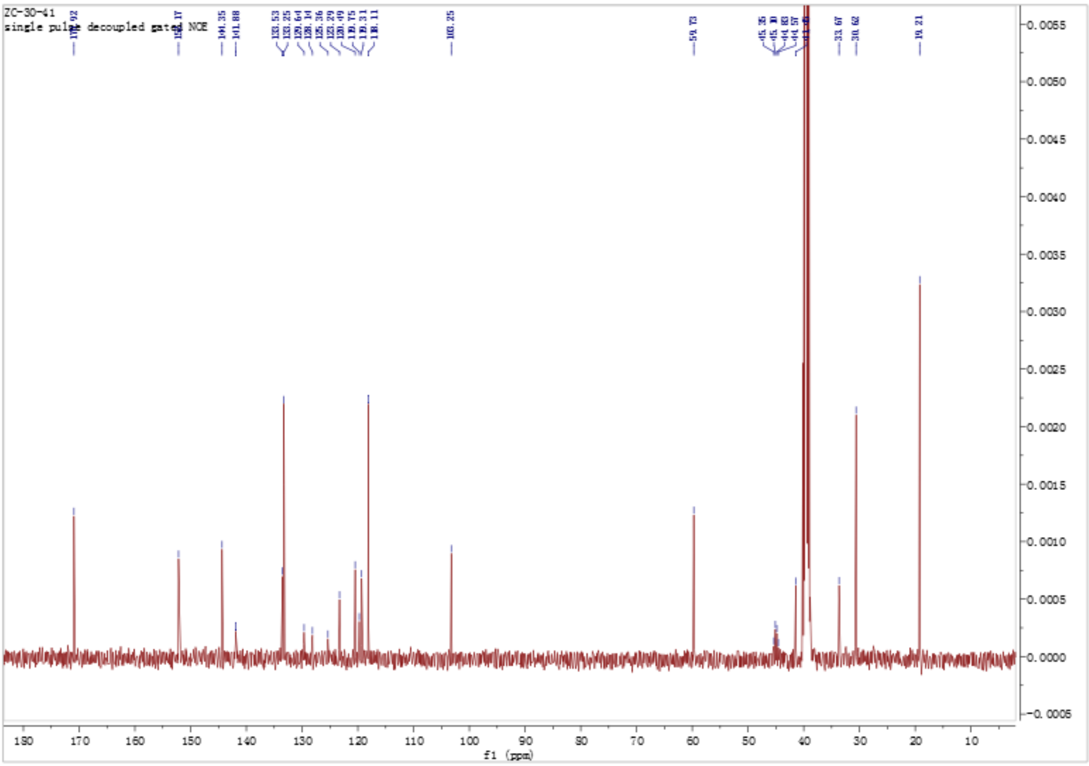

ESI-MS spectra of compound i12

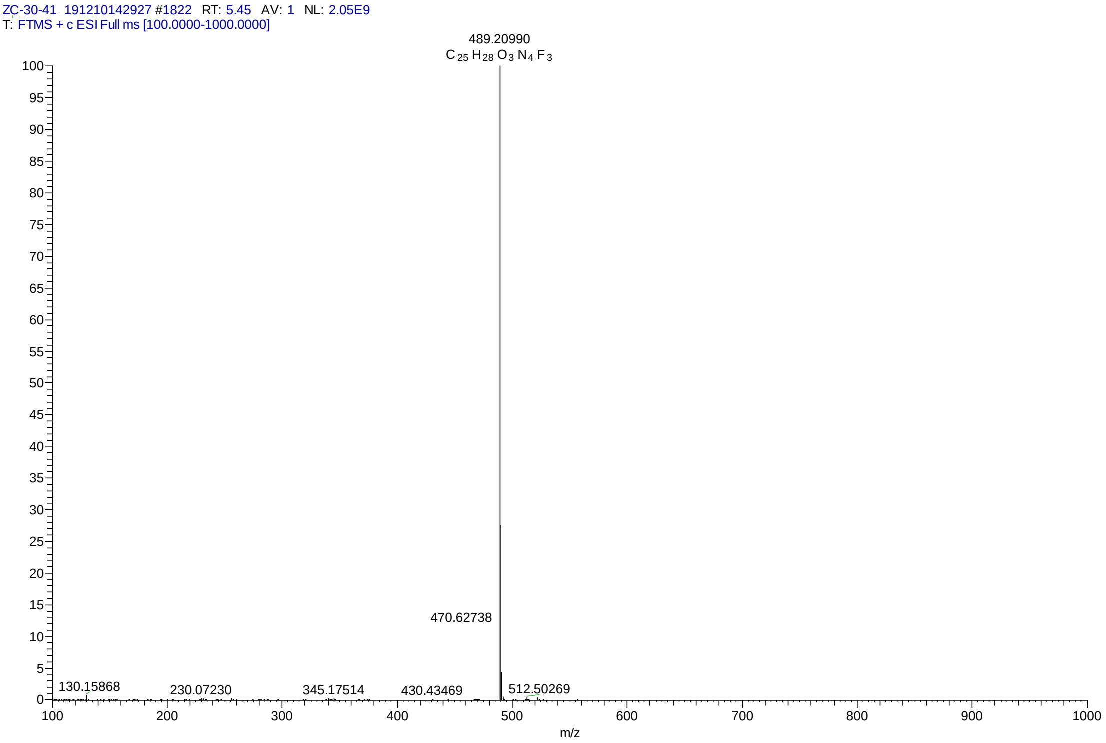

m/z	Theo. Mass	Delta (ppm)	RDB equiv.	Composition
489.20990	489.21080	-1.84	12.5	C25 H28 O3 N4 F3

^{1}H NMR spectra of compound g13

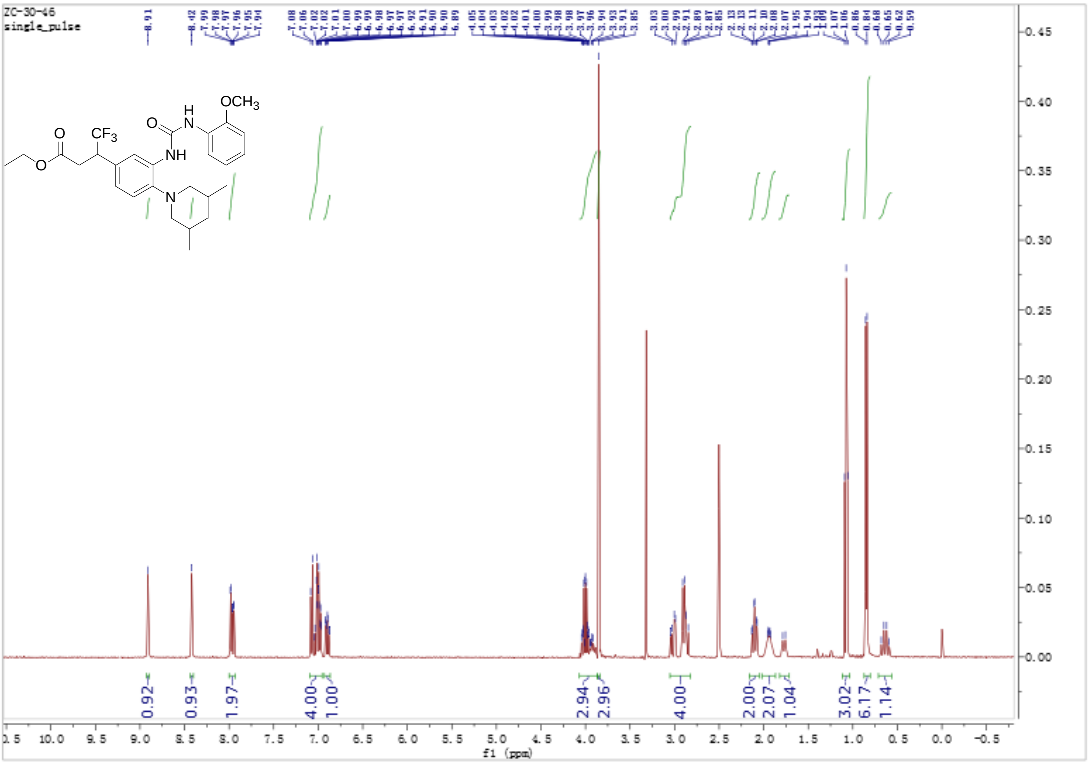

ESI-MS spectra of compound g13

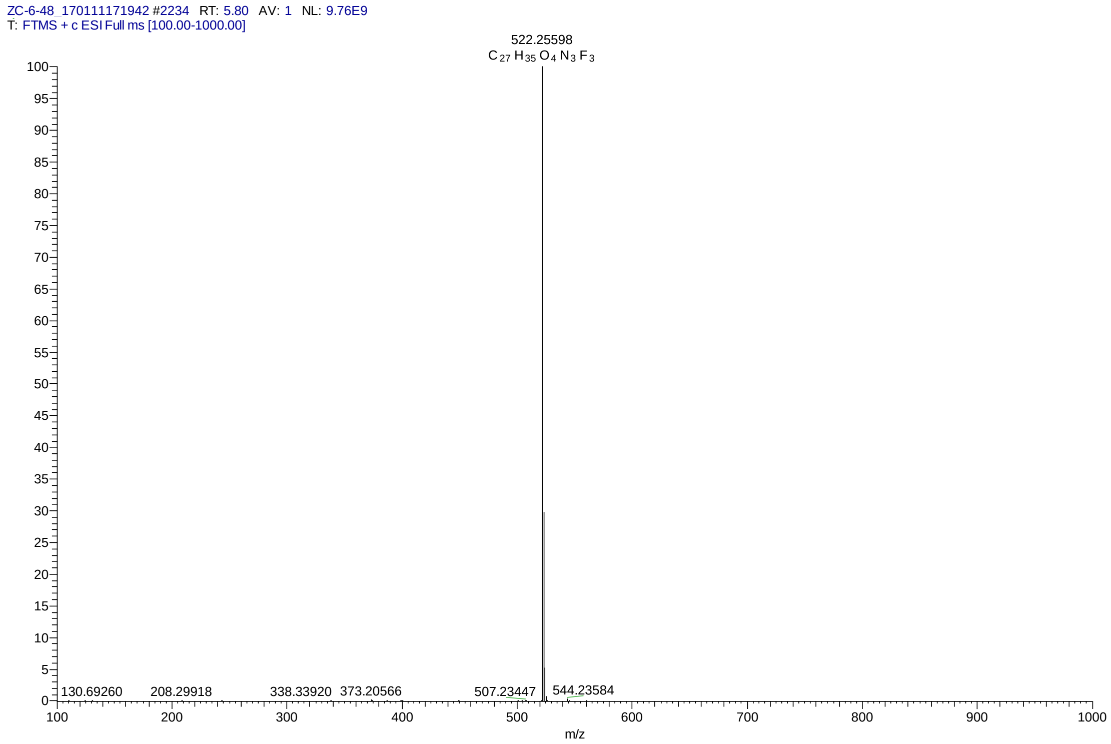

m/z	Theo. Mass	Delta (ppm)	RDB equiv.	Composition
522.25598	522.25742	-2.75	10.5	C27 H35 O4 N3 F3

^{1}H NMR spectra of compound i13

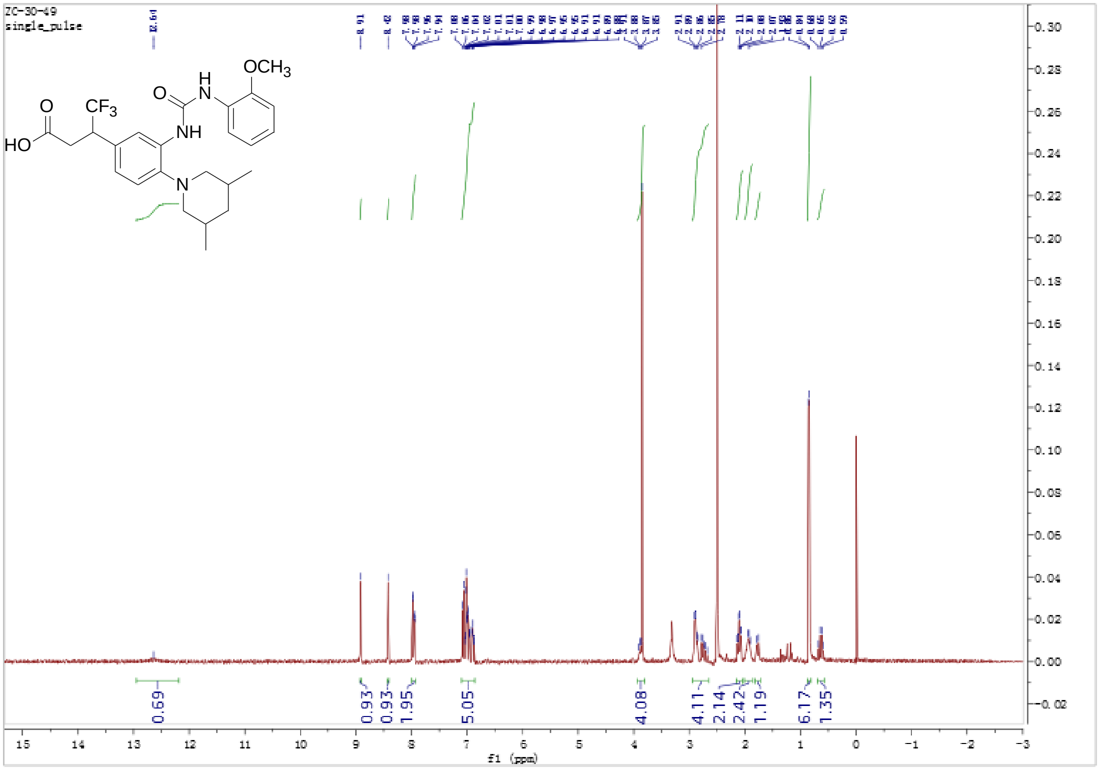

ESI-MS spectra of compound i13

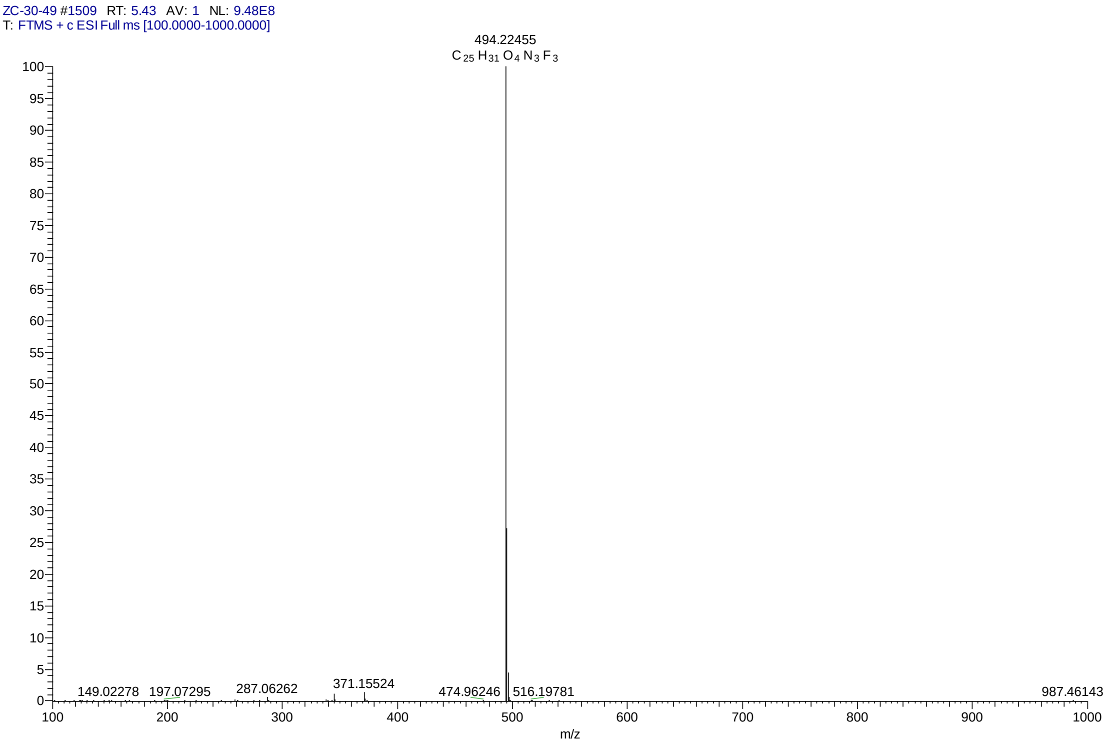

m/z	Theo. Mass	Delta (ppm)	RDB equiv.	Composition
494.22455	494.22612	-3.17	10.5	C25 H31 O4 N3 F3

^{1}H NMR spectra of compound g16

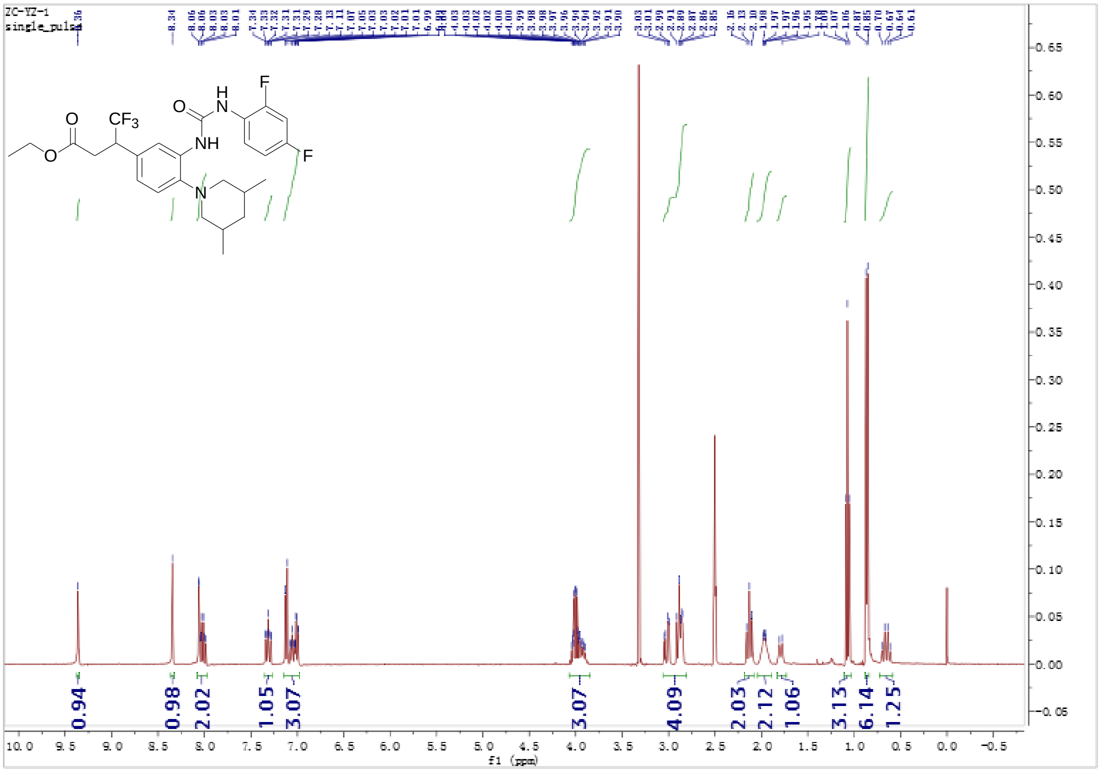

ESI-MS spectra of compound g16

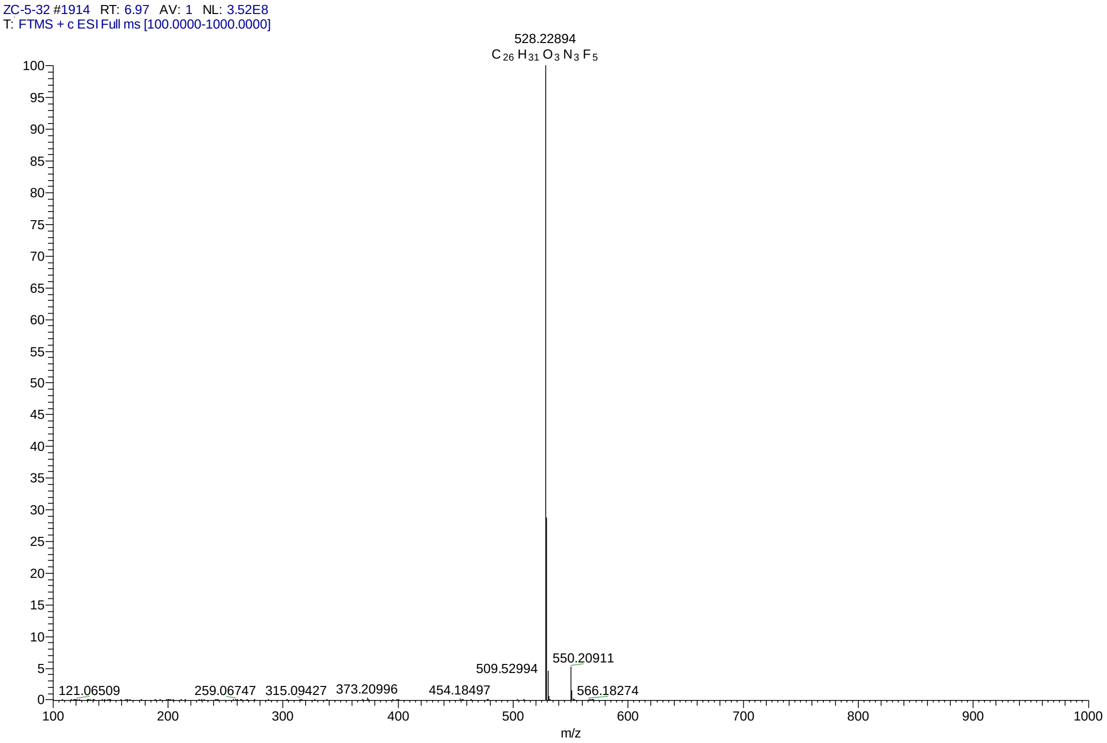

m/z	Theo. Mass	Delta (ppm)	RDB equiv.	Composition
528.22894	528.22801	1.76	10.5	C26 H31 O3 N3 F5

^{1}H NMR spectra of compound i16

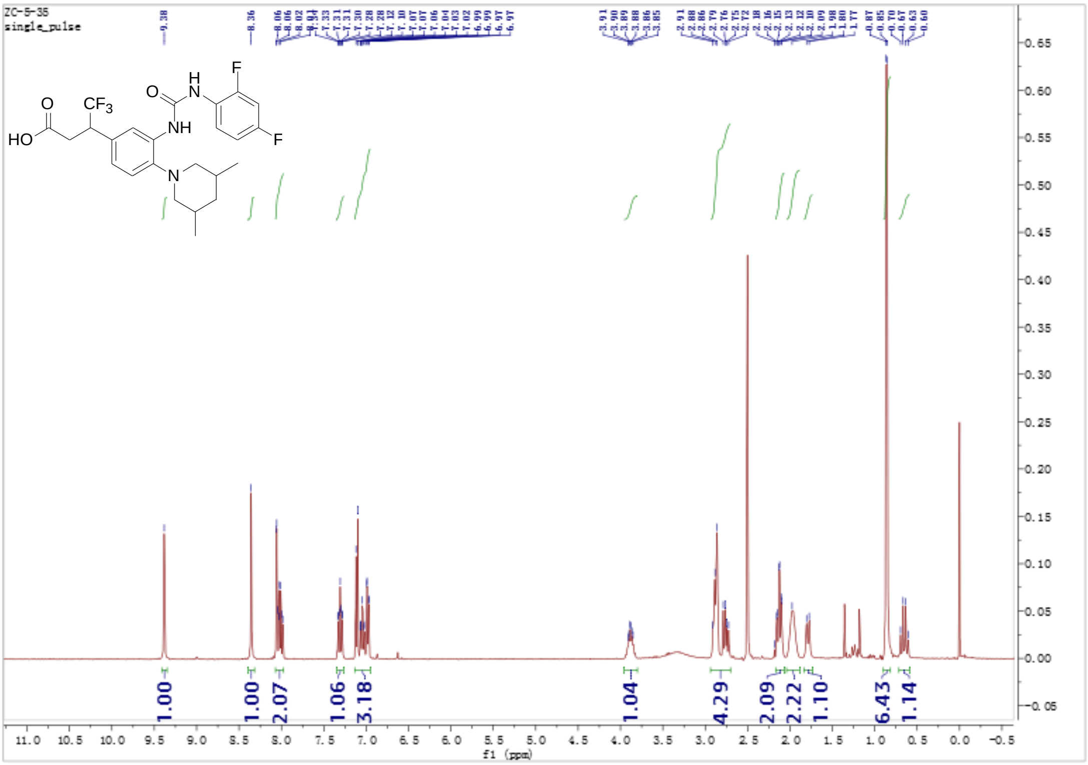

^{13}C NMR spectra of compound i16

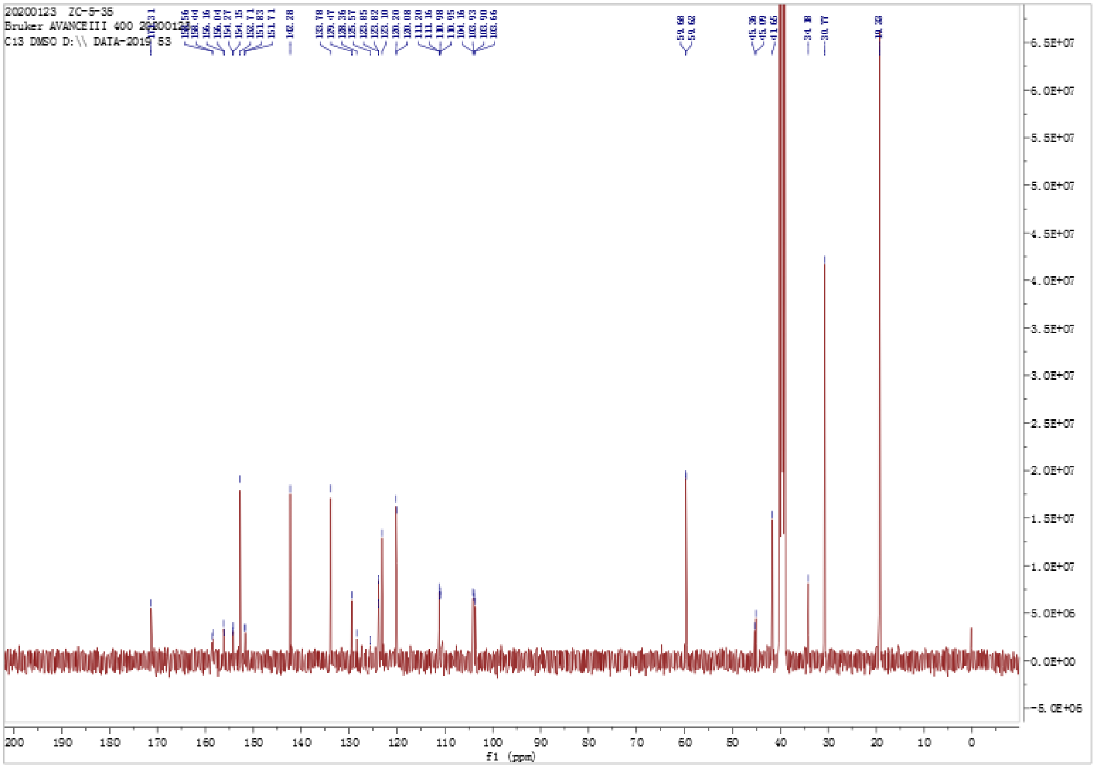

ESI-MS spectra of compound i16

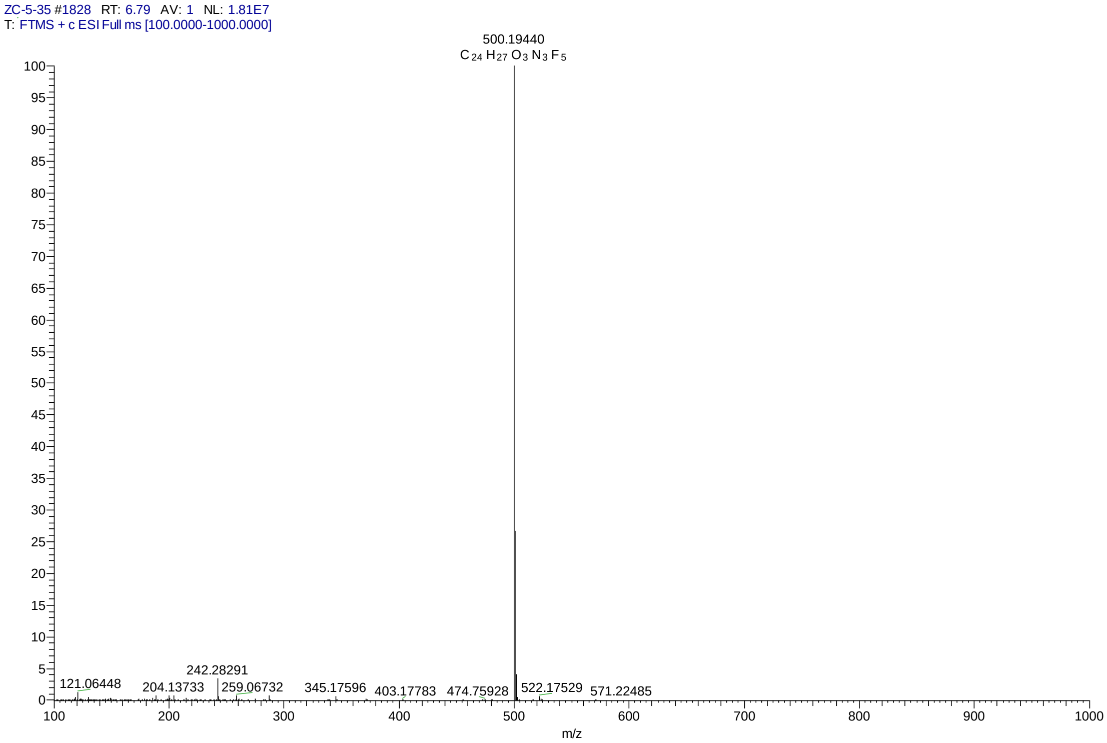

m/z	Theo. Mass	Delta (ppm)	RDB equiv.	Composition
500.19440	500.19671	-4.62	10.5	C24 H27 O3 N3 F5

^{1}H NMR spectra of compound g17

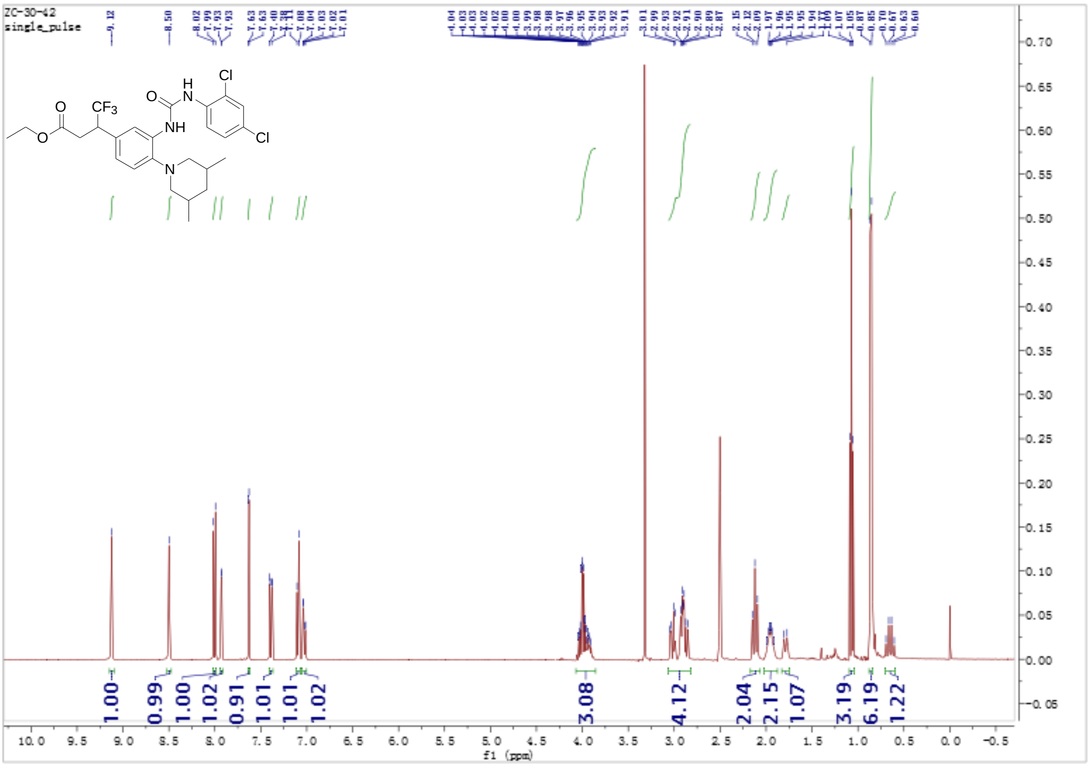

ESI-MS spectra of compound g17

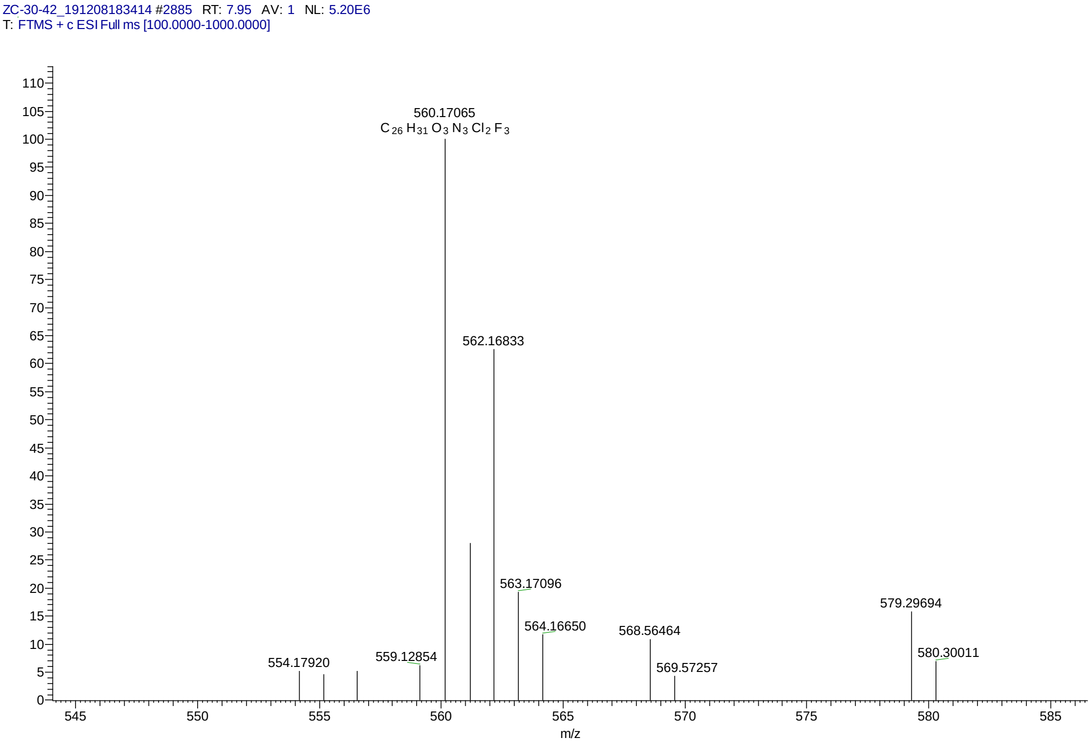

m/z	Theo. Mass	Delta (ppm)	RDB equiv.	Composition
560.17065	560.16891	3.11	10.5	C26 H31 O3 N3 Cl2 F3

^{1}H NMR spectra of compound i17

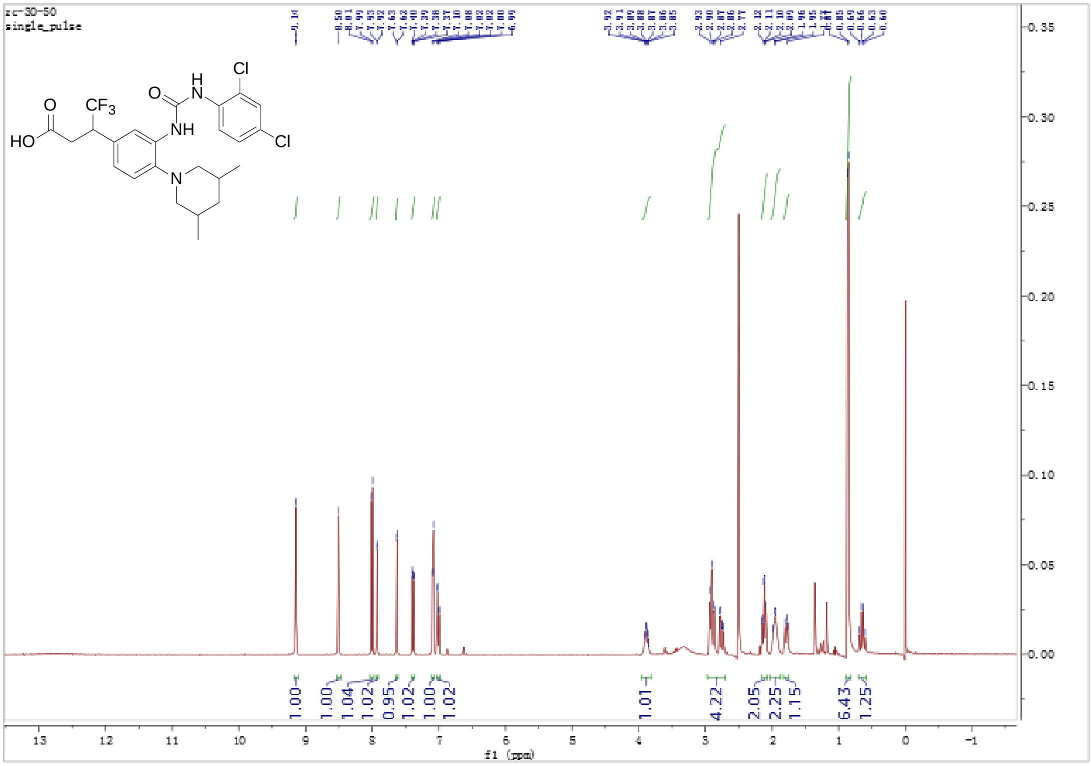

^{13}C NMR spectra of compound i17

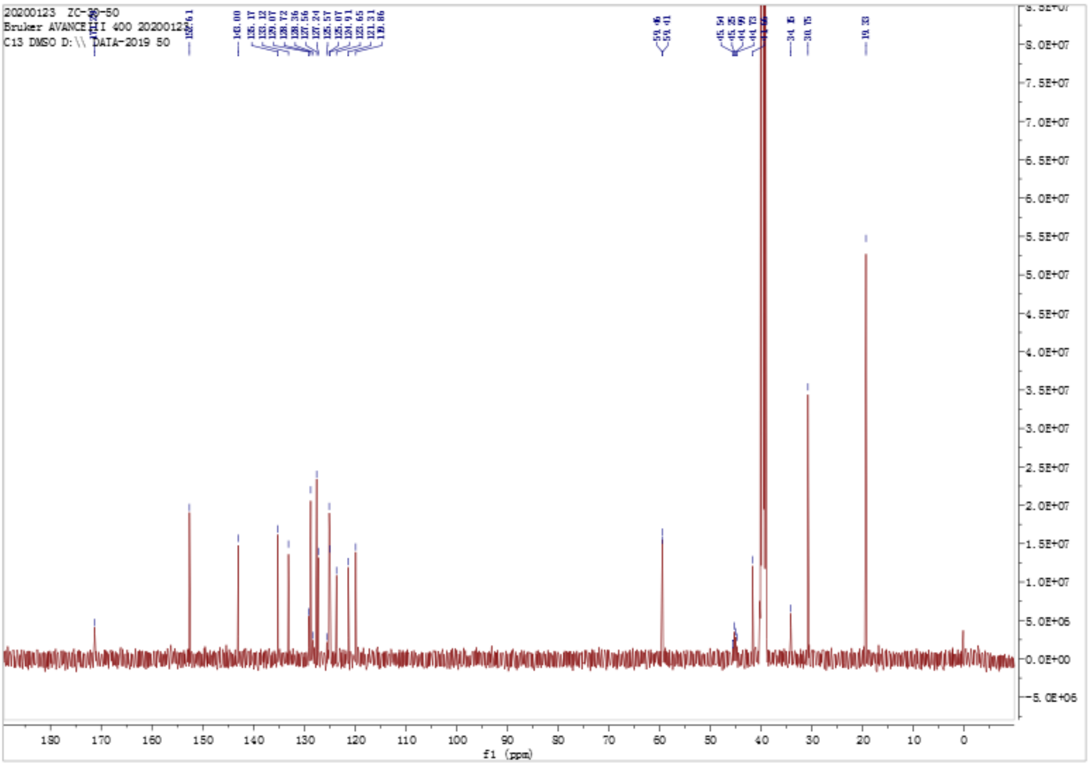

ESI-MS spectra of compound i17

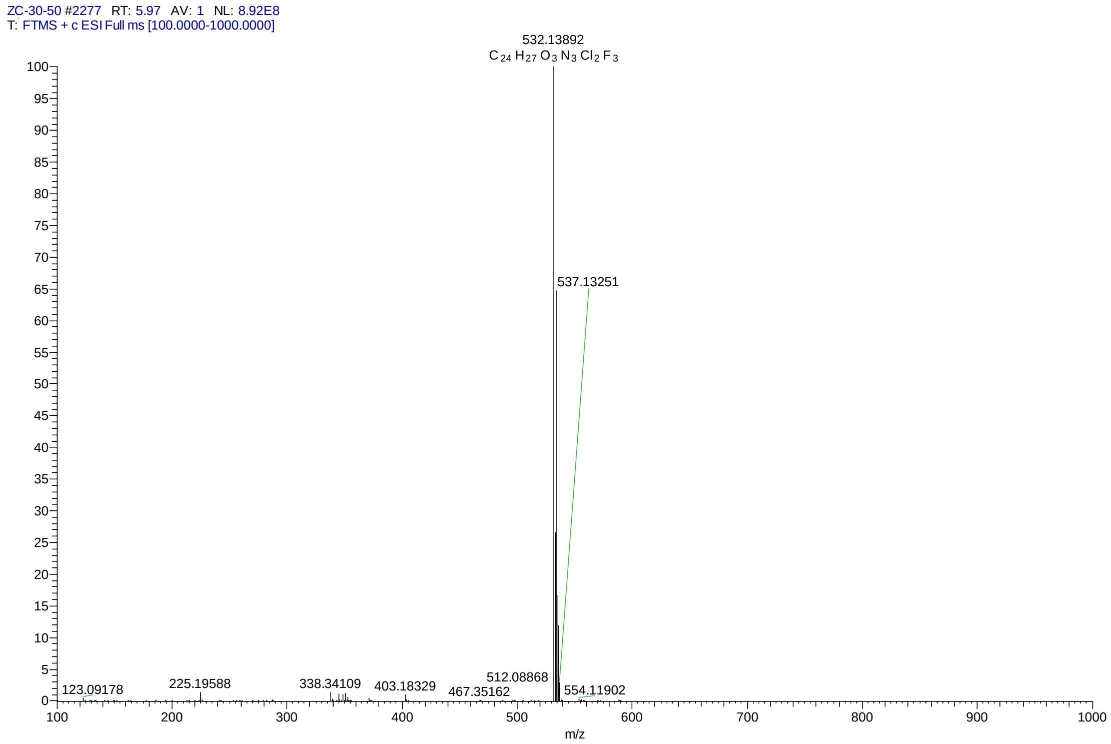

m/z	Theo. Mass	Delta (ppm)	RDB equiv.	Composition
532.13892	532.13761	2.47	10.5	C24 H27 O3 N3 Cl2 F3

^{1}H NMR spectra of compound g18

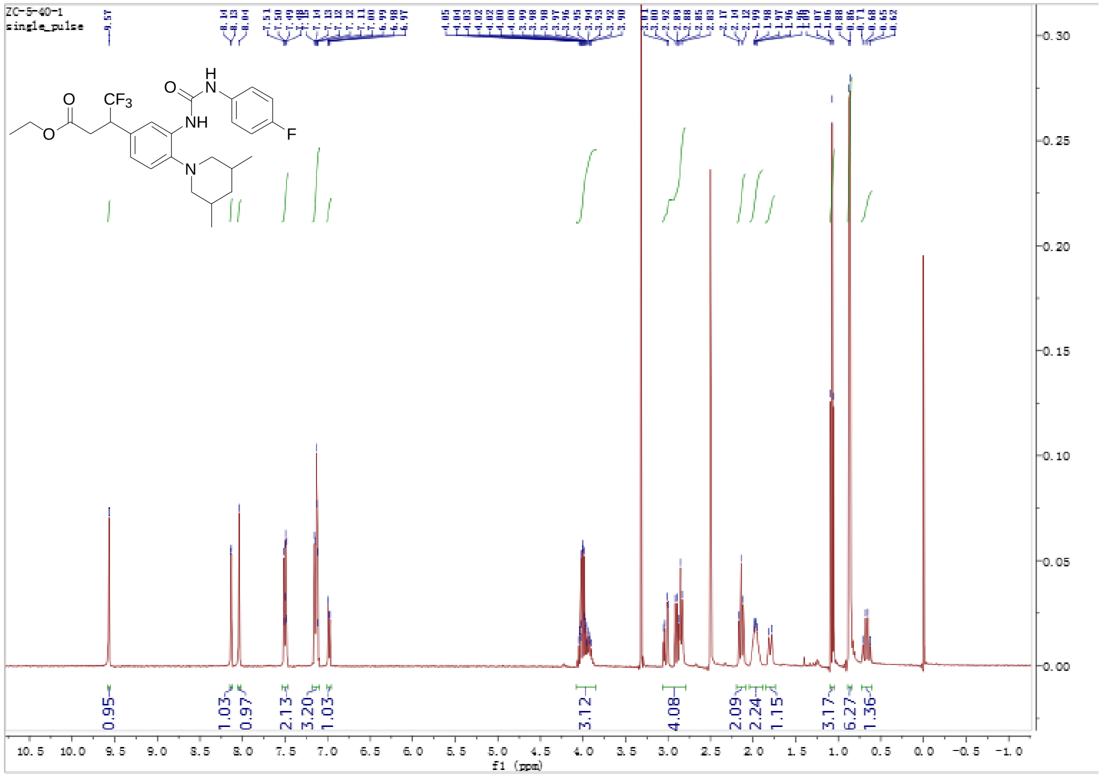

^{13}C NMR spectra of compound g18

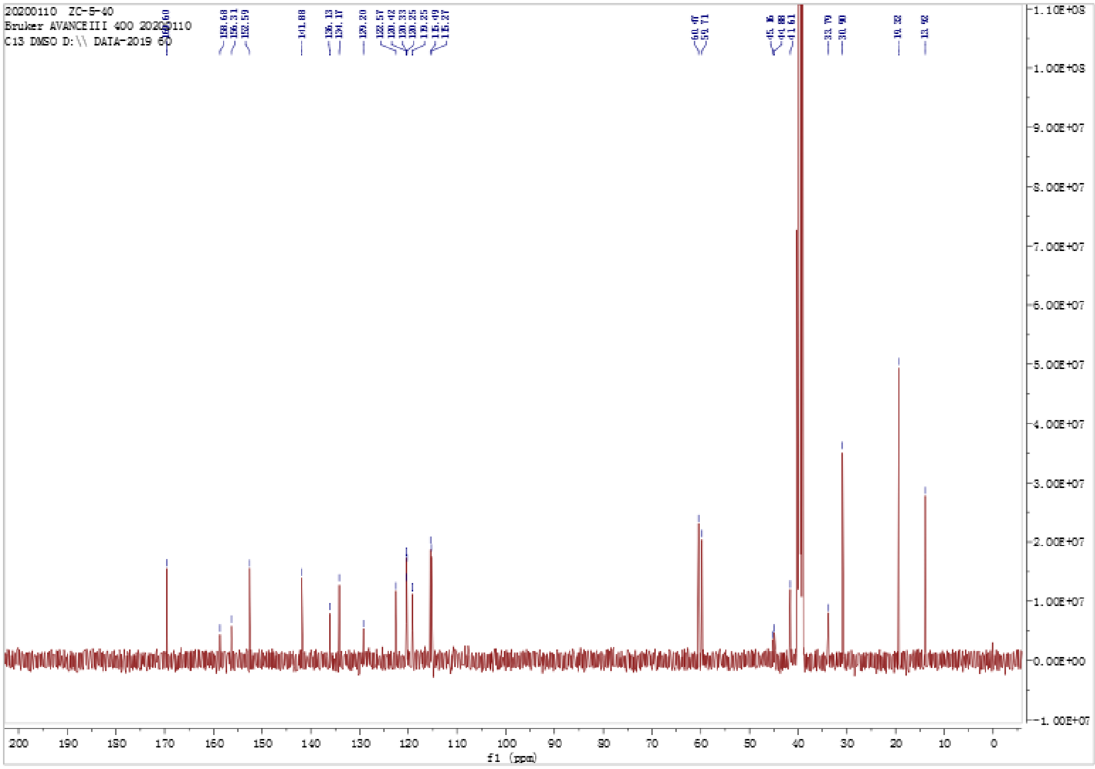

ESI-MS spectra of compound g18

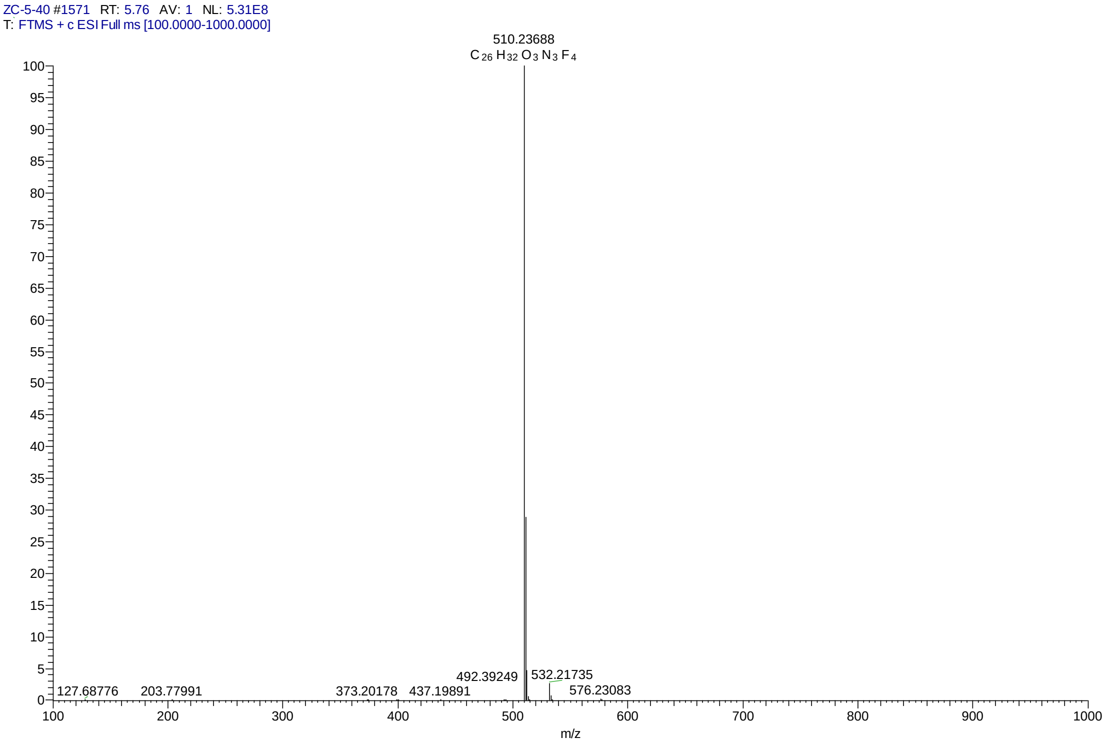

m/z	Theo. Mass	Delta (ppm)	RDB equiv.	Composition
510.23688	510.23743	-1.08	10.5	C26 H32 O3 N3 F4

^{1}H NMR spectra of compound i18

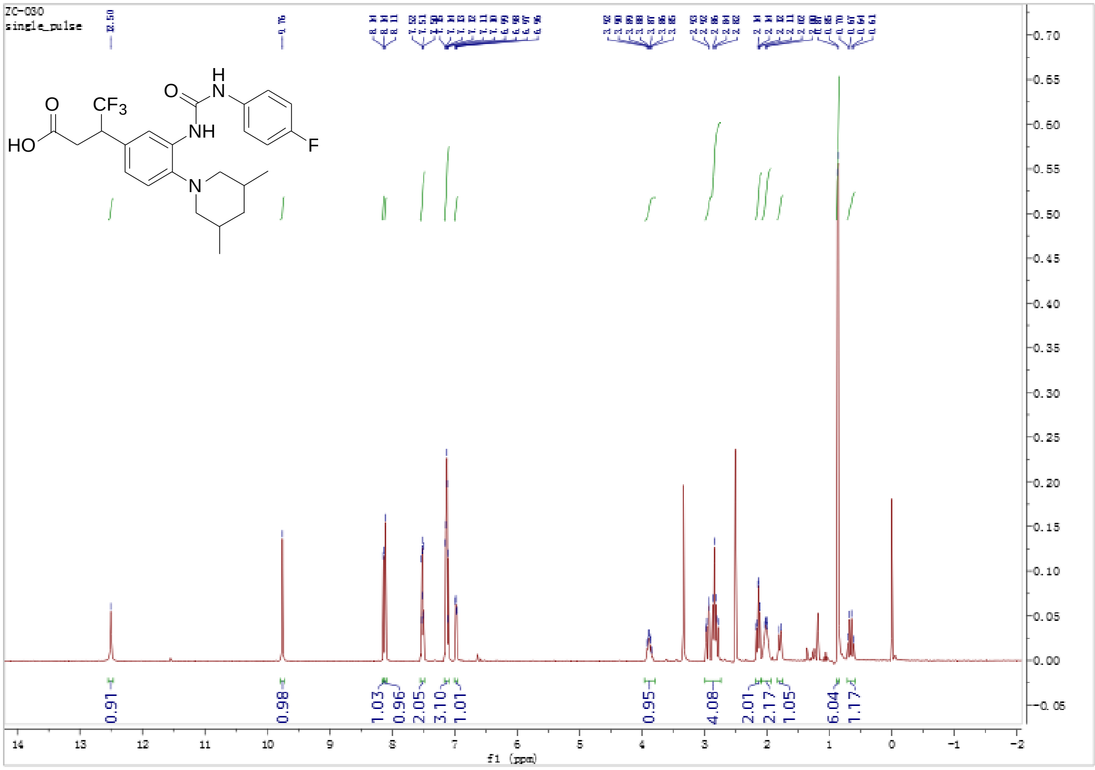

ESI-MS spectra of compound i18

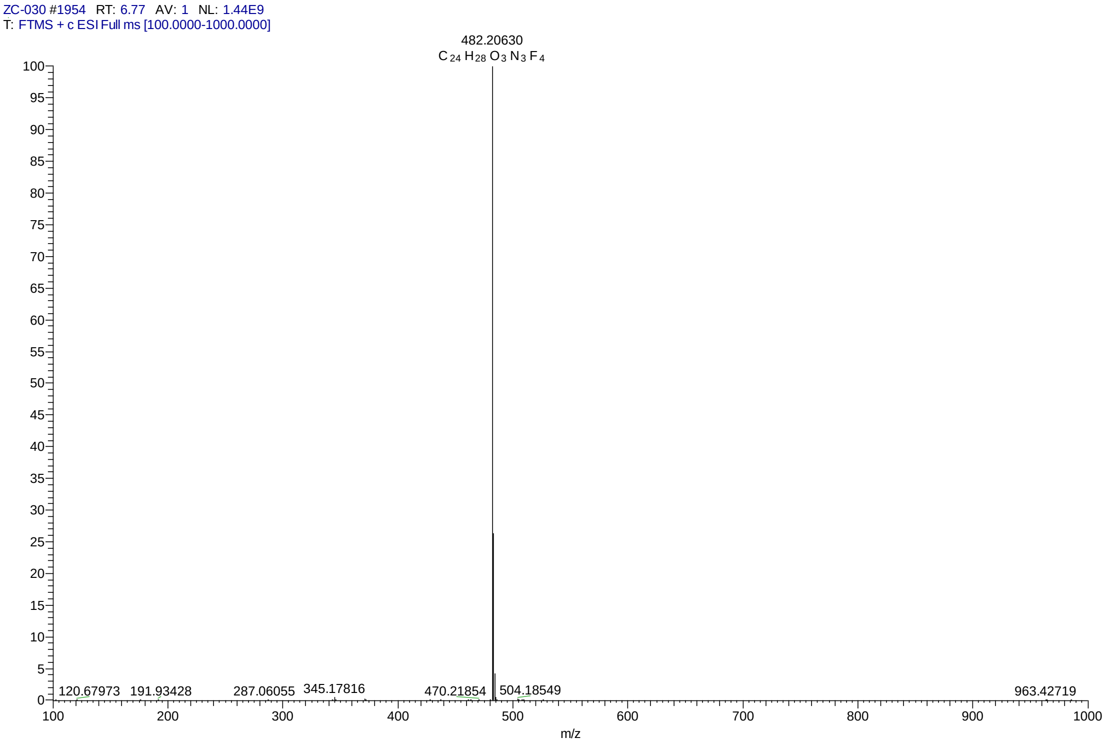

m/z	Theo. Mass	Delta (ppm)	RDB equiv.	Composition
482.20630	482.20613	0.35	10.5	C24 H28 O3 N3 F4

^{1}H NMR spectra of compound g19

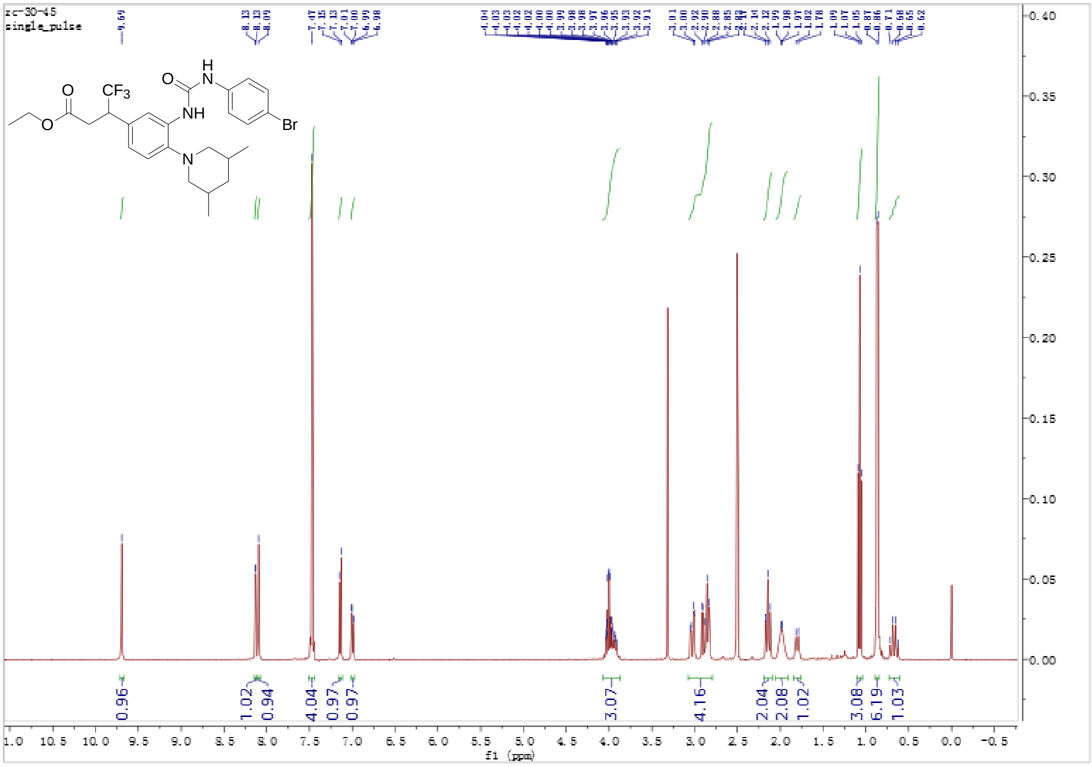

ESI-MS spectra of compound g19

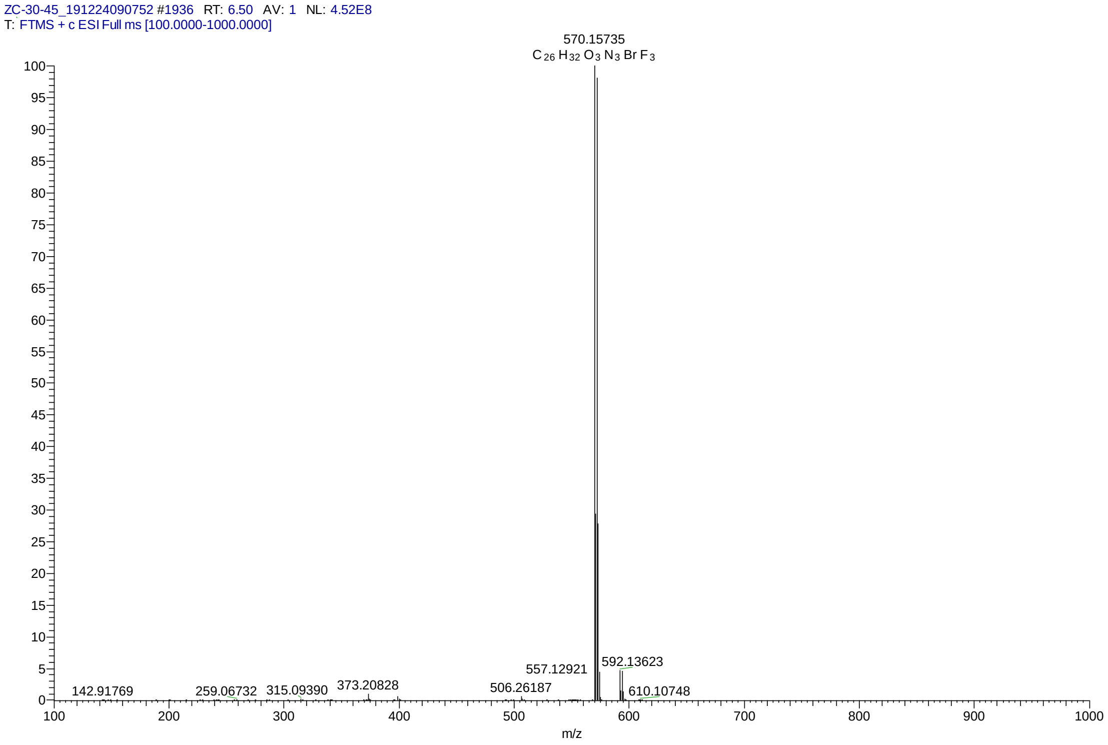

m/z	Theo. Mass	Delta (ppm)	RDB equiv.	Composition
570.15735	570.15737	-0.03	10.5	C26 H32 O3 N3 Br F3

^{1}H NMR spectra of compound i19

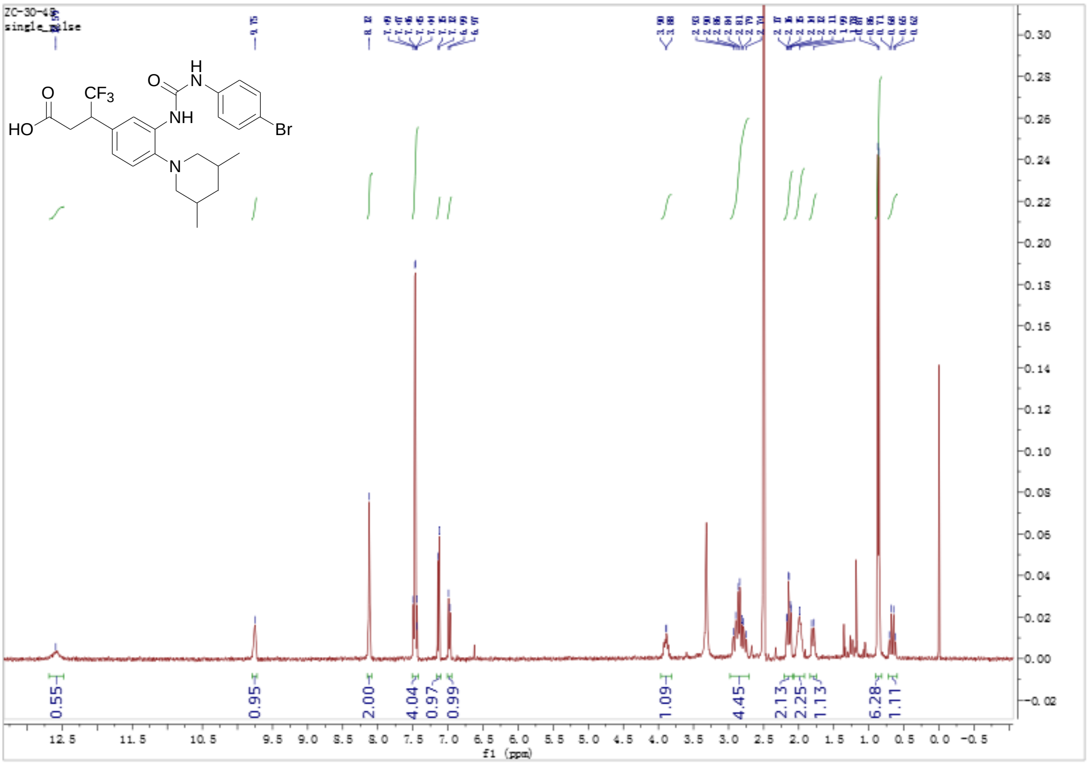

^{13}C NMR spectra of compound i19

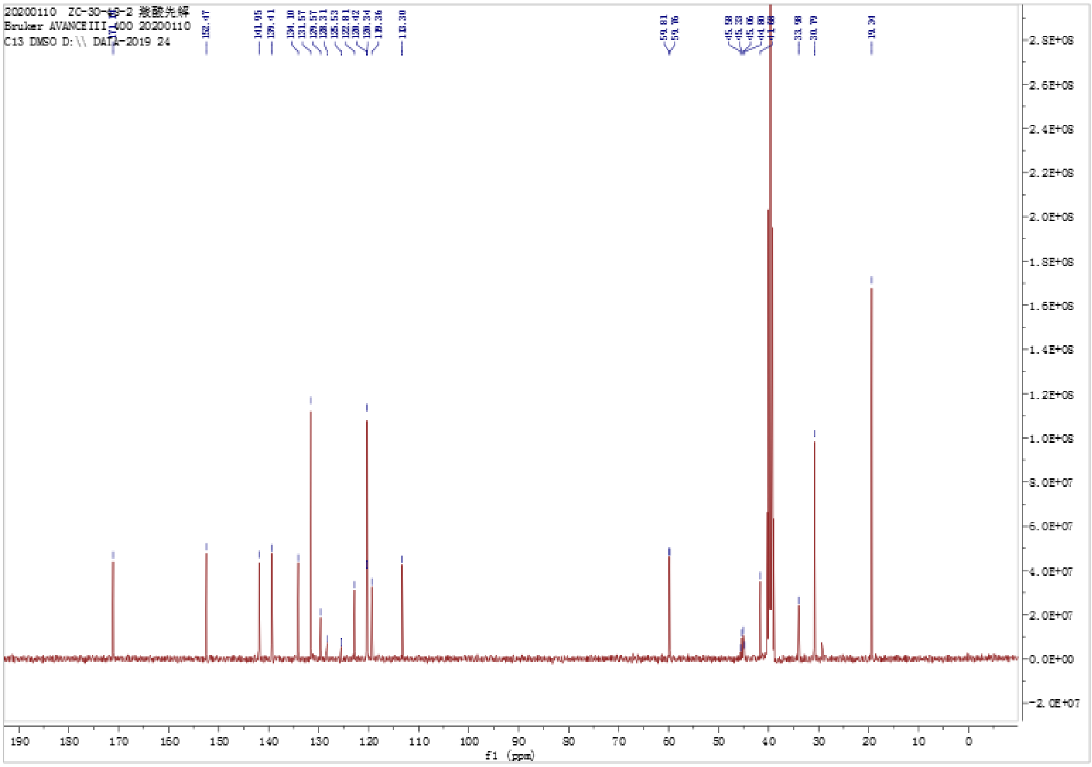

ESI-MS spectra of compound i19

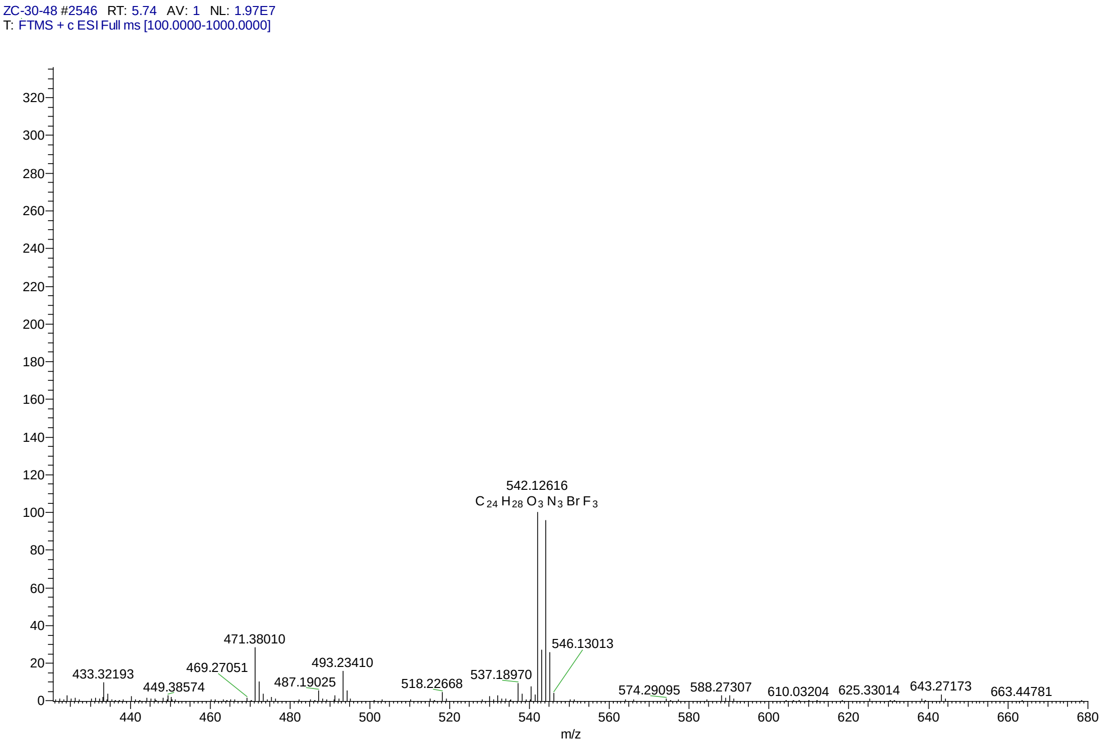

m/z	Theo. Mass	Delta (ppm)	RDB equiv.	Composition
542.12616	542.12607	0.17	10.5	C24 H28 O3 N3 Br F3

^{1}H NMR spectra of compound g20

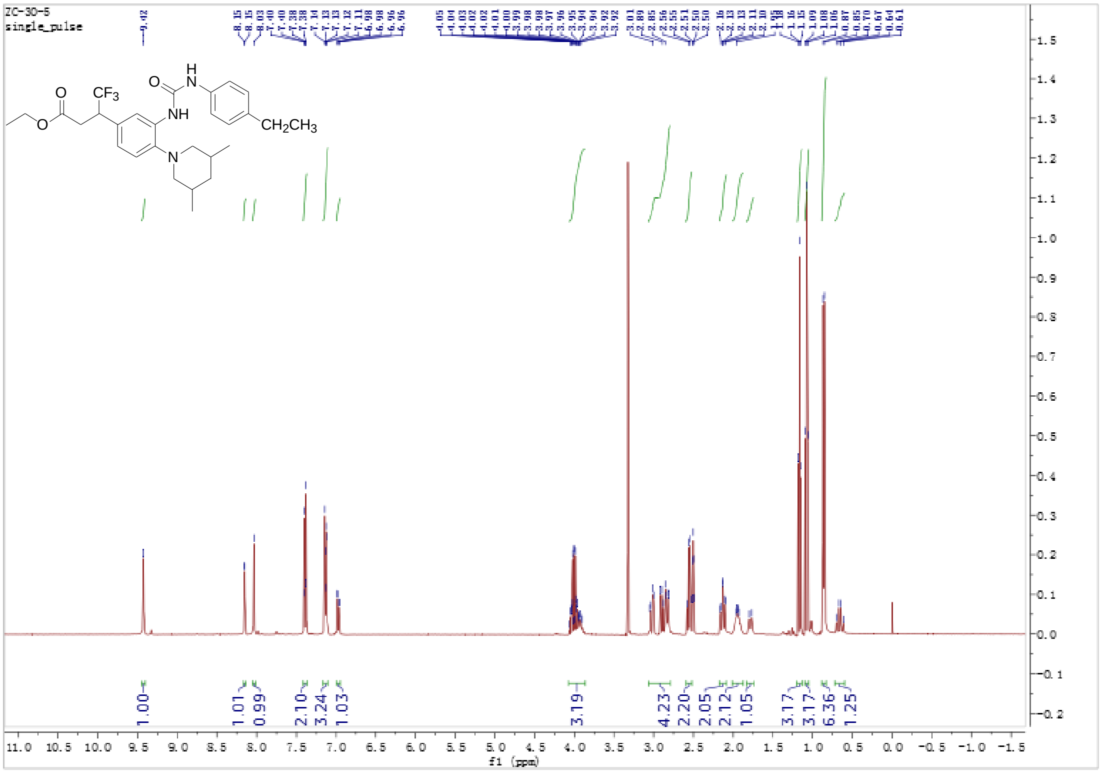

ESI-MS spectra of compound g20

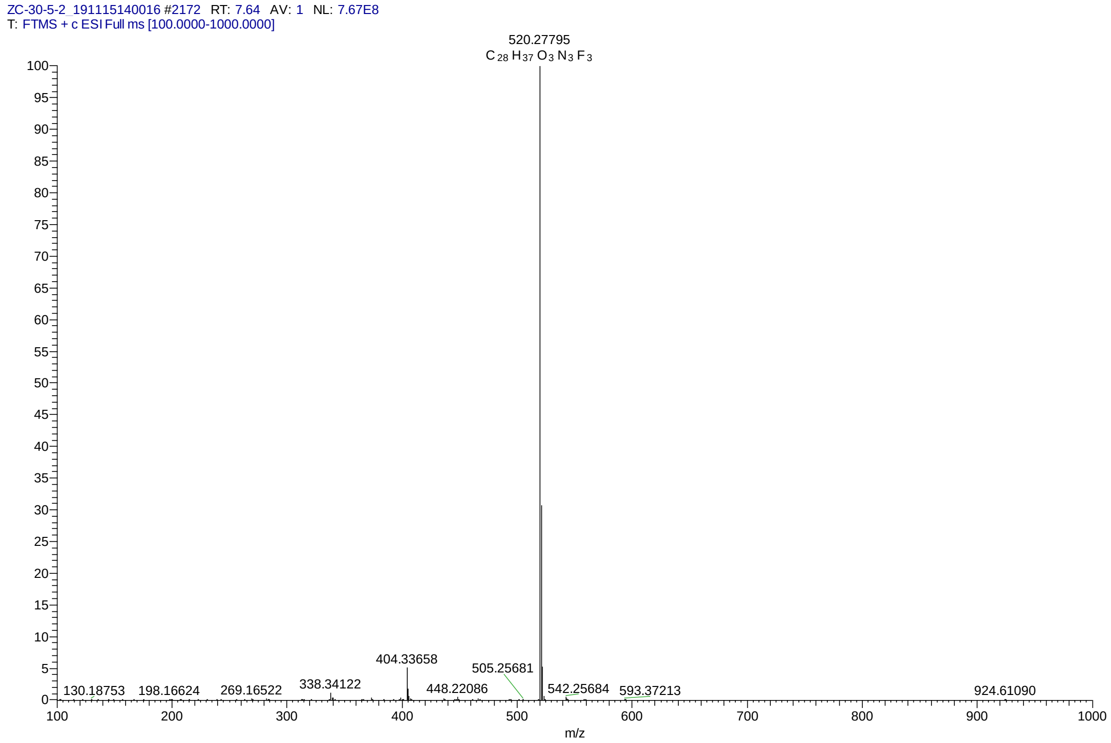

m/z	Theo. Mass	Delta (ppm)	RDB equiv.	Composition
520.27795	520.27815	-0.39	10.5	C28 H37 O3 N3 F3

^{1}H NMR spectra of compound i20

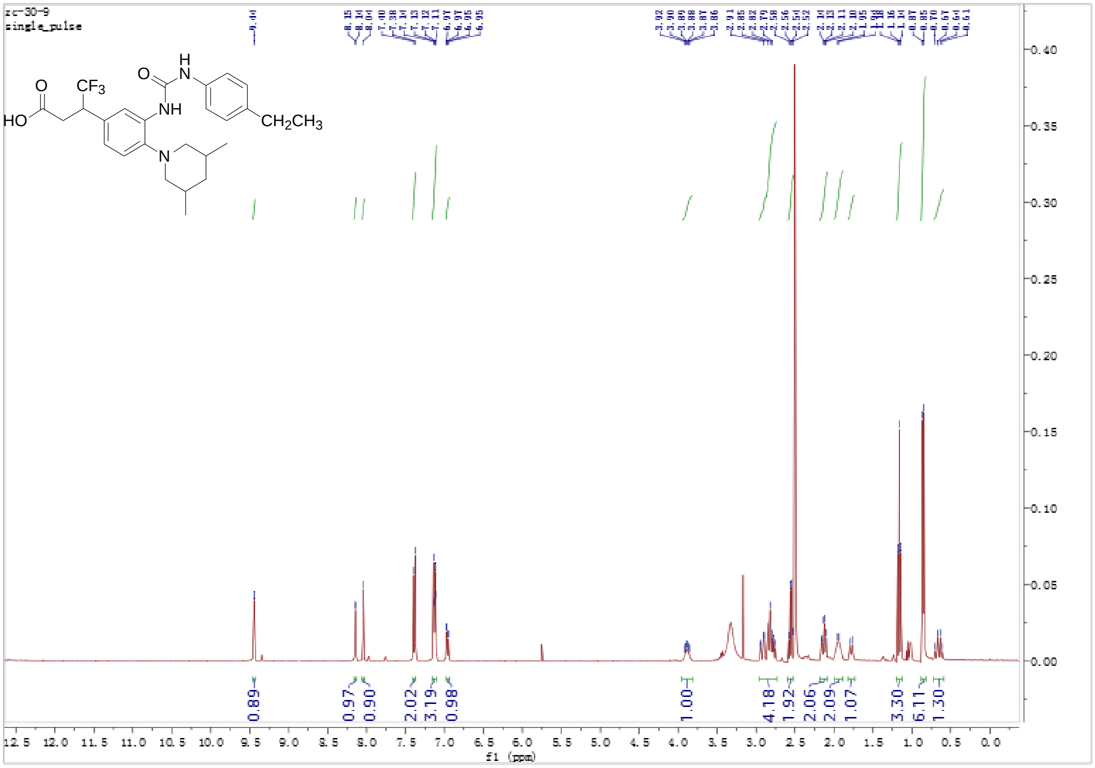

^{13}C NMR spectra of compound i20

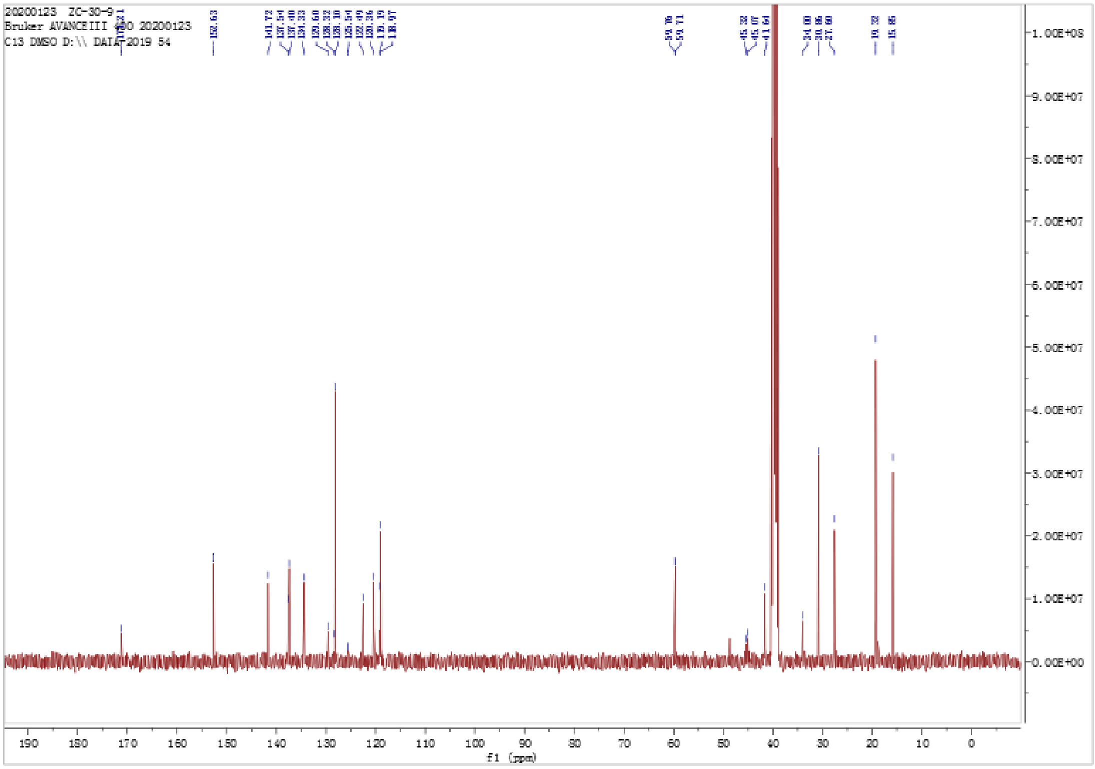

ESI-MS spectra of compound i20

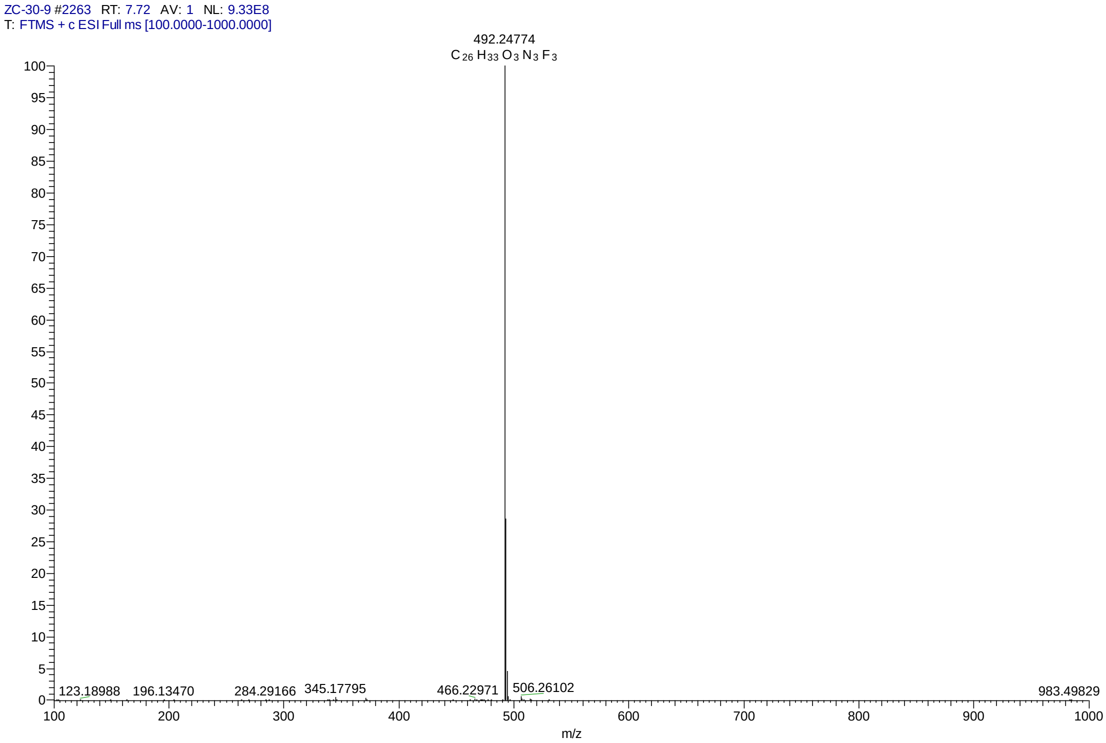

m/z	Theo. Mass	Delta (ppm)	RDB equiv.	Composition
492.24774	492.24685	1.80	10.5	C26 H33 O3 N3 F3

^{1}H NMR spectra of compound g21

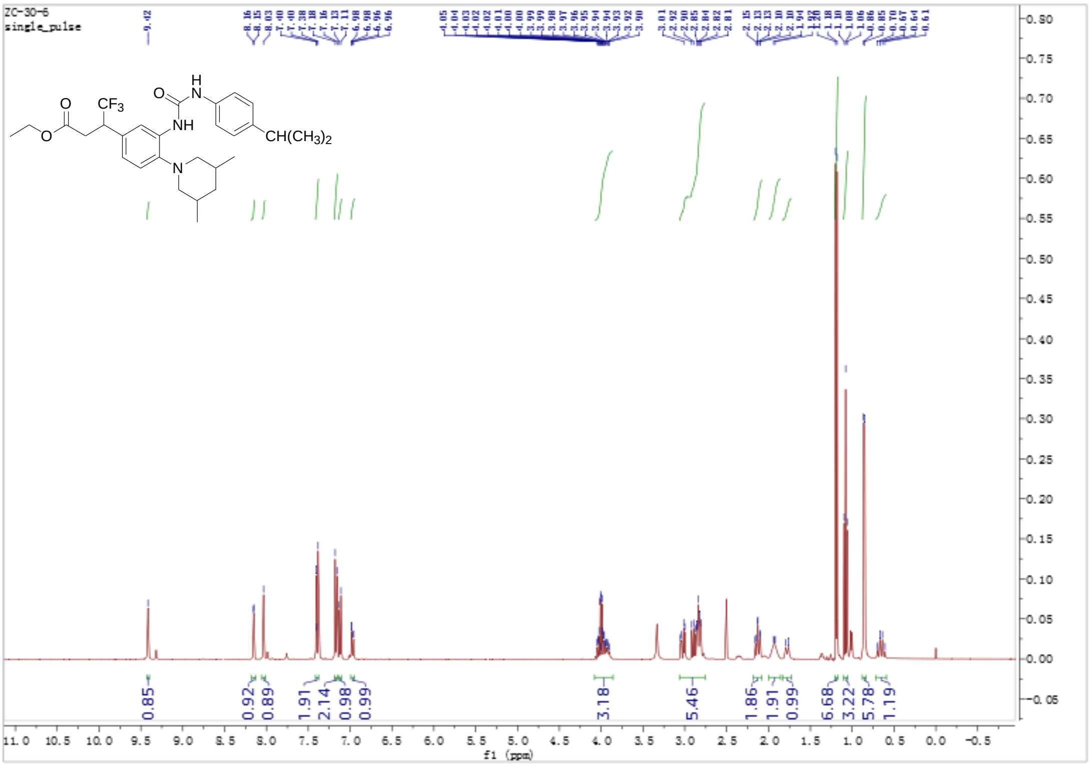

ESI-MS spectra of compound g21

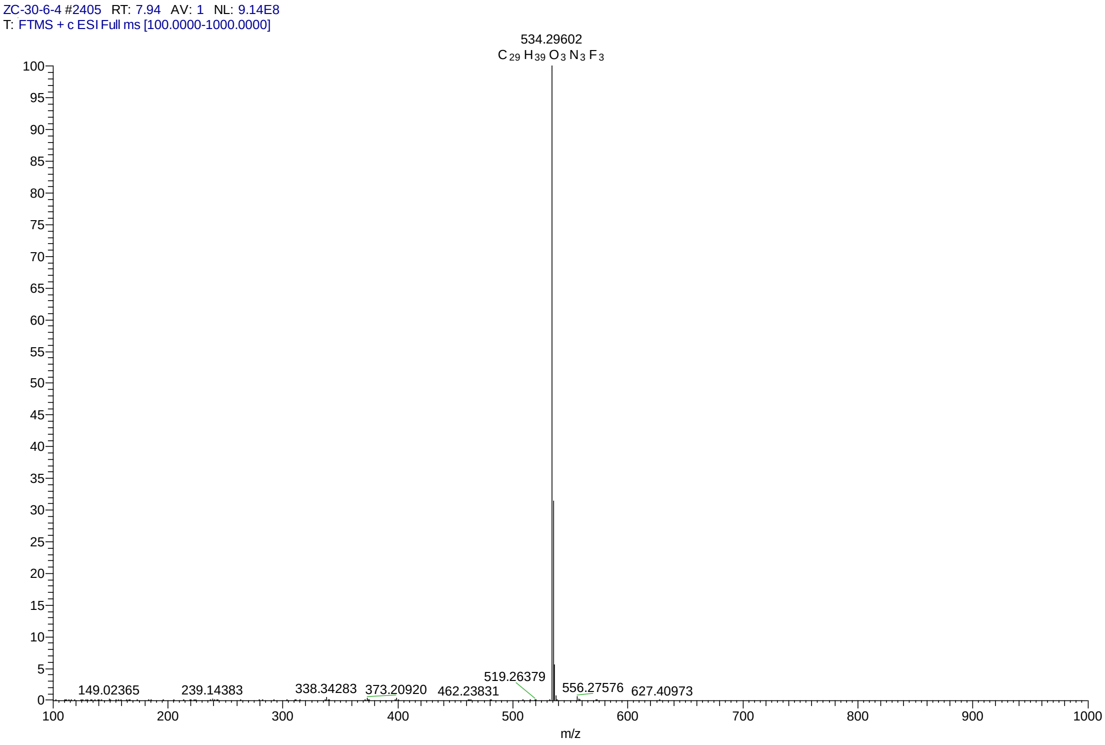

m/z	Theo. Mass	Delta (ppm)	RDB equiv.	Composition
534.29602	534.29380	4.15	10.5	C29 H39 O3 N3 F3

^{1}H NMR spectra of compound i21

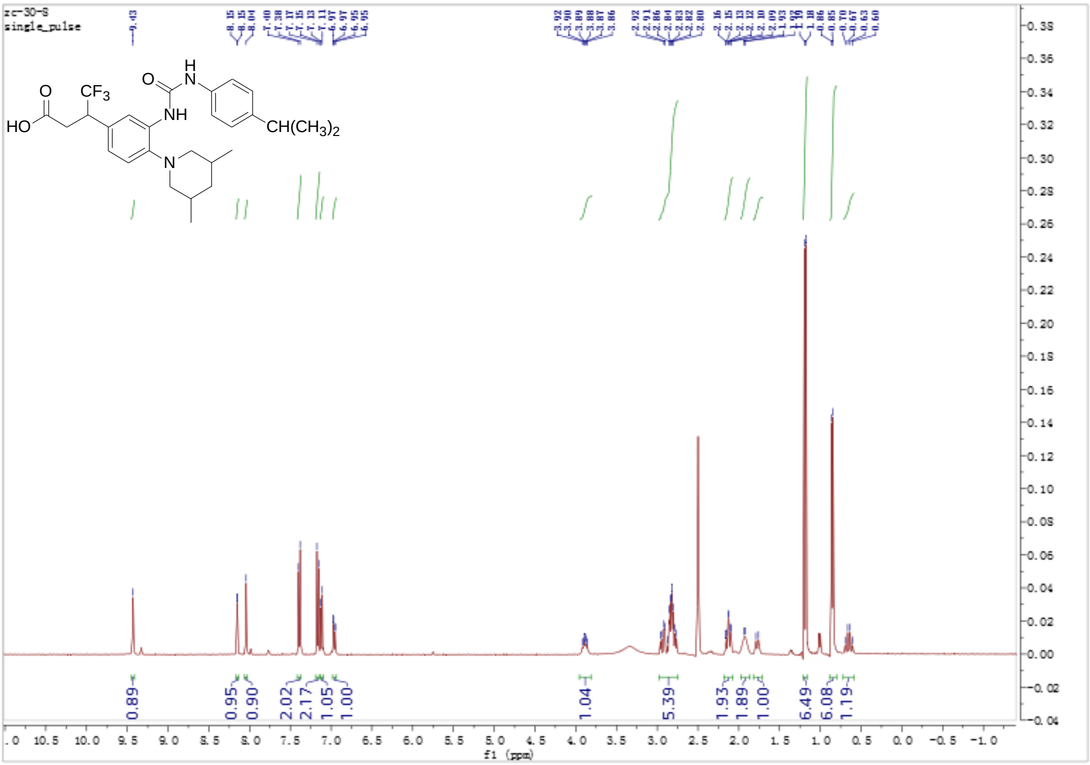

^{13}C NMR spectra of compound i21

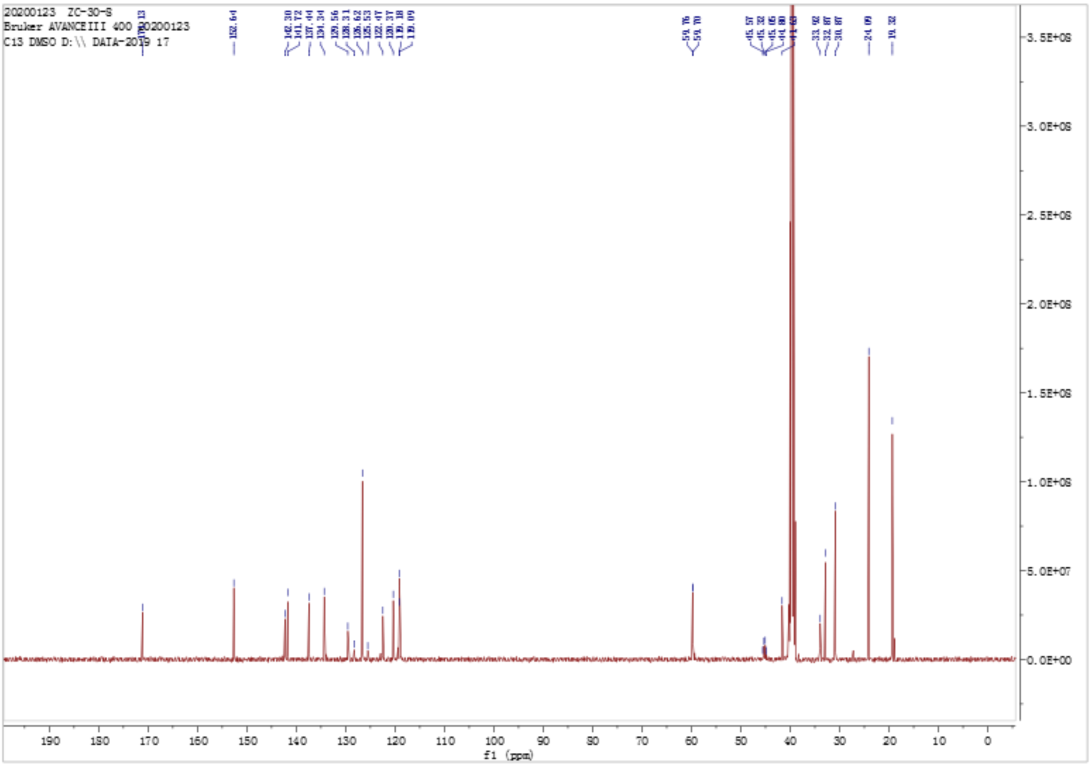

ESI-MS spectra of compound i21

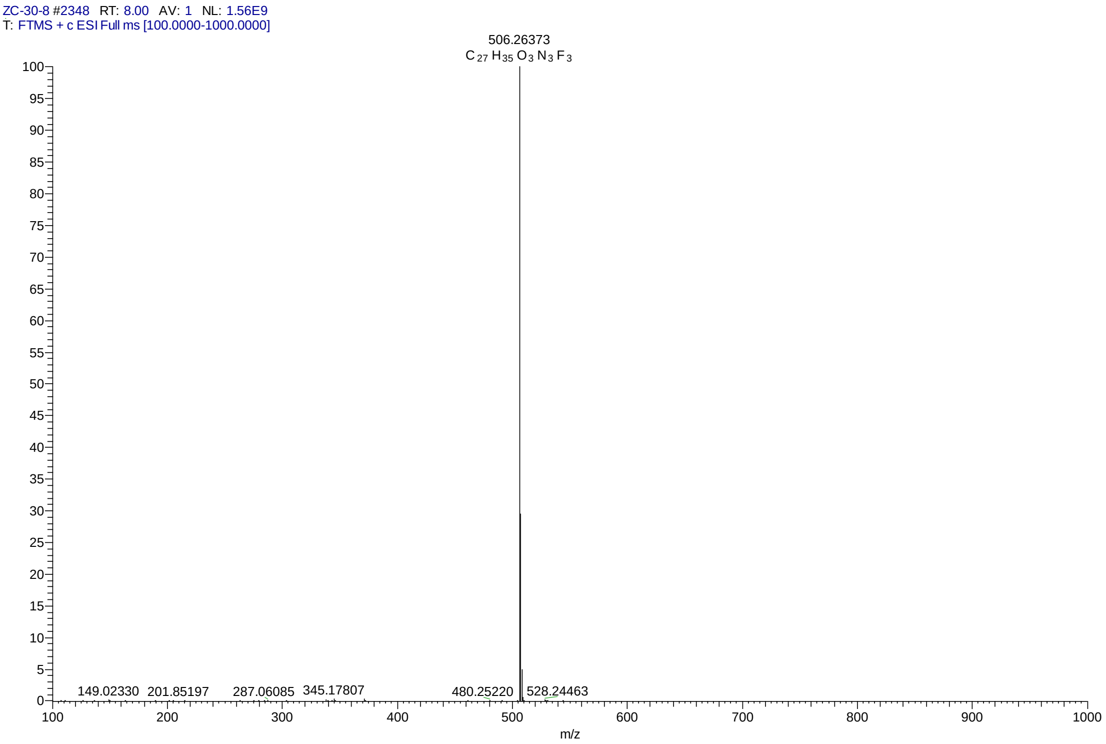

m/z	Theo. Mass	Delta (ppm)	RDB equiv.	Composition
506.26373	506.26250	2.42	10.5	C27 H35 O3 N3 F3

^{1}H NMR spectra of compound g22

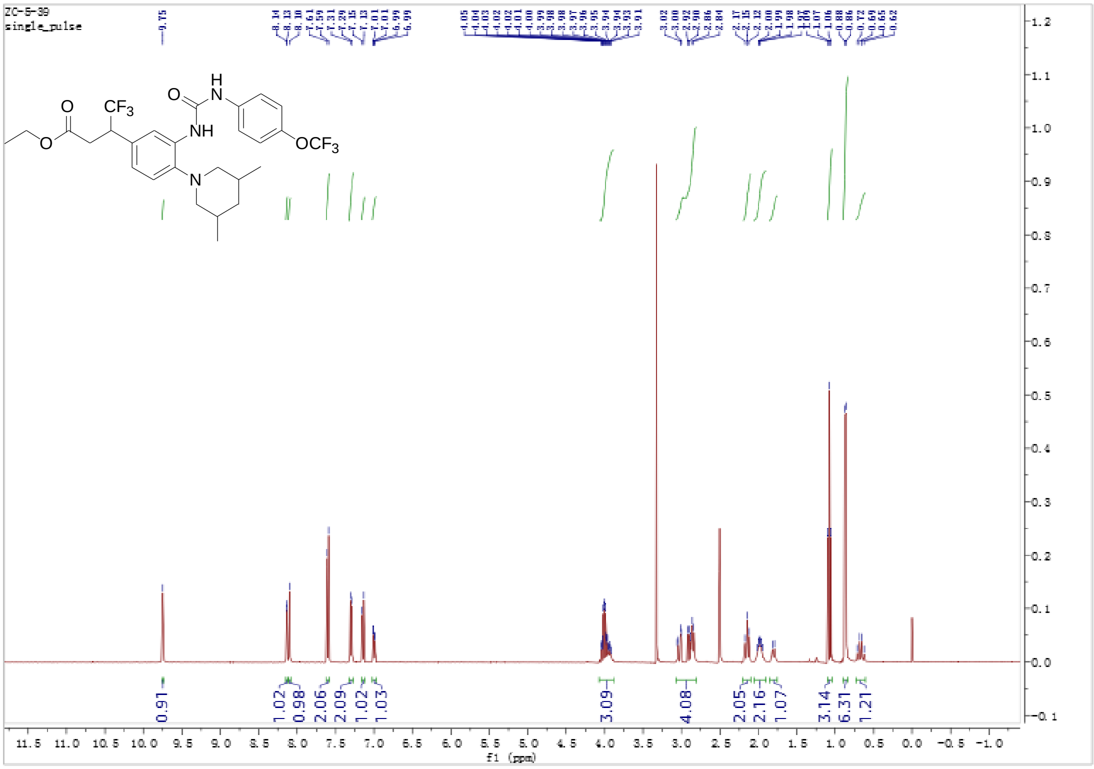

ESI-MS spectra of compound g22

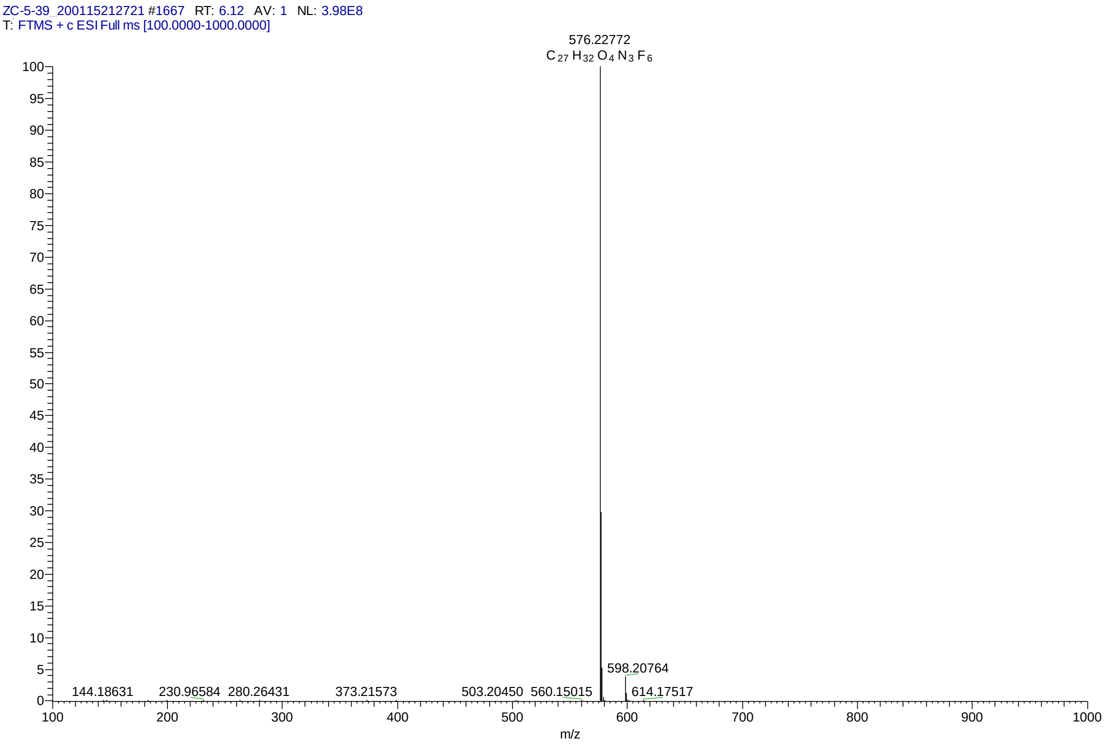

m/z	Theo. Mass	Delta (ppm)	RDB equiv.	Composition
576.22772	576.22915	-2.49	10.5	C27 H32 O4 N3 F6

^{1}H NMR spectra of compound i22

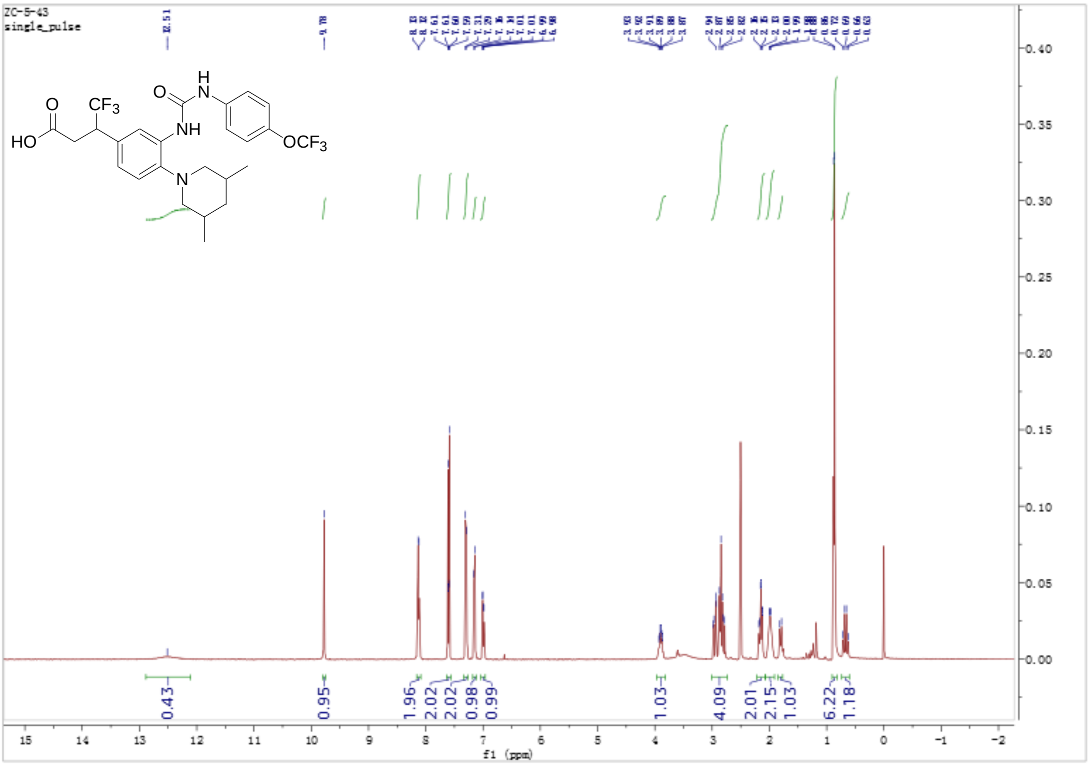

^{13}C NMR spectra of compound i22

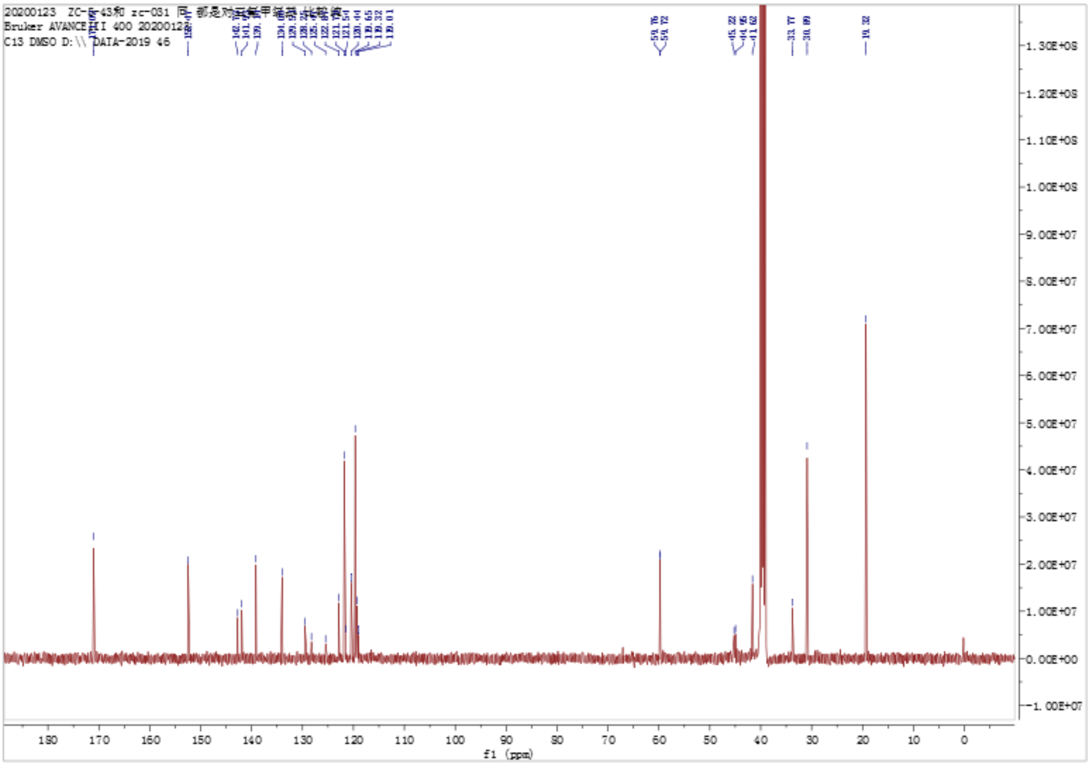

ESI-MS spectra of compound i22

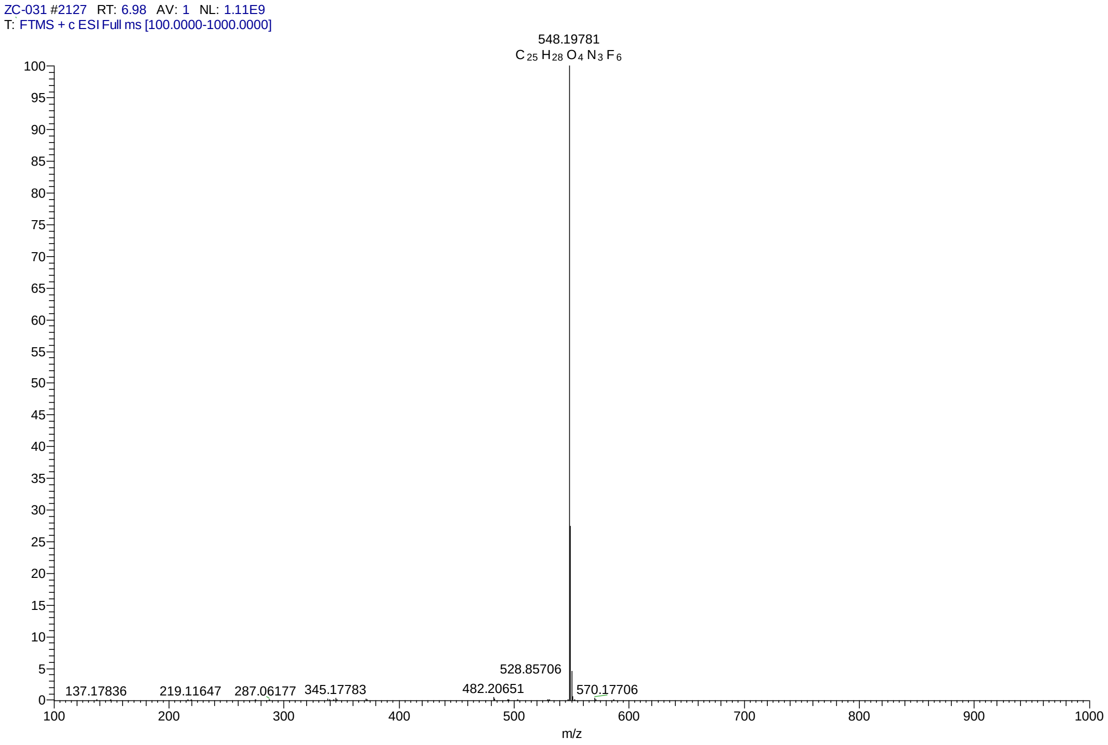

m/z	Theo. Mass	Delta (ppm)	RDB equiv.	Composition
548.19781	548.19785	-0.08	10.5	C25 H28 O4 N3 F6

^{1}H NMR spectra of compound g23

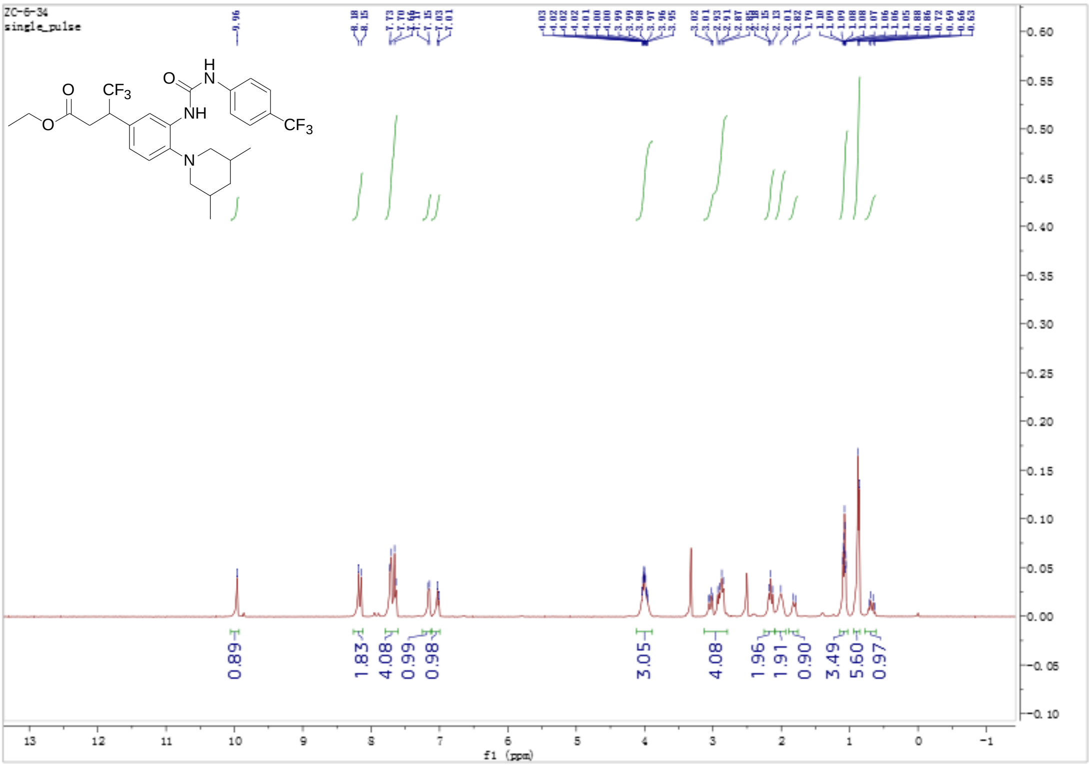

ESI-MS spectra of compound g23

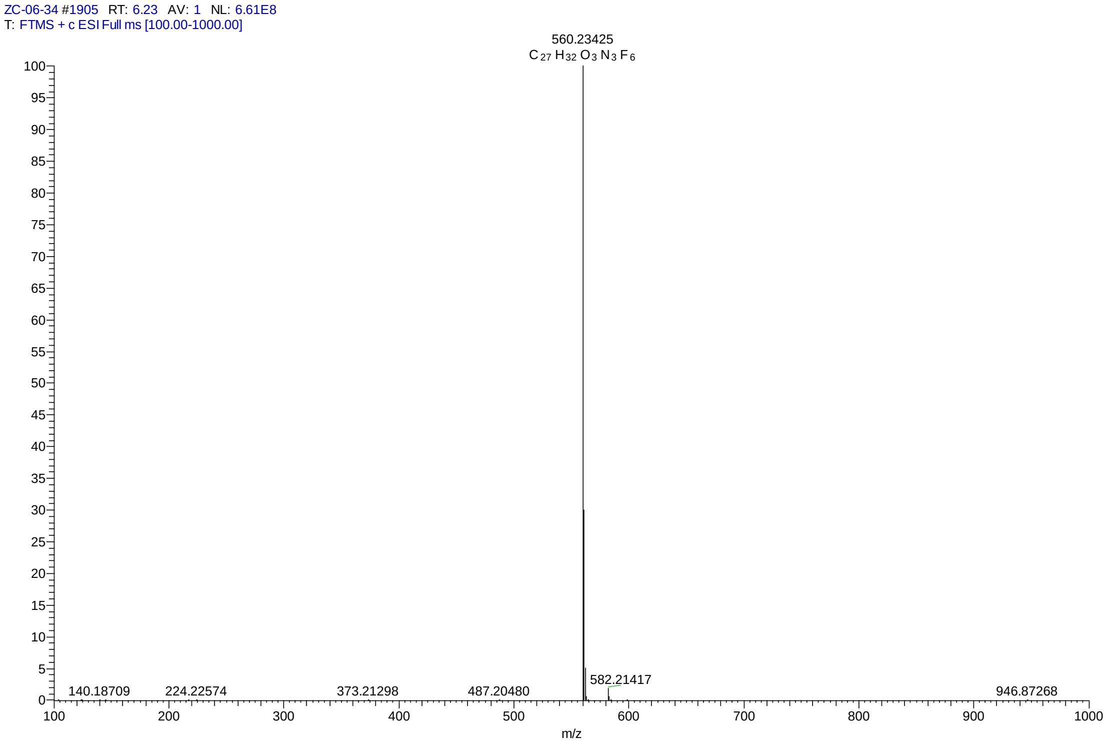

m/z	Theo. Mass	Delta (ppm)	RDB equiv.	Composition
560.23425	560.23424	0.02	10.5	C27 H32 O3 N3 F6

^{1}H NMR spectra of compound i23

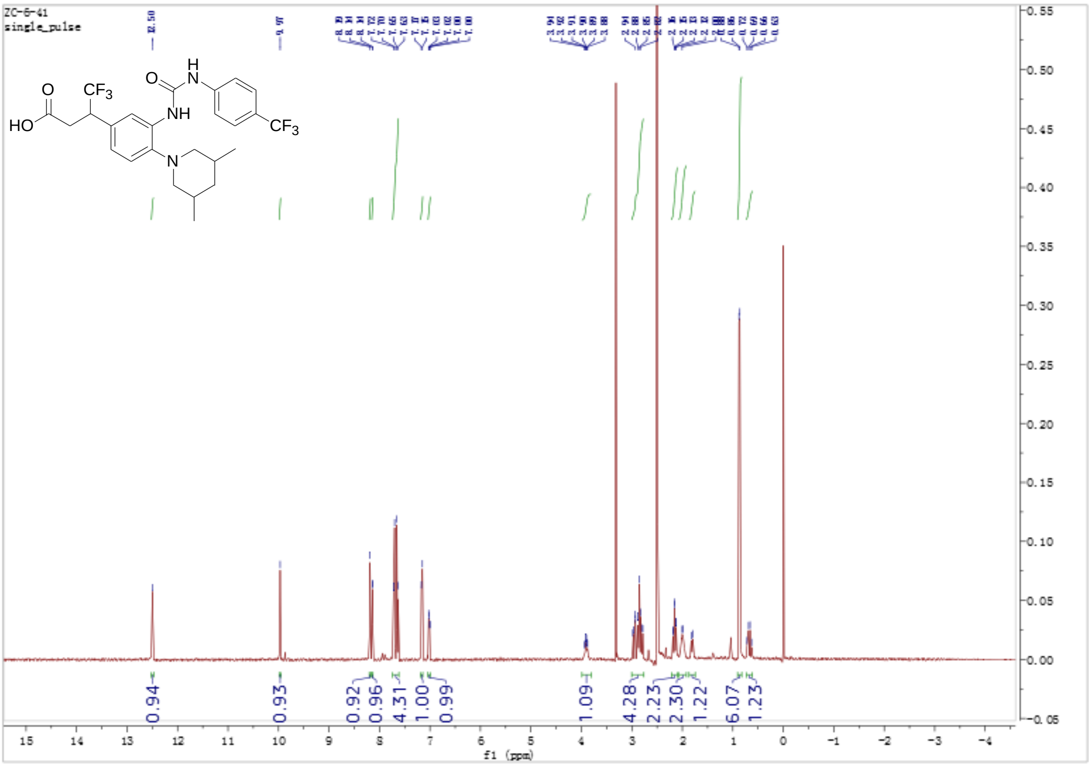

ESI-MS spectra of compound i23

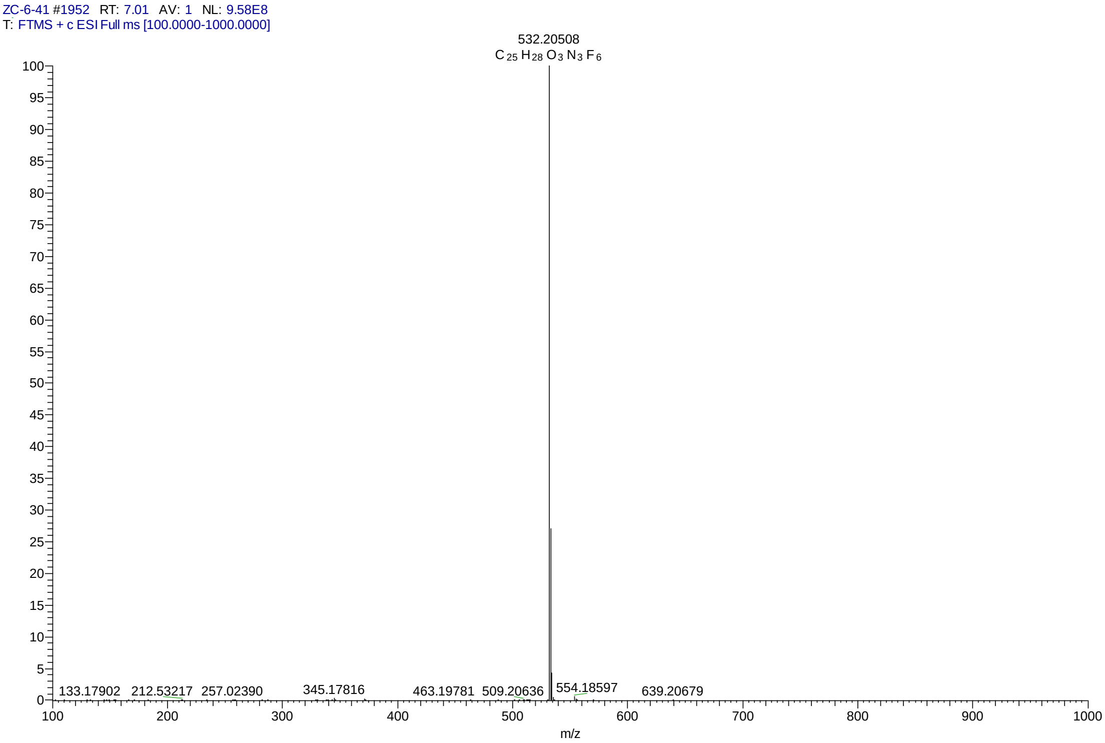

m/z	Theo. Mass	Delta (ppm)	RDB equiv.	Composition
532.20508	532.20294	4.03	10.5	C25 H28 O3 N3 F6

^{1}H NMR spectra of compound g24

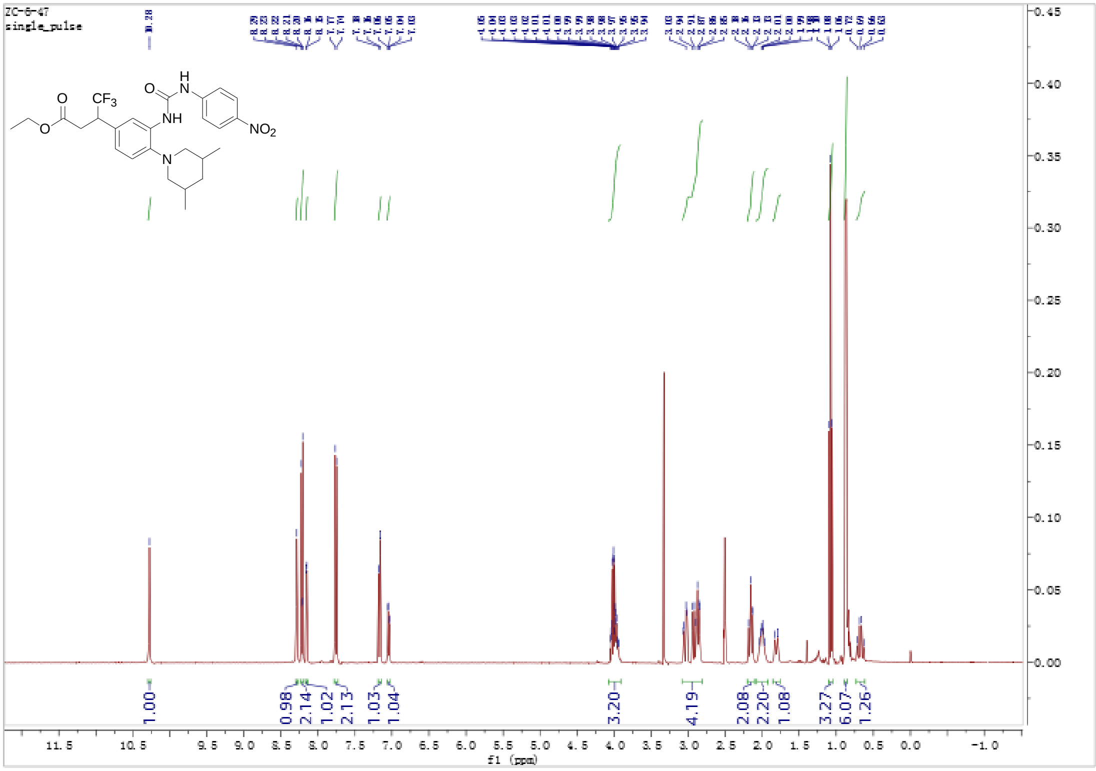

ESI-MS spectra of compound g24

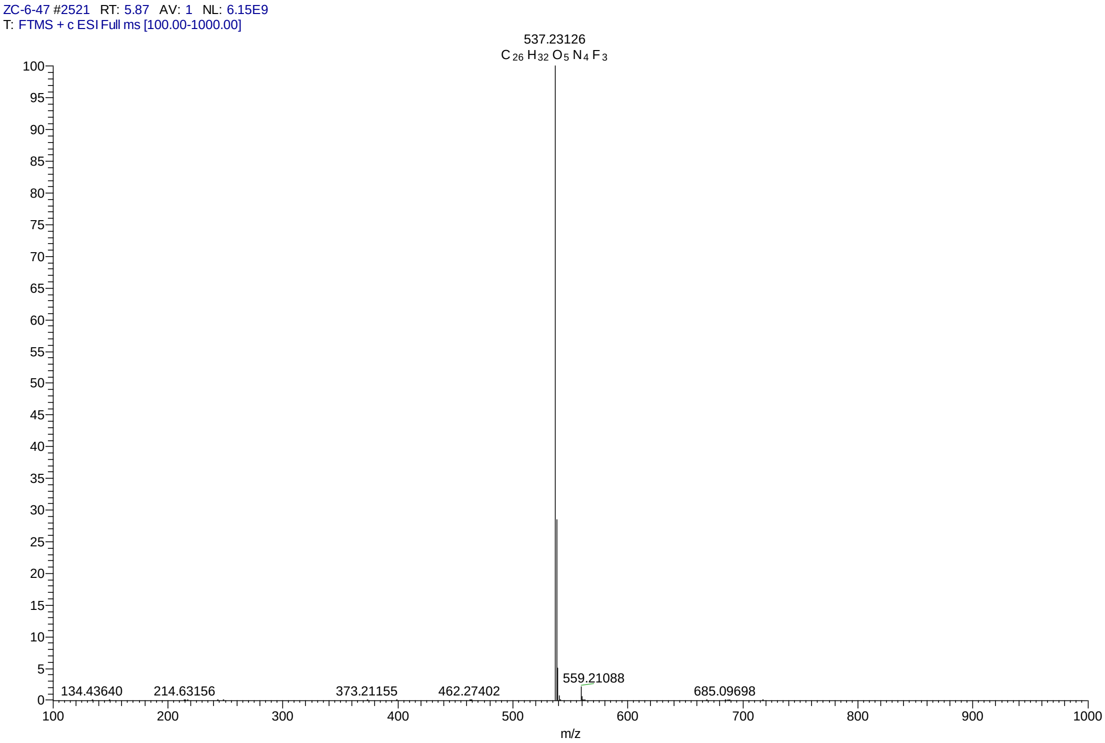

m/z	Theo. Mass	Delta (ppm)	RDB equiv.	Composition
537.23126	537.23193	-1.25	11.5	C26 H32 O5 N4 F3

^{1}H NMR spectra of compound i24

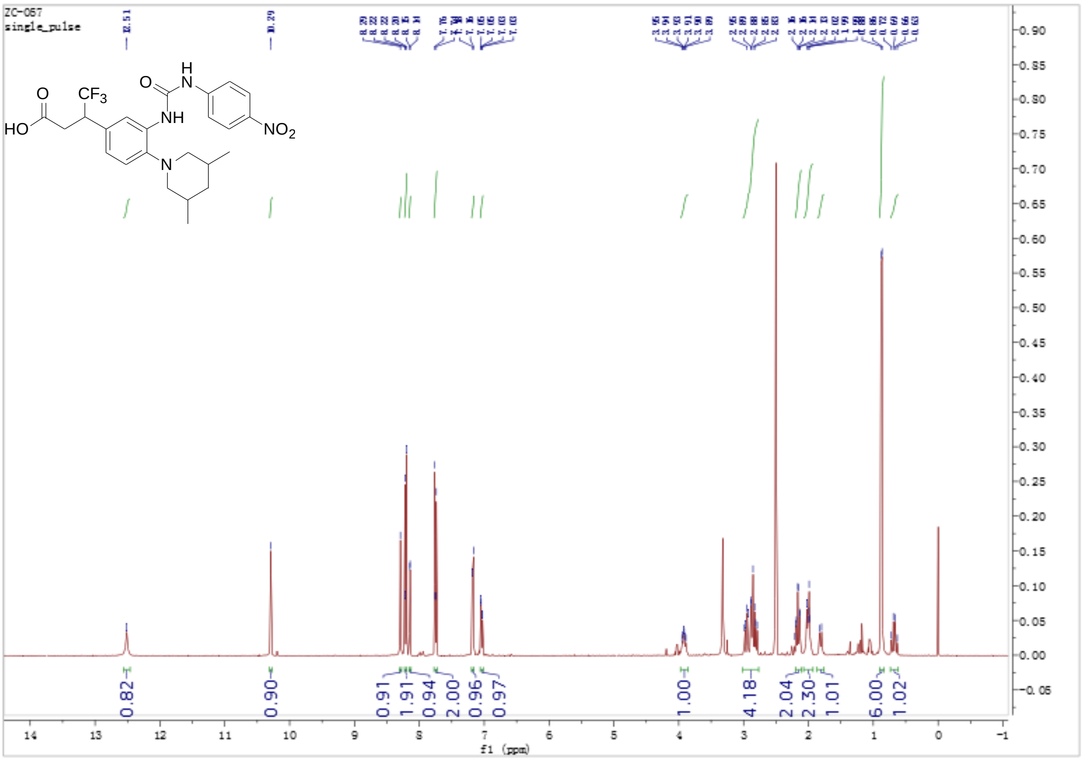

^{13}C NMR spectra of compound i24

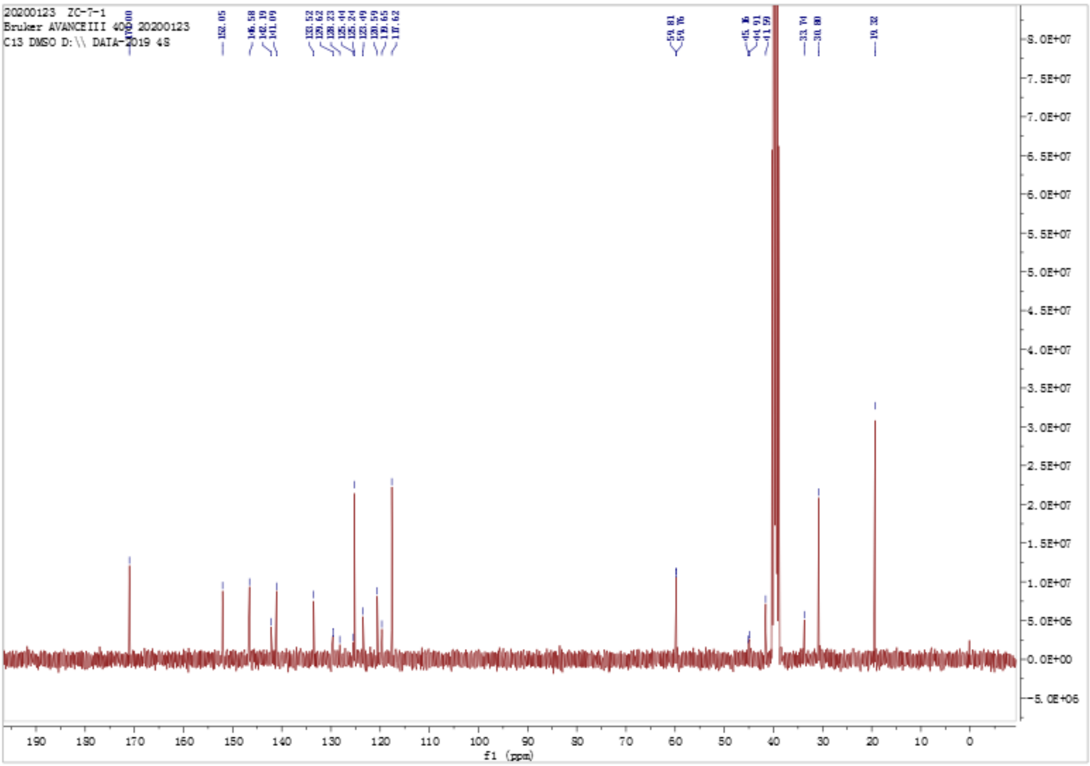

ESI-MS spectra of compound i24

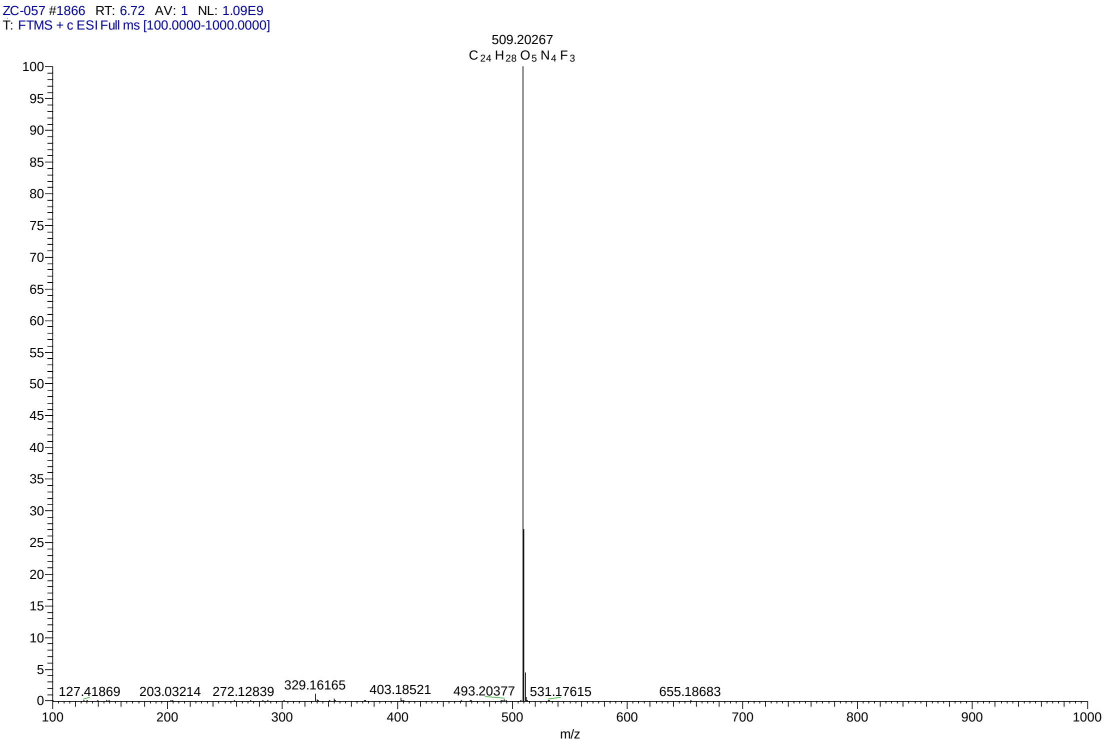

m/z	Theo. Mass	Delta (ppm)	RDB equiv.	Composition
509.20267	509.20063	4.00	11.5	C24 H28 O5 N4 F3

^{1}H NMR spectra of compound h1

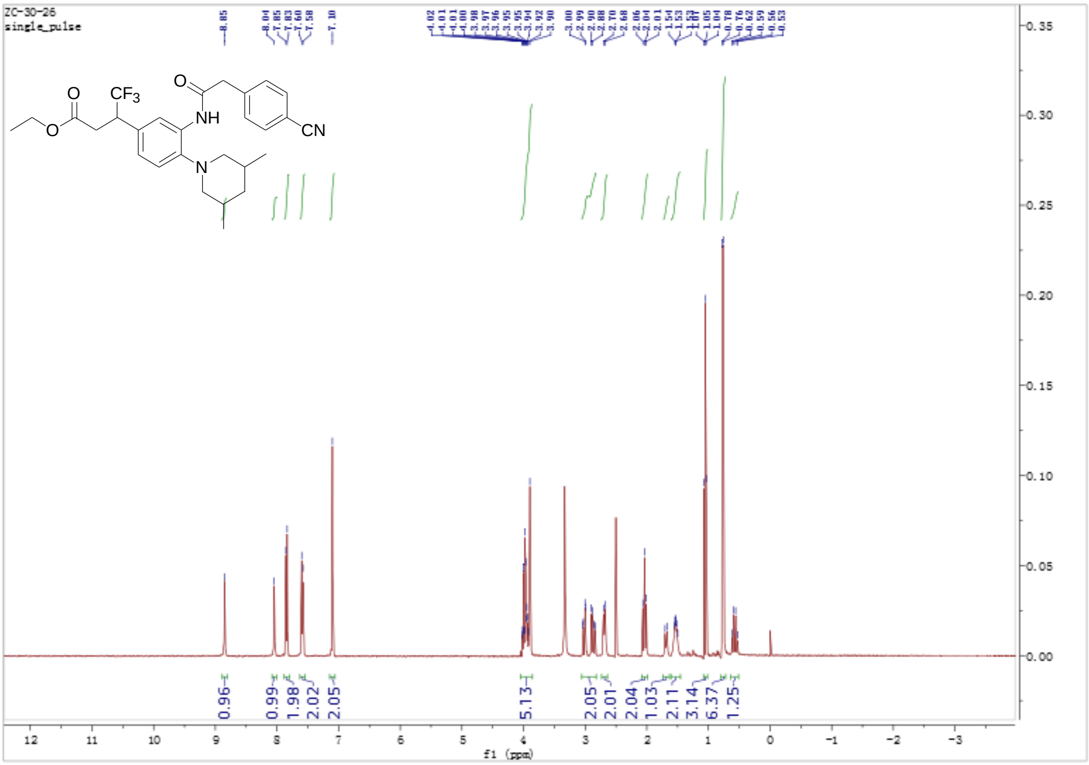

ESI-MS spectra of compound h1

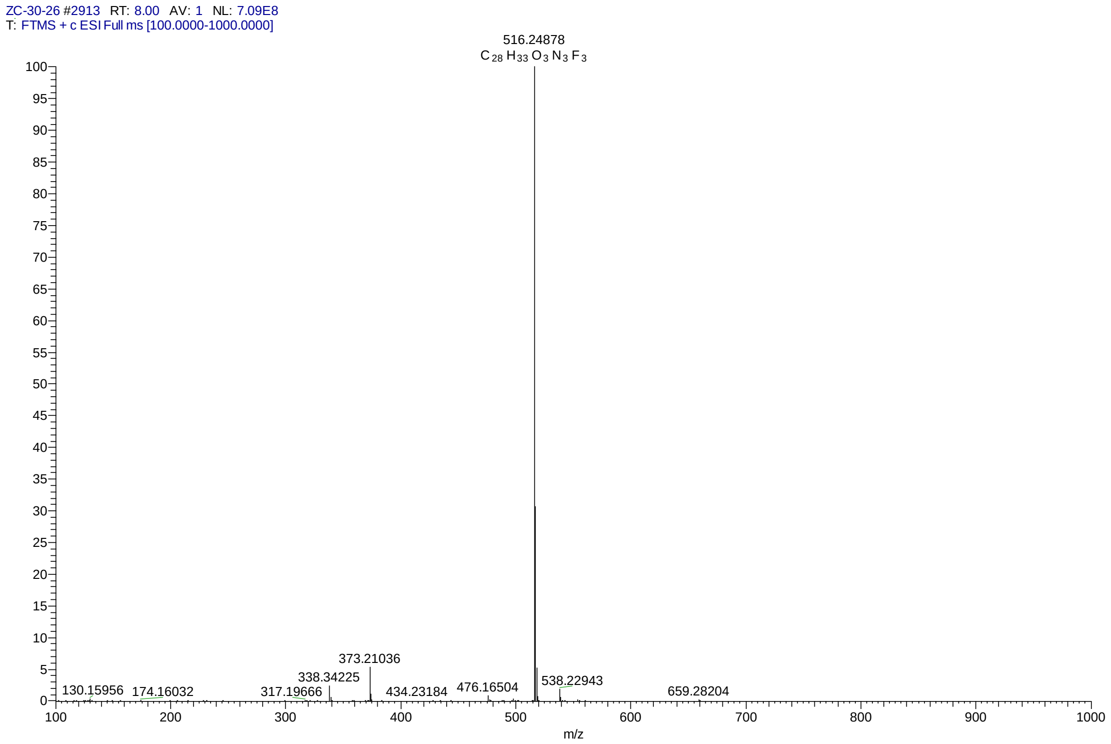

m/z	Theo. Mass	Delta (ppm)	RDB equiv.	Composition
516.24878	516.24685	3.73	12.5	C28 H33 O3 N3 F3

^{1}H NMR spectra of compound j1

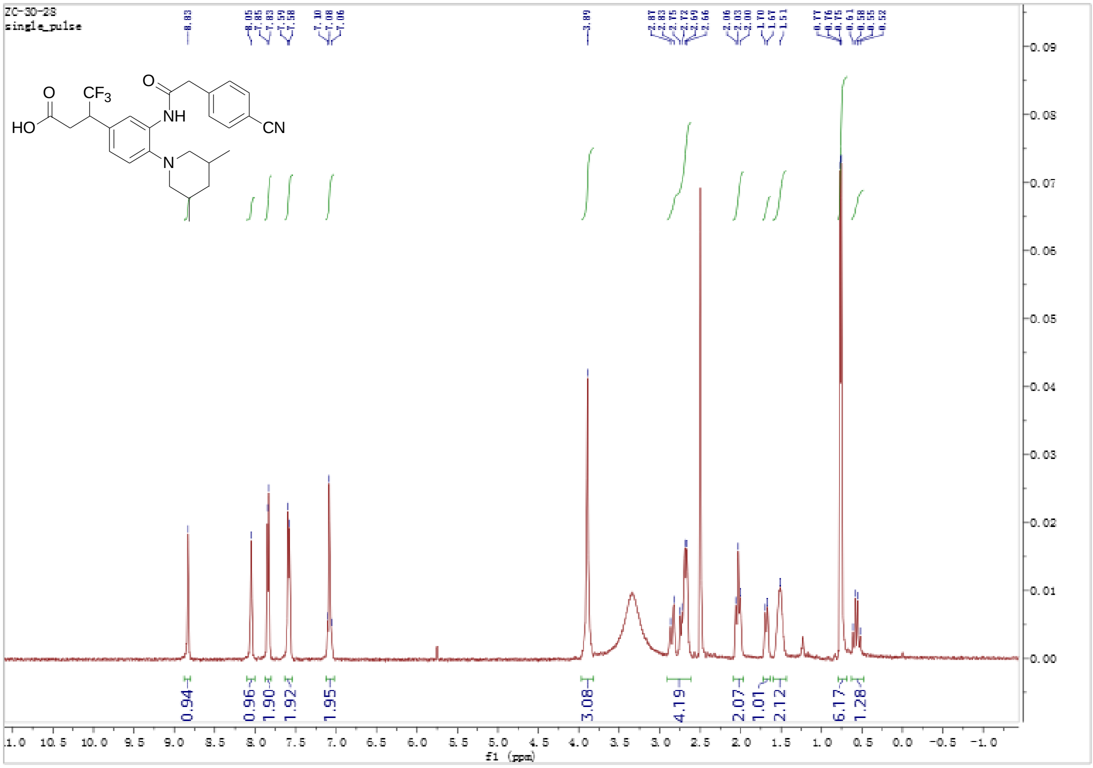

^{13}C NMR spectra of compound j1

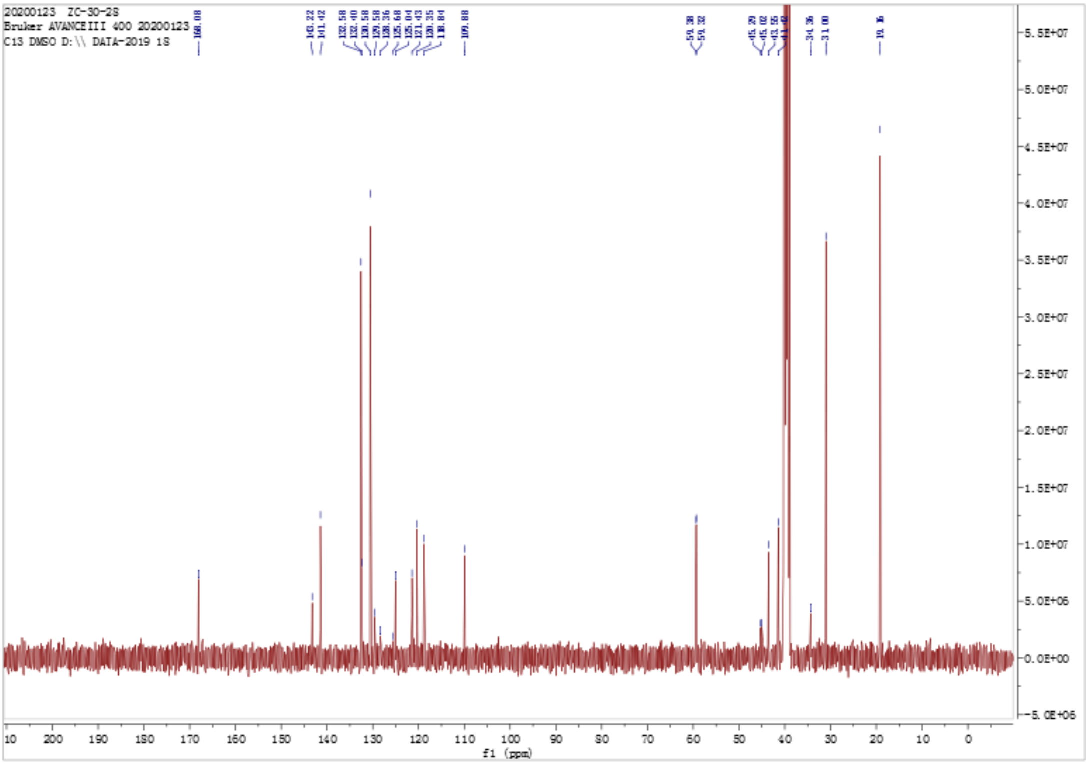

ESI-MS spectra of compound j1

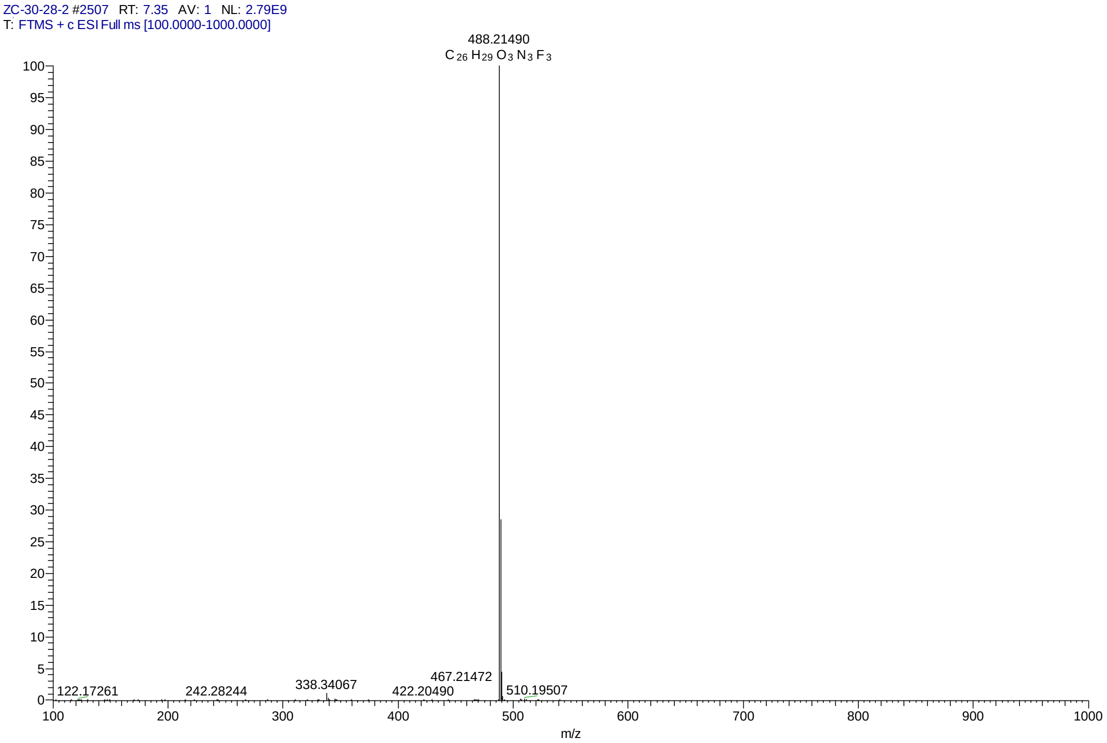

m/z	Theo. Mass	Delta (ppm)	RDB equiv.	Composition
488.21490	488.21555	-1.34	12.5	C26 H29 O3 N3 F3

^{1}H NMR spectra of compound h2

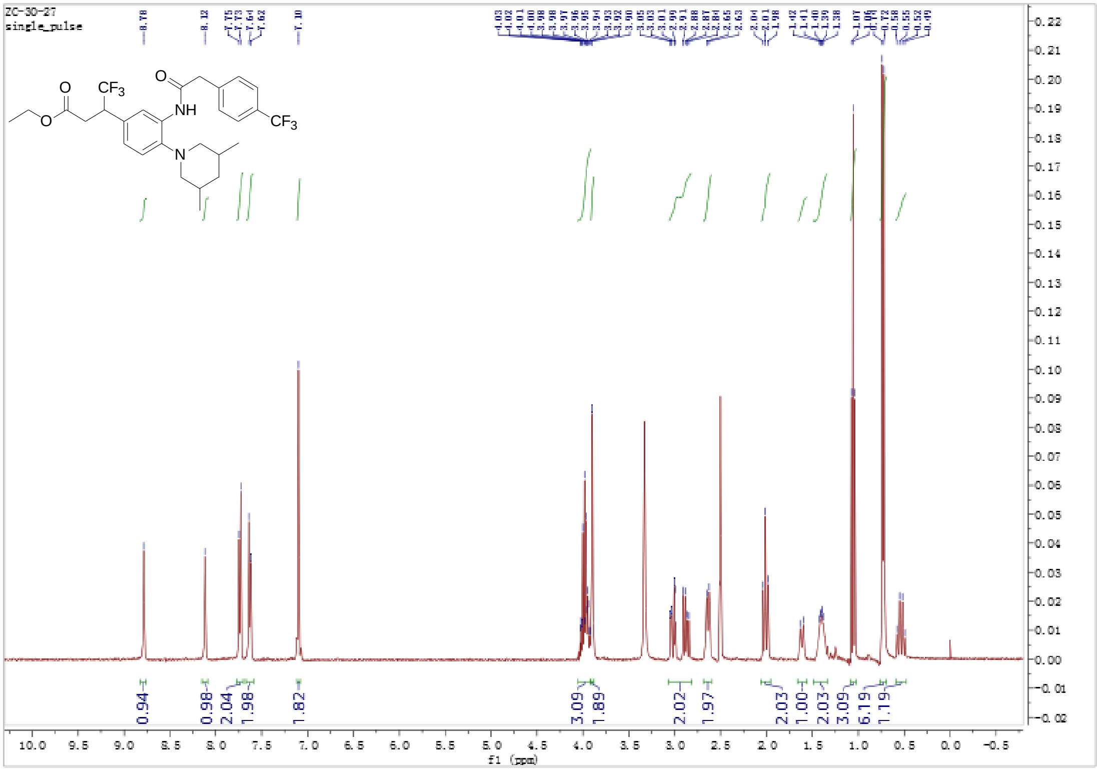

ESI-MS spectra of compound h2

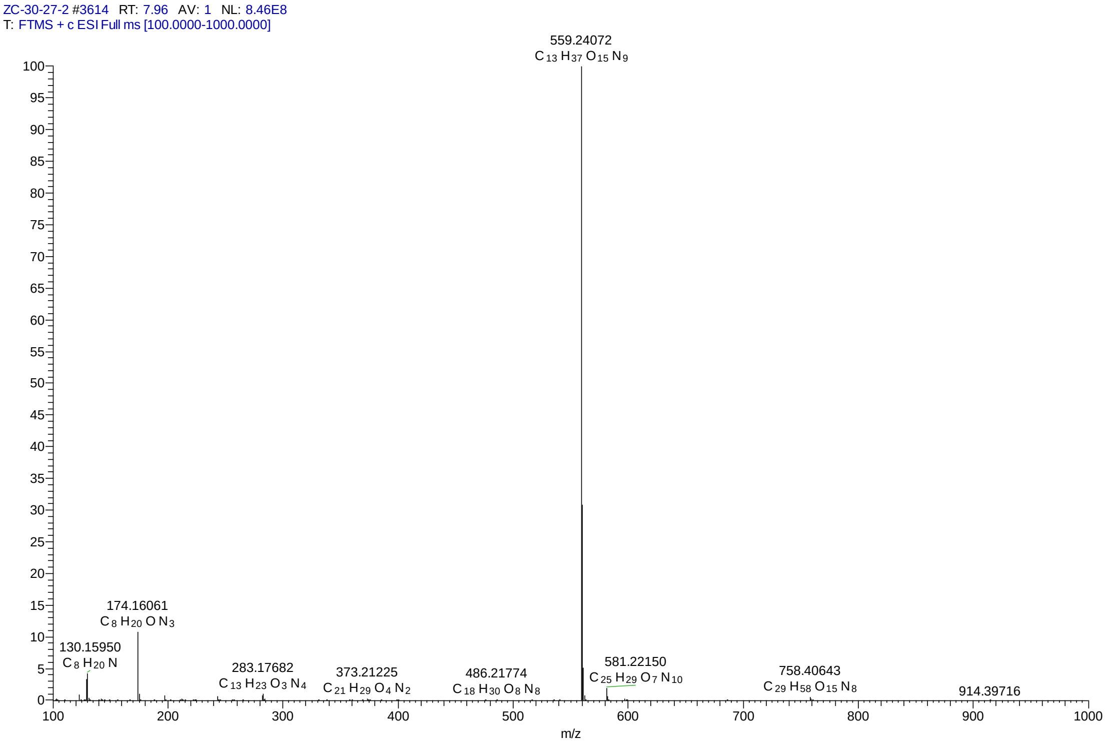

m/z	Theo. Mass	Delta (ppm)	RDB equiv.	Composition
559.24072	559.23899	3.10	10.5	C28 H33 O3 N2 F6

^{1}H NMR spectra of compound j2

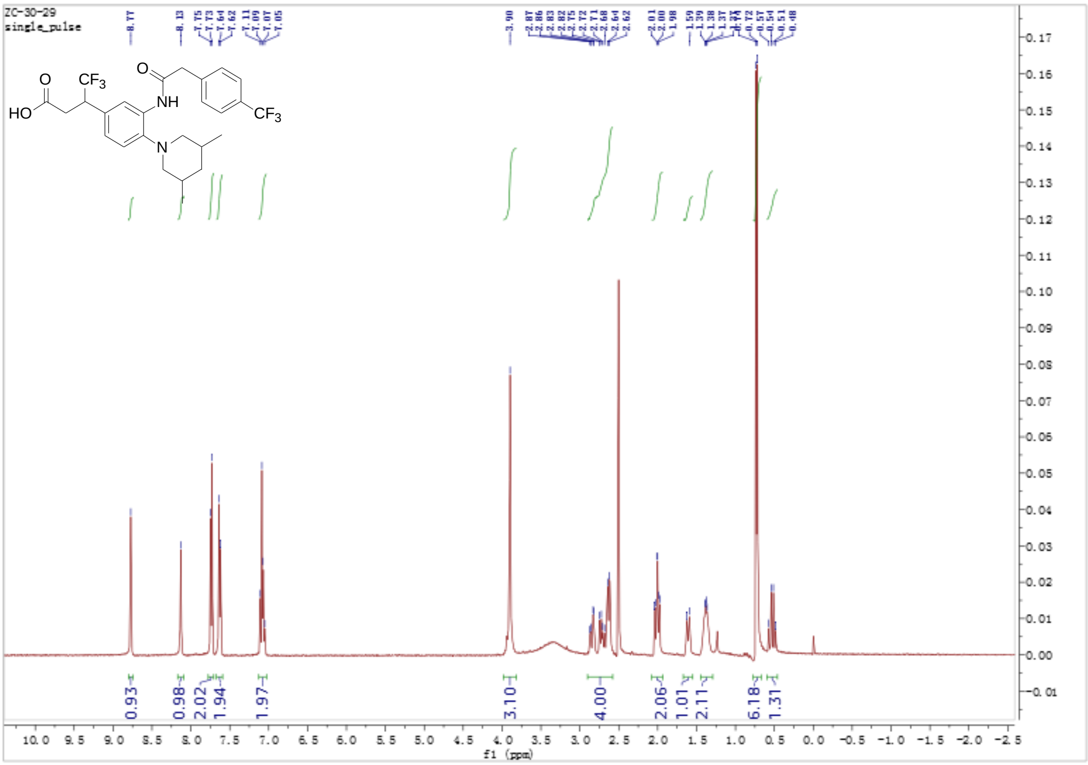

^{13}C NMR spectra of compound j2

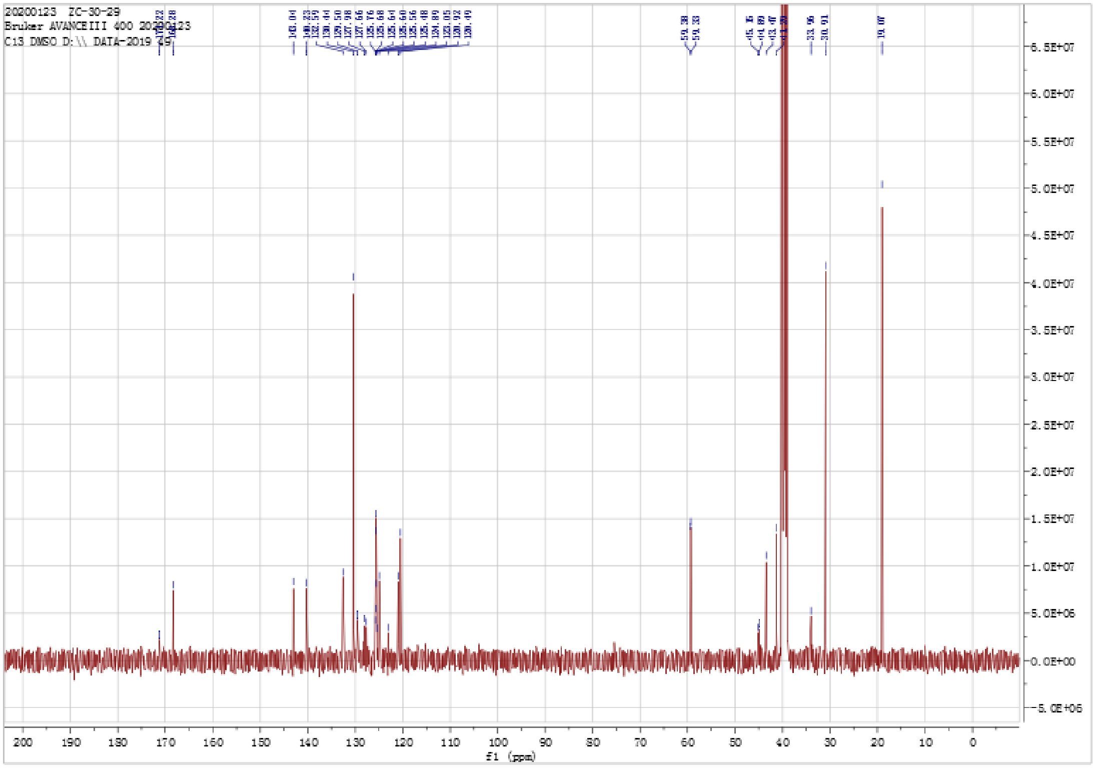

ESI-MS spectra of compound j2

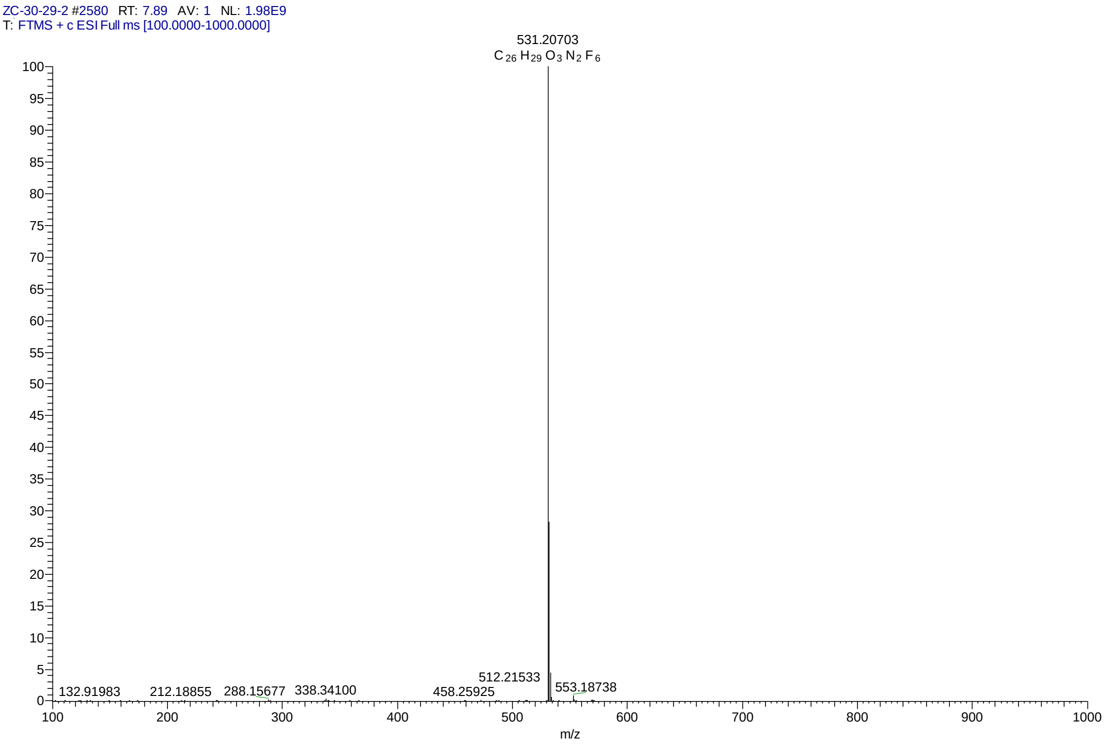

m/z	Theo. Mass	Delta (ppm)	RDB equiv.	Composition
531.20703	531.20769	-1.24	10.5	C26 H29 O3 N2 F6

^{1}H NMR spectra of compound h3

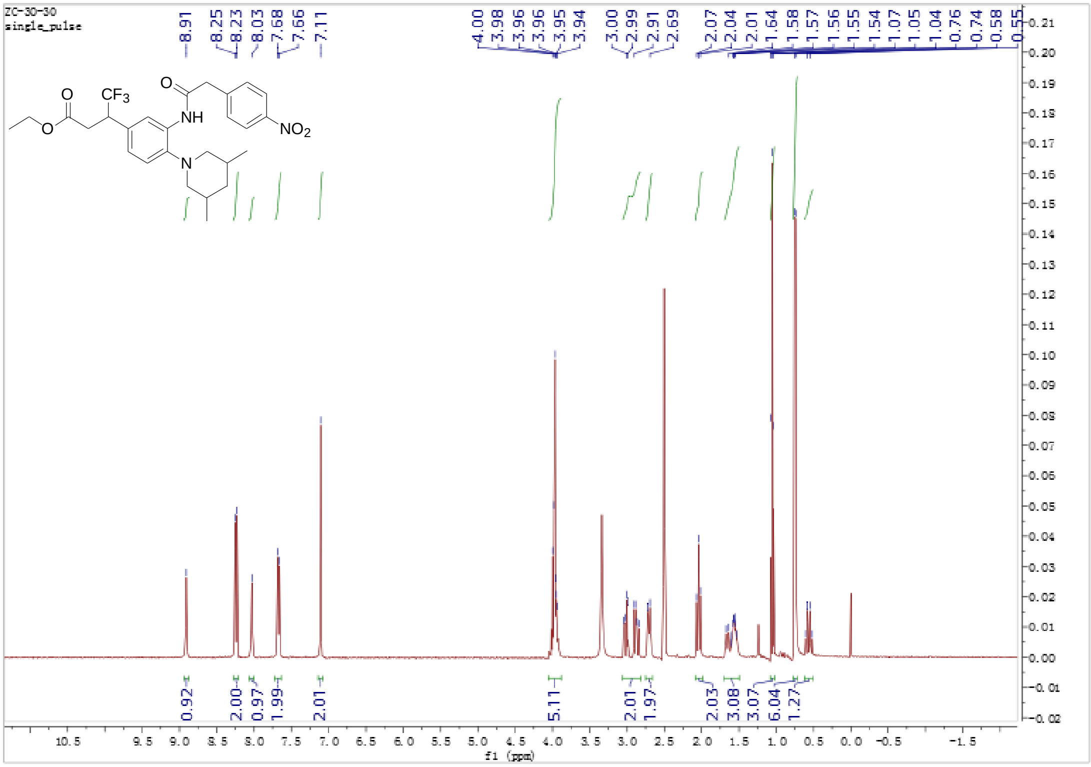

ESI-MS spectra of compound h3

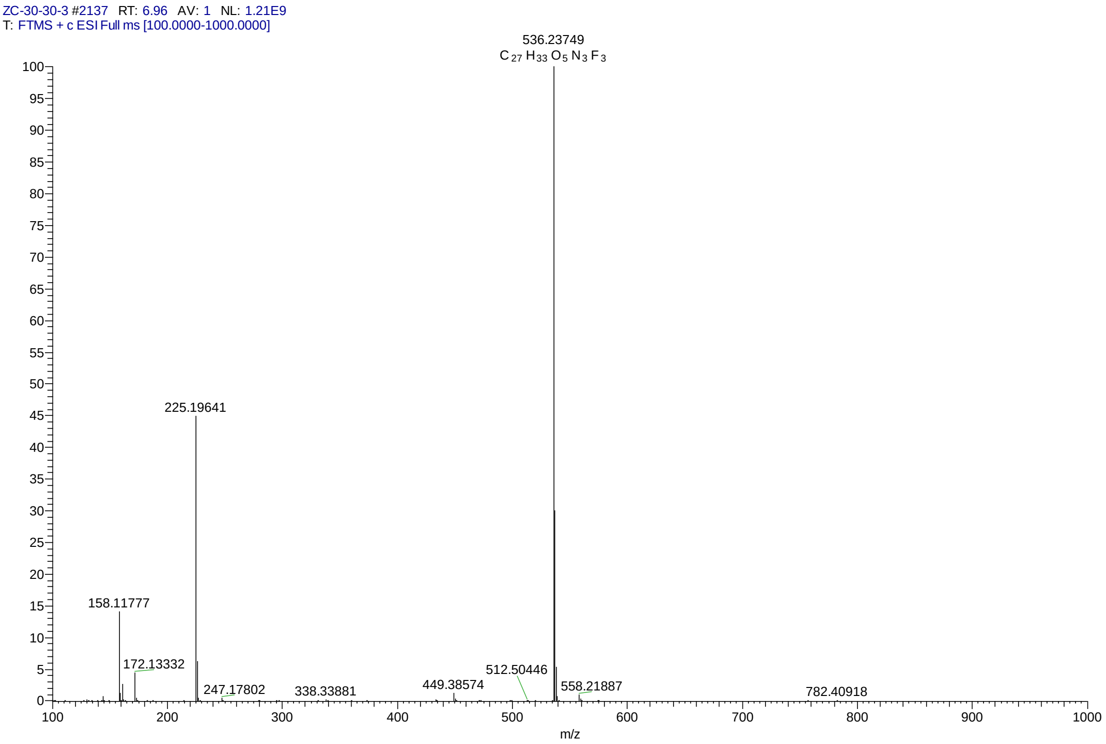

m/z	Theo. Mass	Delta (ppm)	RDB equiv.	Composition
536.23749	536.23668	1.51	11.5	C27 H33 O5 N3 F3

^{1}H NMR spectra of compound j3

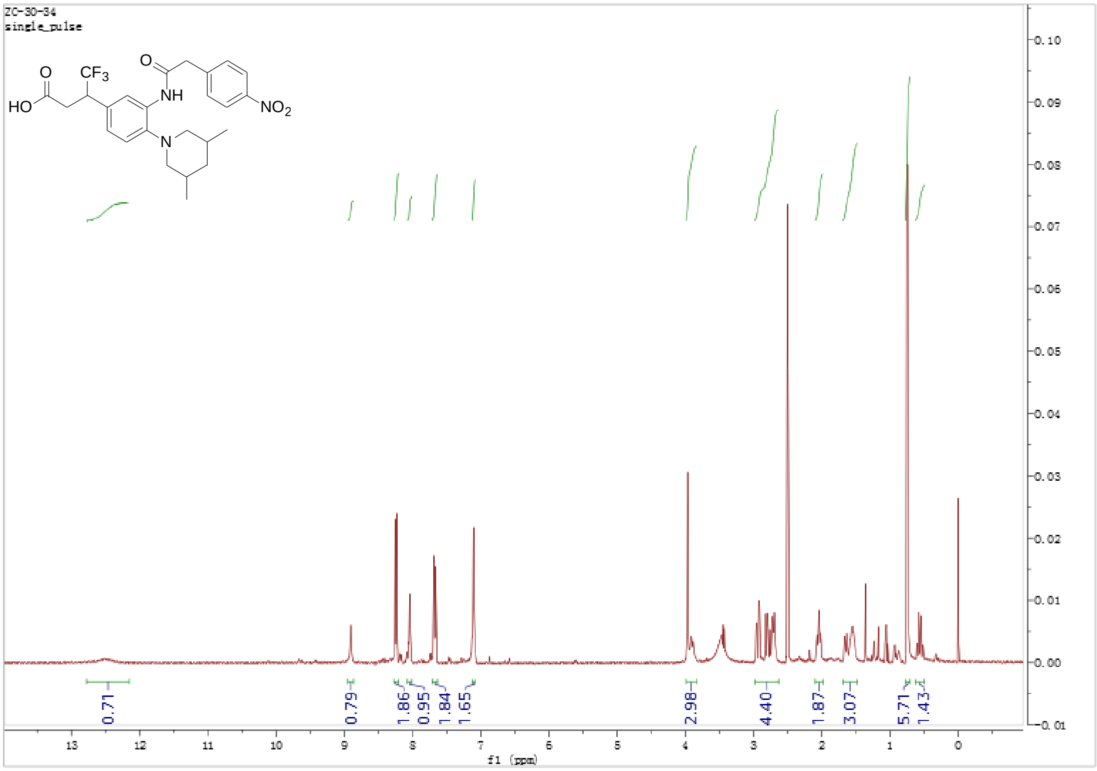

^{13}C NMR spectra of compound j3

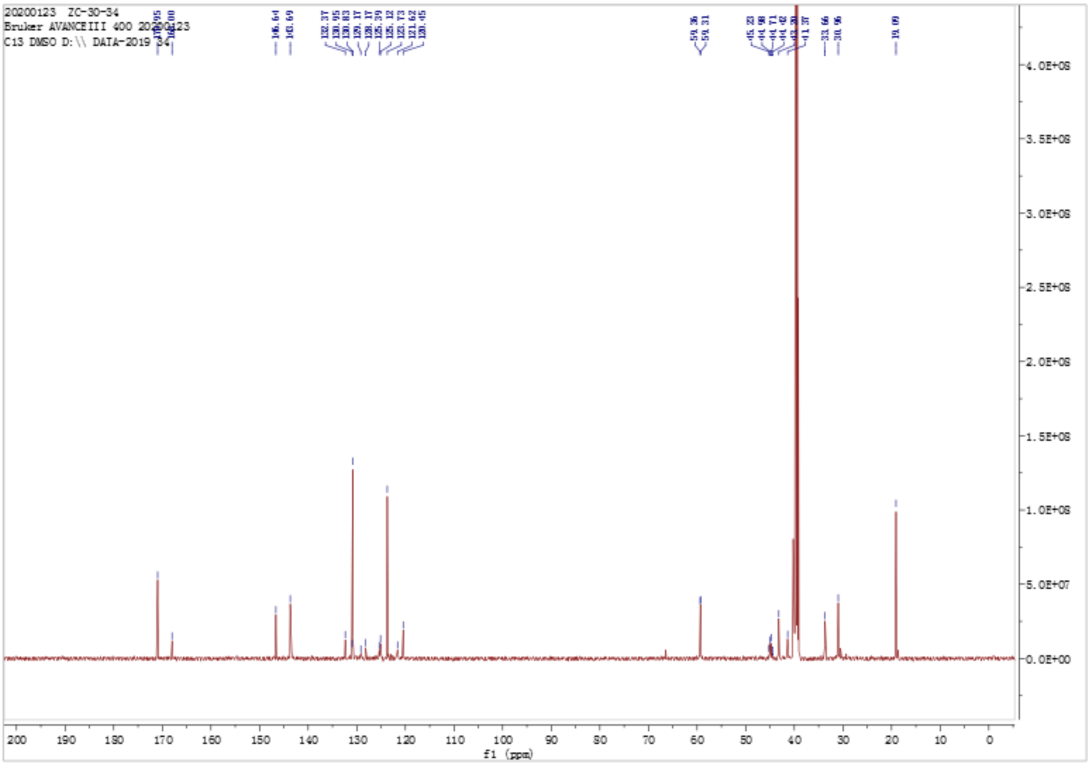

ESI-MS spectra of compound j3

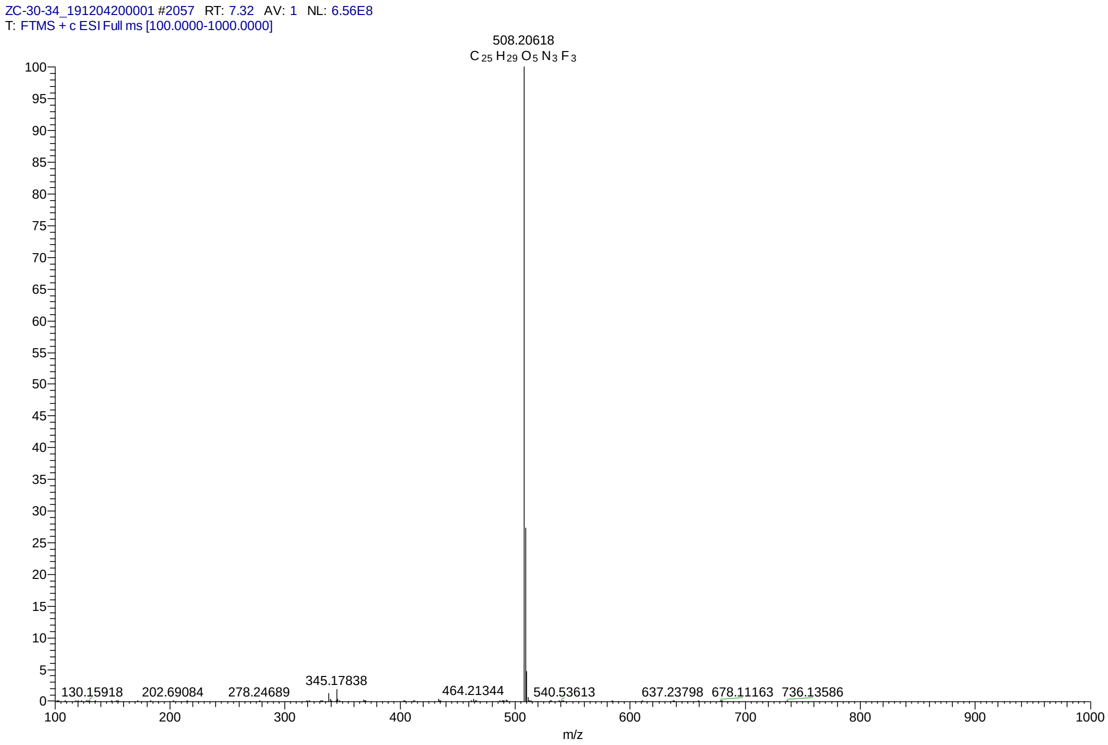

m/z	Theo. Mass	Delta (ppm)	RDB equiv.	Composition
508.20618	508.20538	1.57	11.5	C25 H29 O5 N3 F3
